# High-Throughput Identification and Characterization of LptDE-Binding Bicycle Peptides Using Phage Display and Cryo-EM

**DOI:** 10.1021/acs.jmedchem.5c00307

**Published:** 2025-10-06

**Authors:** Shenaz Allyjaun, Emily Dunbar, Steven W. Hardwick, Sarah Newell, Finn Holding, Catherine E Rowland, Megan A. St. Denis, Simone Pellegrino, Gustavo Arruda Bezerra, Nikolaos Bournakas, Dimitri Y. Chirgadze, Lee Cooper, Giulia Paris, Nick Lewis, Peter Brown, Michael J. Skynner, Michael J Dawson, Paul Beswick, Julia Hubbard, Bert van den Berg, Hector Newman

**Affiliations:** † Biosciences Institute, 12186 Faculty of Medical Sciences, Newcastle University, Newcastle upon Tyne NE2 4HH, U.K.; ‡ 643984 BicycleTx Ltd, Blocks A&B, Portway Building, Granta Park, Great Abington, Cambridge CB21 6GS, U.K.; § Department of Biochemistry, 2152 University of Cambridge, 80 Tennis Court Road, Cambridge CB2 1GA, U.K.

## Abstract

The lipopolysaccharide (LPS) transport (Lpt) system in Gram-negative bacteria maintains the integrity of the asymmetric bacterial outer membrane (OM). LPS biogenesis systems are essential in most Gram-negative bacteria, with LptDE responsible for the delivery of LPS to the outer leaflet of the OM. As an externally accessible, essential protein, LptDE offers a promising target for inhibitor development without the need for cellular penetration. However, there are no direct inhibitors of *E. coli* LptDE, and drug discovery is made challenging since it is a membrane target without a conventional active site. Here, the bicycle phage display platform was used in combination with cryogenic-electron microscopy (cryo-EM) and surface plasmon resonance to identify and map bicyclic peptide binders to *Shigella flexneri* LptDE (SfLptDE). Four distinct epitopes with unique bicycle molecule binding motifs were identified across the SfLptD β-barrel. This method represents a streamlined workflow for the identification and prioritization of hit molecules against LptDE.

## Graphical abstract

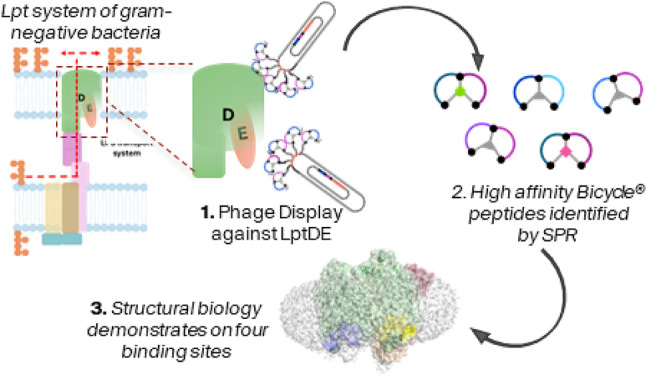

## Introduction

Multidrug resistant bacteria present an increasingly prominent and widespread challenge to public health, in the form of eroding efficacy of currently available antibiotics for treating dangerous infections.^1,2^ This has prompted urgent calls for the development of new antimicrobial agents, displaying innovative chemotypes and mechanisms of action.^3,4^ In Gram-negative bacteria, an asymmetric outer membrane (OM) is present with a protective layer of lipopolysaccharides (LPS), which serves as a permeation barrier for many existing antimicrobial compounds.^5,6^

The seven-part LPS transport system (LptA-G) maintains OM integrity by transporting LPS from the bacterial cytoplasm across the periplasm and then delivering it through the OM to the cell surface.^7–10^ LPS is first extracted from the inner membrane by the LptB_2_FGC complex, then crosses the periplasm on a protein bridge composed of LptA, before reaching the outer-membrane embedded LptDE complex (Figure 1A).^8,11–14^ LptDE is a two-protein plug and barrel complex that spans the OM, made up of the β-barrel LptD, coupled with an associated lipoprotein LptE. The complex translocates LPS from the periplasmic space to the outer leaflet of the OM.^7,8,15–20^ LptD consists of two domains: an N-terminal β-jellyroll domain situated in the periplasm and a β-barrel integrated in the OM bilayer.^7,16,20,21^ LptE resides in the LptD β-barrel in a “plug and barrel” architecture and is involved in the assembly and stability of LptD (Figure 1C).^22,23^ The LptDE complex is an attractive target for novel antibiotic development as it is essential in most Gram-negative pathogens,^24–27^ and has a surface-exposed location.^28,29^

**Figure 1:**
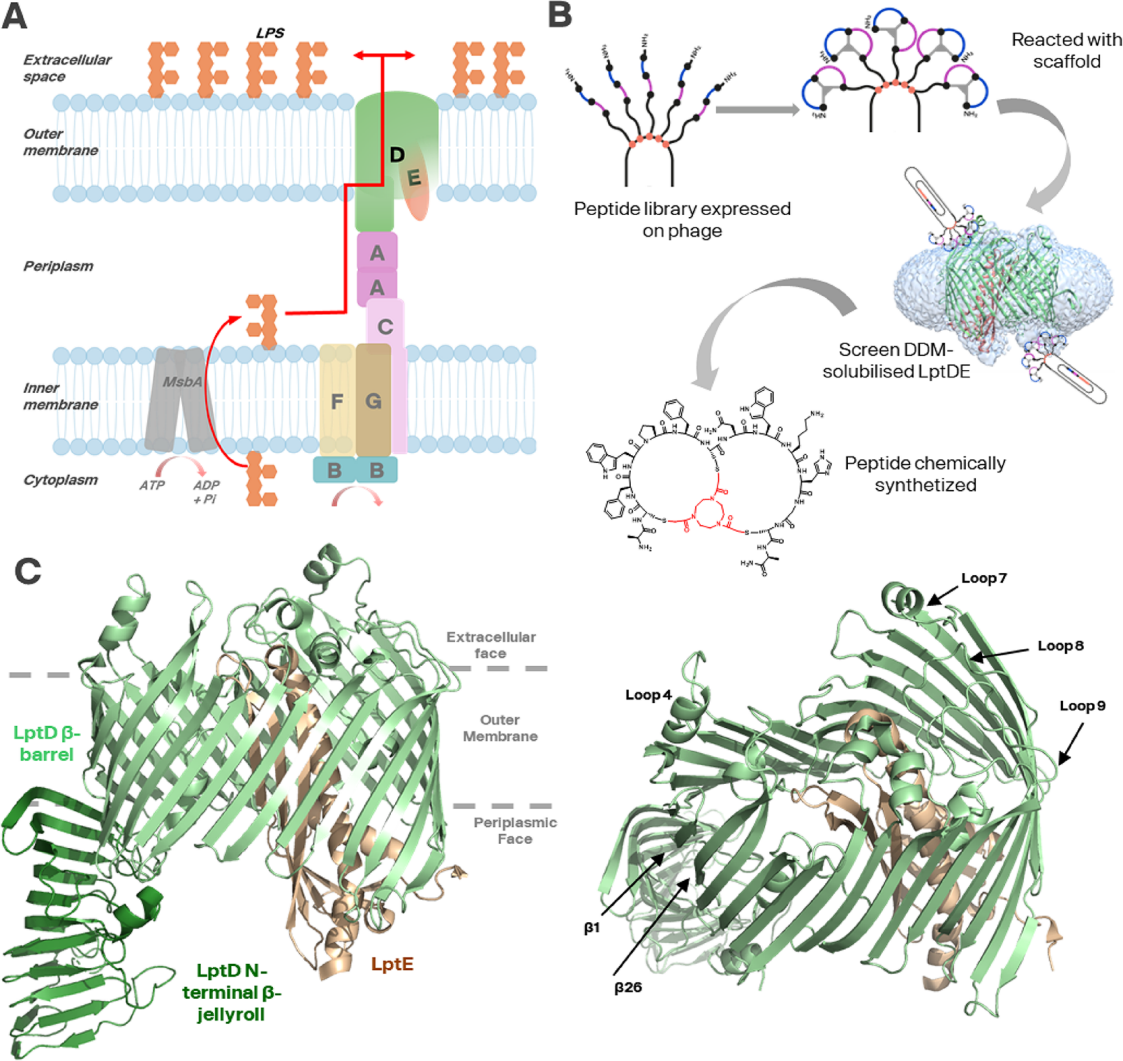
Phage display of Bicyclic peptides against LptDE from*Shigella flexneri*. (A) LptDE is the most downstream member of the Lpt family of proteins, which delivers LPS from the inner member to the outer membrane in Gram-negative bacteria. It is a complex of LptD and LptE. (B) Target-based drug discovery on the Bicycle platform uses phage expressing linear peptides which are subsequently “scaffolded” (scaffold shown in red) to create Bicycle molecules displayed on phage particles. These are screened against the LptDE complex by sequential rounds of affinity pulldown, then chemically synthesized (compound **12** is shown). Not to scale. (C) Crystal structure (PDB ID:4Q35, ref 16) of the SfLptDE complex, including an N-terminal β-jellyroll that forms a V-shaped hydrophobic groove extending into the periplasm that forms contacts with LptA. The outer-membrane associated β-barrel is made up of 26 antiparallel β-strands (β1−β26). These β-strands are connected by turns on the periplasmic side and extended loops on the extracellular face that fold over the lumen of the barrel and require rearrangement to allow LPS molecules to reach the external surface. LptE is tightly bound in the LptD β-barrel.

Previous attempts to identify LptDE inhibitors have been largely unsuccessful. A large antibody screening campaign against LptDE undertaken by Genentech found only nonfunctional α-LptD antibodies. This was hypothesized to be due to insufficient accessibility of essential regions of the protein.^28^ Ribosome and phage display was used to generate several high affinity Pro-macrobodies against *Neisseria gonorrheae* LptDE, but these do not have associated antimicrobial activity.^18^ In contrast, the most promising antimicrobial compound targeting LptDE is the *Pseudomonas aeruginosa*-specific macrocyclic peptide Murepavadin (POL7080), which was discovered as a derivative of the naturally occurring antimicrobial peptide protegrin 1.^27,30^ Murepavadin binds to a periplasmic domain unique to *Pseudomonas aeruginosa* LptD. Molecules that target sites on the OM itself (such as LptDE) do not need to be able to permeate the cell, circumventing a significant challenge in antimicrobial drug development. Phage display was used to discover Bicycle molecules against this challenging target.

Bicycle molecules are bicyclic peptides, formed by the reaction of a trimeric scaffold with 3 cysteines in a linear peptide, resulting in 2 loops.^31^ The conformational restraints imposed by cyclization can result in a higher target specificity and affinity than equivalent linear peptides, with an increased resistance to proteolytic degradation.^32–34^ The Bicycle phage display platform provides an efficient methodology to identify target reactive cyclic peptides in a high throughput manner. These peptides occupy chemical space which could be considered analogous to naturally occurring antimicrobials. However, in contrast to naturally occurring antimicrobials, which are notoriously difficult to chemically modify, Bicycle molecules are fully chemically tunable, which allows them to be optimized as therapeutics.^31,35^

Cryo-electron microscopy (cryo-EM) can elucidate biological structures like membrane proteins at high resolution in close-to-native states, making it an important tool for enabling structure-based drug design.^26,36,37^ Some technical challenges still remain – primarily in the need for high-quality samples and the typically lower throughput when compared to X-ray crystallography. However, continuous advancements in grid preparation, imaging, and processing technologies have revolutionized cryo-EM to enable near-atomic resolution for increasingly complex targets. Recently, the development of molecules against historically challenging membrane protein targets, such as G-protein coupled receptors (GPCRs) and ion channels, has been accelerated through increased use of cryo-EM.^38–40^

Here, a methodology is described which combines the output from the Bicycle phage display platform with surface plasmon resonance (SPR) assays, followed by medium-throughput cryo-EM to identify and triage Bicycle molecules binding to *Shigella flexneri* (Sf) LptDE. SfLptDE was chosen as it has high sequence similarity to the *E. coli* LptDE protein (4 residue differences between the LptDE of the two species, Figure S1) and sufficient amounts of protein can be conveniently produced.^16^ It was assumed that Bicycle molecules discovered against SfLptD would be able to bind to *E. coli* LptD as 2 out of 3 of the sequence variants are buried in the membrane and inaccessible, and the sequence variants in LptE are in the disordered C-terminal region (Figure S1).

Multiple peptides were identified binding to SfLptDE with affinities better than 1 μM, which were subsequently mapped to multiple competition “bins” by SPR. Binding modes were described by cryo-EM, resulting in 8 high-resolution models of SfLptDE in complex with Bicycle molecules at 4 different binding sites. The cryo-EM data collection strategy was optimized to generate maps for up to 3 structures per 24 h of data collection at resolutions high enough to confidently identify Bicycle molecule binding epitopes, even within these challenging small membrane protein samples. Altogether, this represents a robust approach to efficiently prioritize and classify LptDE binding molecules and the rapid localization of their binding sites.

## Results and Discussion

### Hit Identification by Phage Panning

LptDE from *Shigella flexneri* (SfLptDE)^16^ was co-overexpressed and purified from *E. coli* BL21 (DE3) cells and reconstituted into micelles of *n*-dodecyl-β-d-maltose (DDM) detergent. Two constructs of SfLptDE were used in this study: a full-length construct (SfLptDE_FL_) including both the membrane-associated β-barrel and the periplasmic N-terminal β-jellyroll domain of LptD (LptD residues 25–784, LptE residues 1–175); and a truncated construct (SfLptDE_T_) consisting of the β-barrel of LptD only (SfLptD residues 204–784, LptE residues 19–193). During the expression of the FL and truncated proteins in the *E. coli* expression system, the signal peptides of both LptD and LptE were cleaved (Figure S1). After purification, both complexes were chemically biotinylated at primary amines via the use of an amine-reactive biotin ester.

Biotinylated SfLptDE complexes were used to conduct phage panning against libraries of cyclized peptides, as described in previous studies.^31,35,41^ Phage libraries consisted of peptides between 11 and 17 amino acids in length, cyclized via 3 thioether bonds to seven different small molecule “scaffolds”. Four sequential rounds of selection were carried out, with progressive lowering of SfLptDE target concentrations to increase the stringency of phage binding.

Two selections were conducted in this study: one small-scale pilot screen against SfLptDE_FL_, and a second, larger screen against SfLptDE_T_. The truncated form of the protein was chosen for large scale investigation with the aim of reducing the pull-down of molecules binding to the periplasmic N-terminal region of SfLptD and to favor the identification of external face binders. Both forms of the protein were highly tractable to phage display, with at least 4000 unique peptide sequences, representing a diverse array of sequence motifs, identified by next generation sequencing. From the two selections, 76 Bicycle peptides were selected based on their sequence diversity, frequency of observation and, in cases where a monoclonal phage was isolated, the presence of a positive signal in AlphaScreen (Figure S2). These peptides were synthesized using solid phase peptide synthesis (SPPS) to enable biophysical characterization (Figure 2).

**Figure 2:**
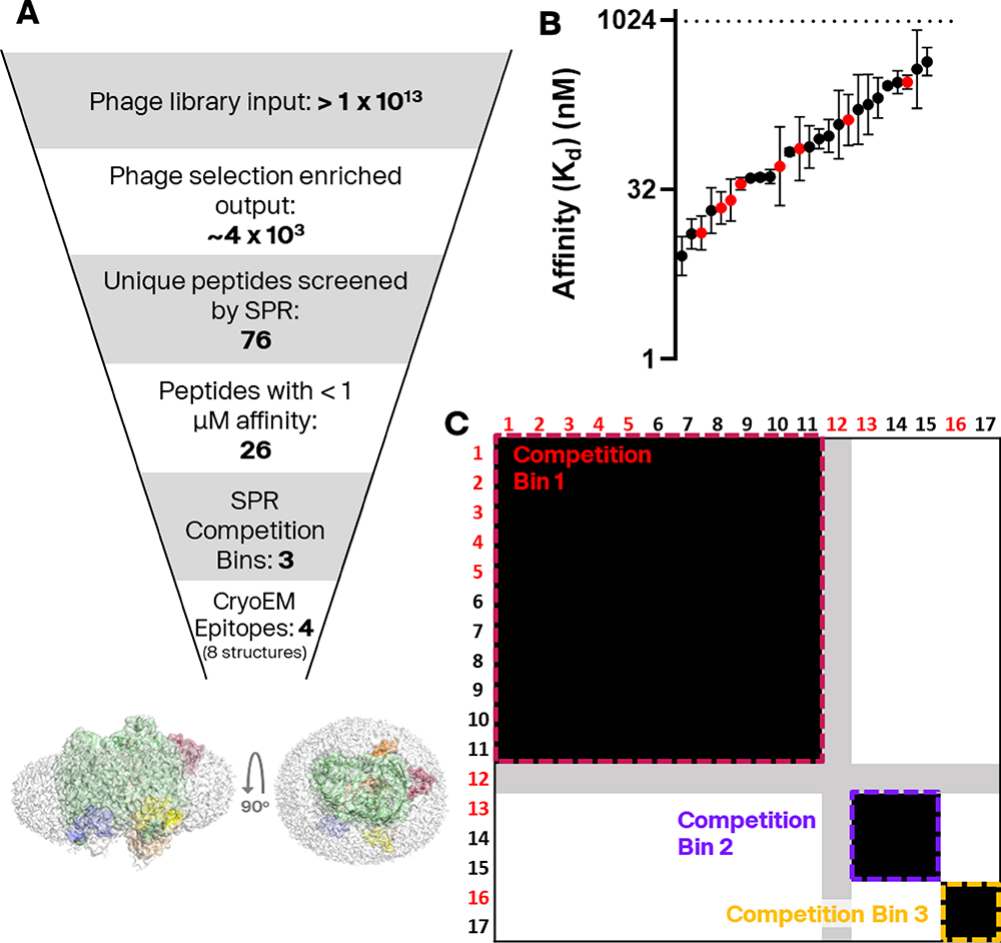
**S**creening cascade overview and SPR hits. (A) Overview of the stages in the screening cascade. Two views of Bicycle molecules binding to the 4 epitopes on SfLptDE (surrounded by a micelle) are shown. “Competition bin” and “epitope” are defined in the text. (B) Affinities of 76 peptides were measured in an SPR assay against LptDE_FL_. The affinities for 26 peptides are shown. An additional 50 peptides were determined to be nonbinders or bound in a nonspecific manner. The weakest affinity that could be reliably measured in this assay was 1 μM. Compounds highlighted in red were analyzed by CryoEM. Error bars are geometric standard deviations of at least 2 biological replicates. (C) Heatmap view of the SPR competition binning experiment. Each row/column represents the interaction profile of one peptide. A peptide–peptide interaction is described as competitive (black), noncompetitive (white) or ambiguous (gray). Numbers of replicates for each interaction are shown in Figure S3E. Interactions with Compound **12** could not be unambiguously assigned because this compound does not appear to compete with itself, which is a critical positive control. An example sensorgram of this is shown in Figure S3D.

### Biophysical Analysis of Hit Peptides

A surface plasmon resonance (SPR) assay^18,28^ was subsequently used to determine the binding affinities of the Bicycle molecules to SfLptDE_FL_. SfLptDE gives reproducible traces that typically fit well to a 1:1 binding equation model, making SPR a suitable primary assay to determine Bicycle molecule-protein interactions. Of the 76 different Bicycle molecules tested, 26 molecules had better than 1 μM affinity, with the highest affinities measuring ∼ 10 nM (Figure 2C and Table 1).

**Table 1:** Characteristics of the Bicycle Peptides Identified in Complex with SfLptDE in This Study

compound	sequence^a^	scaffold^b^	peptides in competition bin^c^	affinity (p*K*_d_)^d^	affinity (*K*_d_, nM)^e^	binding face of SfLptDE^f^
**1** ^g^	ACKWENDIWHCM **WMD** CA	TBAZ	11	7.9 ± 0.15 (3)	13	extracellular facing, partially buried in micelle
**2** ^g^	ACRAKCDWFS **WLD** DCA	TCTZ		7.6 ± 0.19 (3)	26	
**3** ^g^	ACKKGCDWLFL **WHD** DCA	TCTZ		6.9 ± 0.23 (4)	133	
**4** ^g^	ACDPWWQFN **WCD** QDHCA	TBCU		7.6 ± 0.14 (4)	22	
**5** ^g^	ACWHW**WL** E **E**DCDKKECA	TATB		6.5 ± 0.06 (3)	287	
**6**	ACPFWWQLN **WCD** ADWCA	TBCU		7.4 ± 0.01 (2)	40	
**7**	ACPWNENIWYCM **WED** CA	TBAZ		7.0 ± 0.09 (2)	90	
**8**	ACKDRCDWFS **WQD** ECA	TCTZ		6.8 ± 0.31 (4)	164	
**9**	ACKQHWDWYCV **WMD** ECA	TBAZ		7.4 ± 0.06 (2)	42	
**10**	ACHKSCDWNLL **WLD** DCA	TCTZ		7.1 ± 0.19 (3)	76	
**11**	ACADPWSCF **WSD** WTCA	TBCU		6.7 ± 0.18 (2)	208	
**12** ^g^	ACFWPFCNWKHGCA	TBAZ	1	7.3 ± 0.35 (5)	51	extracellular facing, partially buried in micelle
**13** ^g^	ACS **D** **FMDWFYCD** GYPCA	TATB	3	7.1 ± 0.28 (3)	74	periplasmic facing, partially buried in micelle
**14**	ACT **DFMDWLYCE** HYTCA	TATB		7.8 ± 0.20 (3)	21	
**15**	ACQ**D** FMDYFFCDFQMCA	TATB		6.7 ± 0.27 (3)	182	
**16** ^g^	ACE **WCIFW** YDPKLCA	TSTA	2	7.4 ± 0.05 (3)	36	periplasmic facing, partially buried in micelle
17	ACDP **WCIFW** MPCA	TATB		8.1 ± 0.17 (2)	8	

a Where a sequence motif was identified for an epitope of peptides it is shown in bold.

b Scaffold structures are shown in Table S6.

c The number of peptides with confirmed SPR binding identified in this by SPR competition binning (Figure 2C). Compound **12** could not be unambiguously assigned to any competition bin, but structural studies showed it to be binding in a different epitope to all other peptides tested.

d Affinity measured by SPR represented as a p*K*_d_: average of −log_10_(*K*_d_) ± standard deviation of −log_10_(*K*_d_). Replicate numbers are shown in brackets.

e Affinity measured by SPR, represented as antilog geometric means.

f Binding face of those peptides tested by CryoEM.

g Compounds further analyzed by cryo-EM.

A subset of the SfLptDE-binding Bicycle molecules was taken forward into a competition binning assay using SPR with an A–B–A injection system. This system allowed the simultaneous engagement of two Bicycle molecules to be determined, indicating whether they competed for the same or different “competition bins”. Peptides binding to the same competition bin may be binding to the same location on the protein, but other factors (such as allosteric interaction) could instead be responsible for the observation. Therefore, the term competition bin indicates binding sites derived from SPR studies and “epitope” for those identified by structural biology. Alignment of peptide sequences in each competition bin revealed conserved motifs, indicating a relationship between sequence and binding location (Figure 2C and Table 1).

### Cellular Assays

Without a convenient biochemical assay of LptDE activity, functional inhibition could only be tested by bacterial cell growth inhibition assays in the presence of each molecule. The antimicrobial activity of each of the Bicycle molecules was tested against a panel of weakened *E. coli* strains. While the protein was from *Shigella*, this was used primarily as a model for the *E. coli* protein. In order to increase our ability to detect weak effects on bacterial growth we selected a panel of *E. coli* mutant strains with attenuated OMs via knockouts of nonessential OMP protein biogenesis proteins (Δ*bamB*, Δ*bamC*, Δ*bamE*, Δ*surA*),^42^ weakened LptDE complex (Δ*lptM* ^42^), attenuated LPS production (Δ*waaD*).^42^ BamB, BamC and BamE are nonessential members of the BAM complex, alongside the chaperone protein SurA. BAM folds OMPs into the OM, and knockout of individual components of the complex have been shown to cause defects in outer membrane assembly if the minimal function of the complex is not met.^43^ LptM is a nonessential member of the Lpt pathway which has recently been shown to aid the assembly of LptDE to ensure correct disulfide bond formation.^44,45^ LPS biosynthesis includes many enzymes, including the gene product of *waaD* (also referred to as *rfaD*). It has been shown previously that antibodies targeting the outer membrane surface can exhibit an antimicrobial effect in a Δ*waaD* strain that is not observed in the wildtype.^46,47^ With a panel of Bicycle molecules, growth inhibition was not observed in any of the strains tested, nor was inhibition observed with treatment of a cocktail of Bicycle molecules from each competition bin (Table S1). Bicycle molecule binding modes were further investigated using structural approaches to identify specific protein interaction surfaces.

### Structural Analysis with Cryogenic Electron Microscopy

A representative Bicycle molecule from each competition bin was chosen for structural determination in complex with SfLptDE_FL_ by cryo-EM. A total of ∼10,000 movies were collected over 24 h for the first data set (Compound **12**, Figure 3B, PDB 9I97), yielding ∼1.2 million particles after preliminary particle extraction. Processing of the data set generated a reconstruction at 2.48 Å resolution, built from ∼330,000 particles. The achieved resolution allowed to resolve both the β-barrel of SfLptDE and the Bicycle molecule (Figures 3A and S4), showing binding of the Bicycle molecule along the outer rim of the β-barrel on the extracellular side of the protein between β-sheets 11–13. While the Bicycle molecule made specific peptide contacts with the protein, a significant portion of the molecule was embedded within the detergent micelle and did not appear to cause any significant structural rearrangement of the barrel (Figure S5).

**Figure 3:**
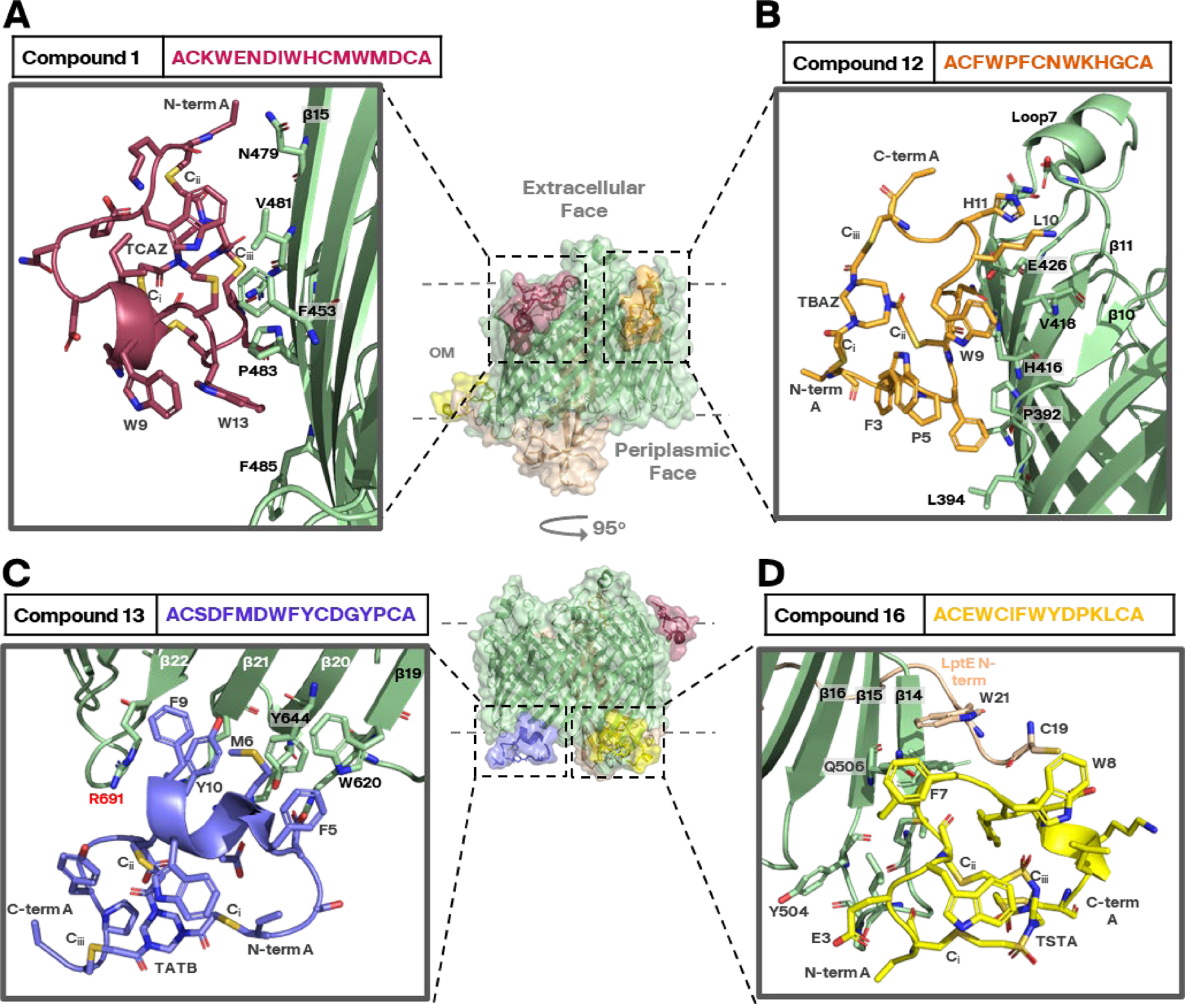
Bicycle molecules binding to the β-barrel SfLptDE complex. Cryo-EM data processing showed four Bicycle molecules (**1**, **12**, **13,** and **16**, PDB IDs 9I92, 9I97, 9I98, and 9Q8N respectively) binding to four independent epitopes across the protein, either to the extracellular (A, B) or periplasmic (C, D) “rim” of SfLptD. All Bicycle molecules had a protein binding face and a detergent binding face, with protein contacts being stabilized mostly via hydrophobic interactions. Charged residues on the Bicycle molecules were typically found facing away from the protein. Overall, the backbone of SfLptDE showed little rearrangement upon Bicycle molecule binding compared to the apo conformation (Figure S5). R691 is highlighted in red in (C) as this is the site of one of the 3 sequence variants between *E. coli* LptD and SfLptD (Figure S1A). The composite SfLptDE overlay image in the center is for illustration only; in each structure, SfLptDE was bound to one Bicycle molecule only. Electron densities for all Bicycle peptide binders can be found in Figure S6.

Extracted particles from this data set were then split equally into subsets of particles based on the number of hours taken for data collection and subsets corresponding to one, two- and three hours worth of collection were processed. After analyzing the minimum number of particles required to achieve sub-3 Å resolution in the final 3D reconstruction, all subsequent cryo-EM collections were reduced to 6–8 h (with the same sample preparation conditions as established), yielding an average of ∼3500 movies per data set. Processing of each of these collections resulted in final reconstructions with a resolution between 2.3 – 2.9 Å (Figure S4, see Table S2 for further details on each data set), meaning that 3 collections per 24 h could be performed without compromising on overall resolution (Figure S4D).

In all data sets, Bicycle molecules could be built into the generated electron density map, revealing binding sites at four separate epitopes across the protein complex. All Bicycle molecules across each epitope had a similar binding mode, as described for compound **12,** i.e., with a protein binding face interacting with the rim of the β-barrel, and a detergent binding face (Figure 3). Overall, the data indicated two binding positions at the periplasmic face of the protein contacting β-sheets 15–16 (compound **16**, PDB 9Q8N Figure 3D) and β-sheets 20–21 (compound **13**, PDB 9I98 Figure 3C) and two binding positions at the extracellular face of the protein contacting β-sheets 11–13 (compound **12**, PDB 9I97 Figure 3B) and β-sheets 14–16 (compound **1**, PDB 9I92 Figure 3A) with additional contacts at extracellular facing loop 7. In all reconstructions, the N-terminal β-jellyroll domain was significantly less resolved than the membrane embedded β-barrel domain, although it could be resolved to some degree with focused reclassification and additional data collection. However, given that the Bicycle molecules were not targeting this region, this additional classification step was typically not performed (Figure S4E).

One Bicycle molecule, Compound **16**, binding at the periplasmic face of the SfLptD β-barrel at β-sheets 15 – 17 is situated near the N-terminal region of LptE, close to where LptE anchors into the membrane via its lipoprotein tail. The reconstruction of this Bicycle-molecule bound state shows additional density consistent with the lipid tails extending from the N-terminal cysteine of the molecule (Figure S8). Since our other ligand bound structures or previously reported structures of the LptDE complex have not shown density for the lipoprotein tail, we speculate that the presence of the Bicycle molecule stabilized this region of LptE.

The binding site of Compound **13** on SfLptD (near the periplasmic side of β-sheets 20–22) contains residue Arg691 (highlighted in red in Figure 3C). As shown in Figure S1, the corresponding residue in *E. coli* LptD is a tryptophan, and it is therefore considered unlikely that this molecule would be able to bind to *E. coli* LptDE, due to the hydrogen bonding stabilization between the hydroxyl group of Asp12 on Compound **13** and the guanidinium group of Arg691 on SfLptD (Figures S1 and S7).

To confirm the SPR competition binning, four additional cryo-EM data sets using different Bicycle molecules were collected from competition bin 1, which had the most unique sequences. The resulting models showed that all four Bicycle molecules bind in the same region and with very similar binding mode as Compound **1** (Figure 4A), indicating that the SPR-based competition binning can effectively be used to sort molecules according to different binding locations. All five imaged molecules (Compounds **1–5**) in this epitope had an aligned sequence contacting the β-barrel of SfLptD, with a ubiquitous W×(D/EE) motif making a consistent interaction (Figure 4B). In each of these interactions, the Bicycle molecule tryptophan indicated was docked into a hydrophobic pocket formed by Pro483, Phe485, Tyr516, and Pro518 on LptD, with further stabilization coming from hydrogen bonds formed by the guanidinium group of Arg520 and the hydroxyl group of Tyr516 contacting both the amino acid backbone and the side chain functional groups of the D/EE residues (Figure S7). The remaining residues of the five Bicycle molecules aligned poorly, especially in the regions facing the detergent micelle (Figure 4 and Table 1). Mutation of the key residues in the W×D/EE motif resulted in significant aberration in peptide binding to SfLptDE; substitution of the tryptophan residue with an alanine abolishes binding in SPR while substituting the aspartic/glutamic acid residues with an alanine significantly weakens peptide affinity to LptDE (Figure 4 and Table S3). Overall, this indicated a level of motif specificity in Bicycle molecules binding to SfLptDE, despite the relatively small interaction surfaces, demonstrating this high-throughput screening process did not select for merely nonspecific detergent-binding molecules. However, mutation of the hydrophobic residues in the peptide loop facing the detergent micelle also abolished binding for Compound **1** (Figure 4 and Table S3), indicating that the micelle plays a significant role in the stabilization of Bicycle peptide binding to SfLptDE.

**Figure 4:**
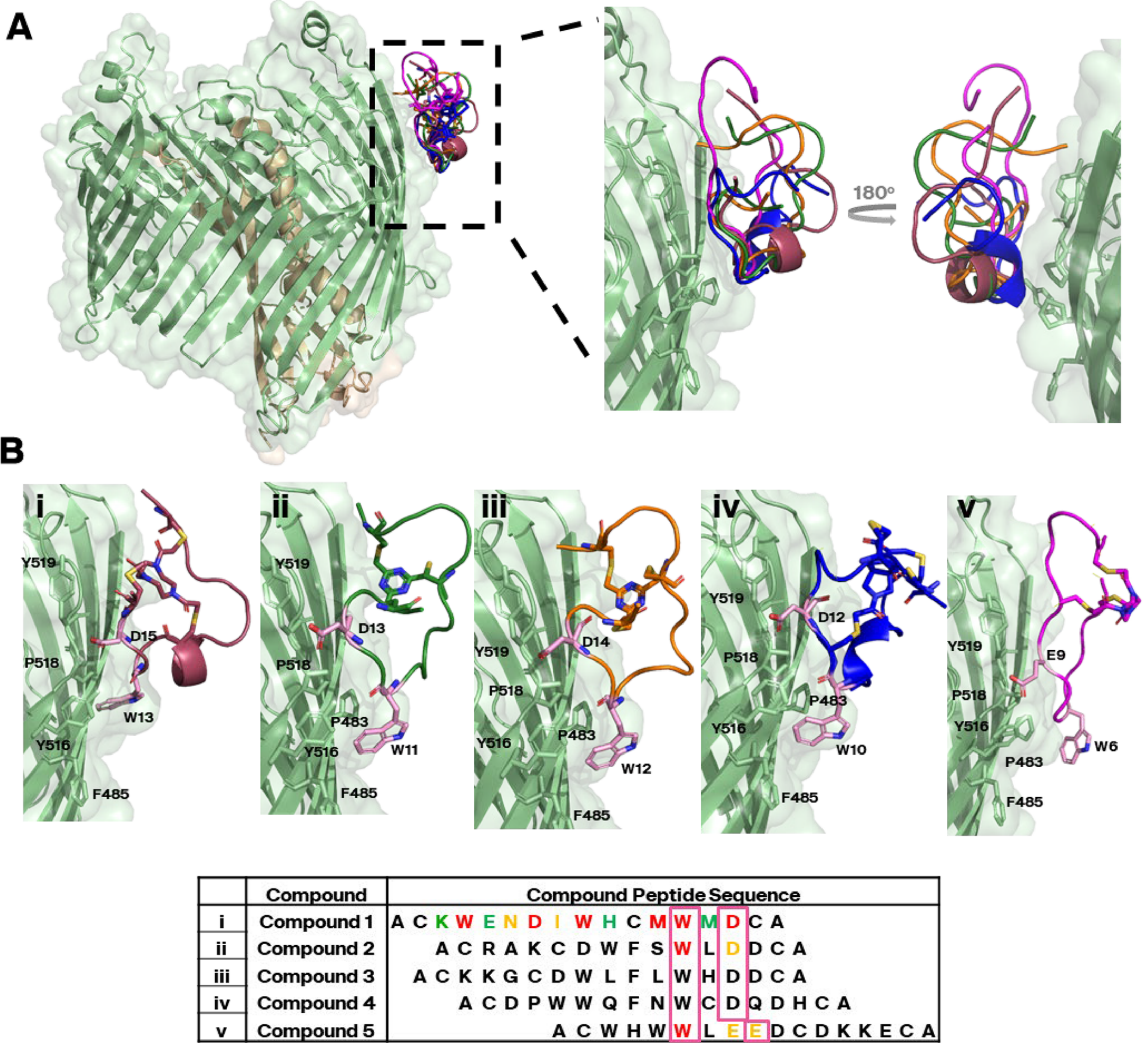
Bicycle binders in the same competition bin (competition bin 1) were confirmed to bind to the same location (epitope 1) on SfLptDE. (A) Overlay image of five separate Bicycle molecules (**1–5,** PDB IDs 9I92, 9I93, 9I94, 9I95, and 9I96 respectively) belonging to competition bin 1 bound to the same location (epitope 1) on SfLptDE at the extracellular-facing rim of the β-barrel and between β-sheets 11–13. Bicycle molecule scaffolds have been removed from this panel for clarity. (B i–v) All five molecules contacted the protein with a conserved W×(D/EE) motif (pink boxes). These interactions were stabilized primarily by hydrophobic side chains and limited hydrogen bonding from the LptD barrel (Figure S7), but the rest of the molecule is poorly aligned, indicating motif specificity. Outside of the LptDE-contacting motif, sequence diversity was high. Compounds **1**, **2,** and **5** were subject to an alanine-substitution experiment – residues highlighted in green could be substituted with an alanine with little to no effect on binding affinity to SfLptDE_FL_, residues in yellow resulted in a marked decrease in affinity and residues labeled in red were not tolerant to substitution, completely losing any binding to SfLptDE. Residues in black were not tested in the alanine substitution experiment. Binding affinity values for each peptide are given in Table S3.

## Conclusions

LptDE is a target of significant interest in the field of antimicrobial drug discovery.^25,27–29^ The goal of this work was to identify LptDE-binding molecules that could be used to develop novel inhibitors of SfLptDE that block LPS transport without the need for cellular permeation. In this study, a workflow was demonstrated to identify and characterize Bicycle molecules interacting with different epitopes of SfLptDE.

Drug discovery against LptDE is made challenging by the lack of molecules with LptDE-targeting activity and the absence of appropriate functional assays to test for LptDE inhibition. At the hit validation stage, molecules may have such weak activity that their effect on cell growth cannot be measured, even for essential targets such as LptDE. Chemical genomic approaches such as knockouts of associated proteins can be useful to sensitize the cell to antimicrobial compounds but even the panel of strains with weakened OMs tested here showed no susceptibility to the Bicycle molecules shown to bind.

Screening of membrane associated targets presents several challenges in drug discovery. One of the major challenges is presentation of the target protein in a relevant conformation. This has been addressed in recent years through presenting the target in a detergent environment.^48^ The presence of detergent introduces further challenges, in particular the ability to unambiguously and efficiently distinguish hits which bind solely to the target protein from those which interact with the detergent. A robust protein purification protocol together with faster data collection and processing was crucial in enabling us to characterize multiple molecules binding multiple binding epitopes.

The only well-validated LptDE inhibitor, Murepavadin has not been structurally characterized in complex with LptDE, but resistant mutations point to it binding at the N-terminal domain of *Pseudomonas* LptDE.^27^ The N-terminal domain is highly divergent between *Pseudomonas* and *Shigella* LptDE. Comparison between the *Shigella* and *Pseudomonas* structures shows the site of a Murepavadin resistance mutation is distant from the 4 binding sites identified in this study (Figure S10A). Whether the molecules identified here are able to bind to Pseudomonas LptDE is out of scope for this study, although some conserved residues are present between the Shigella and Pseudomonas protein at the same epitope regions identified for the Shigella binders (Figure S10). It is unclear to us why our method identified only detergent interface binders and not peptides binding to locations which cause functional inhibition. First, it is conceivable that the lack of known natural inhibitors for *Enterobacterales* LptDE (i.e., *E. coli* and *Shigella flexneri*) is due to the much lower “tractability” to inhibitors of the protein in the absence of the Pseudomonas-specific domain. Second, the large interaction face provided by the DDM to the “back” face (i.e., away from the protein) of the Bicycle molecule leads to higher affinities. Modification of the protein formulation may remove this DDM-driven bias. Alternative formulations include: screening whole cells overexpressing LptDE;^28,47^ LptDE expressed in outer membrane vesicles;^49^ liposomes;^50^ nanodiscs;^45,51^ or SMALPS.^52^ Each of these methods brings additional challenges for purification and ease of screening and further work is needed to explore their potential for use in our pipeline.^53,54^

Here, cryo-EM was deployed in an early stage discovery capacity, showing that it can be effectively used to probe the potential ligand chemical space across an entire target molecule. Structures for 8 molecules binding LptDE at 4 epitopes are presented, all of which interact to some degree with the detergent micelle. Importantly, the resolution achieved allowed clear identification of hit molecules which interacted with the detergent and has acted as an efficient, early stage triage. Given the high level of current interest in outer membrane targets from both prokaryotic and eukaryotic organisms, we believe this work represents a widely applicable technique which will assist drug discovery scientists in the early identification of detergent binders and thereby deselecting them from further work.

## Experimental Methods

### SfLptDE Protein Expression and Purification

SfLptDE_FL_ in pBAD22 was received from the Huang lab.^16^ A truncation in SfLptD (deletion of residues 26–201, (Figure S1) was made by Q5 mutagenesis according to the manufacturer’s protocol (New England Biolabs) using the pBAD22 plasmid encoding full-length SfLptDE, with a hexahistidine tag on the C-terminus of LptE. The truncated SfLptD protein was coexpressed with an LptE which has a tobacco etch virus (TEV) cleavage site before the hexahistidine tag (Figure S1). Plasmids were transformed into electrocompetent BL21 (DE3) Δ*cyo* cells with a deletion of cyoB, and truncation of cyoA and cyoC, removing the major contaminant CyoABCD in the purification process and reducing the steps required to generate a clean sample.^55^ Cells were grown in LB supplemented with 100 μg/mL ampicillin at 37 °C 180 rpm until they reached an OD_600_ of 0.6–1.0 and induced with 0.1% (w/v) arabinose. Cells expressing full-length SfLptDE were induced at 37 °C and 180 rpm for a further 2.5 h whereas truncated SfLptDE expressing cells were induced at 18 °C, 150 rpm for 20 h.

Cells were harvested by centrifugation (4 °C, 20 min, 4200 rpm, Beckman J6-HC, JS 4.2). Cell pellets were resuspended in TBS (20 mM Tris-HCL (pH8), 300 mM NaCl) supplemented with DNaseI and manually homogenized with a dounce. Cells were lysed via two passes at 20 kpsi using a cell disruptor (Constant Systems, 0.75 kW model), and centrifuged at 42,000 rpm (45Ti rotor; Beckman Optima XE-90) for 50 min at 4 °C. The supernatant was discarded, and the total membrane pellet was resuspended in 2% (w/v) LDAO buffer (TBS) with a dounce homogenizer and stirred for 1 h at 4 °C. The suspension was centrifuged at 30,000 rpm (45Ti rotor; Beckman Optima XE-90) for 30 min at 4 °C, and the supernatant loaded onto a 5 mL IMAC column of Ni-charged chelating Sepharose (Cytiva) equilibrated in buffer A (300 mM NaCl, 20 mM Tris-HCL (pH 8), 30 mM imidazole, 0.15% (w/v) DDM). The column was then washed with 30 column volumes (CV) of buffer A and the protein eluted with 3 CV buffer B (300 mM NaCl, 20 mM Tris-HCl (pH 8), 200 mM imidazole, 0.15% (w/v) DDM). The eluted protein was concentrated with an Amicon Ultra centrifugal filter (100 kDa molecular weight cut off) and applied to a size exclusion column (Superdex 200 Increase 10/300 GL) equilibrated in 10 mM HEPES, 100 mM NaCl, 0.05% (w/v) DDM pH7.5. SfLptDE complex peak fractions were pooled, concentrated and flash frozen in liquid nitrogen.

Purified SfLptDE was chemically biotinylated by incubating a amine-reactive biotin (using EZ-Link NHS-LC-LC-Biotin 21343, Thermo Scientific) with purified SfLptDE in a 1:5 protein:biotin molar ratio in 10 mM HEPES-NaCl (pH 7.5), 100 mM NaCl, 0.05% (w/v) DDM overnight at 4 °C before quenching with the addition of 1 M Tris-HCl (pH 8). Excess unreacted biotin was removed by size exclusion chromatography (Superdex 200 Increase 10/300 GL pre-equilibrated with 10 mM HEPES, 100 mM NaCl, 0.05% DDM). Mass Spectrometry analysis of samples confirmed the presence and modification status of the complex (Figure S9).

### MICs

Minimum inhibitory concentrations were measured following CLSI guidelines.^56^ Briefly, strains were spread onto Luria Broth Agar or Nutrient Agar plates and grow overnight. For *E. coli* strains, Δ*surA*, Δ*bamB,*, Δ*bamC*, Δ*bamE*, Δ*lptM*, Δ*waaD* bacteria from the plate were suspended in 0.9% saline to produce a bacterial density corresponding to a 0.5 McFarland standard then diluted 1 in 400 into cation-adjusted Mueller-Hinton broth (CaMHB, product code 90922, Millipore) and 200 μL per well was added to a plate containing a small volume of compound in DMSO.

For the hyperporinated strain GKCW102 (and nonporinated control GKCW101), the bacteria were prepared as previously described in CaMHB medium.^57^ The compounds were dispensed from a 10 mM stock in DMSO using a D300e dispenser (Tecan) to a top concentration of 128 μg/mL. Plates were then incubated overnight for 18–22 h at 37 °C. At least two biological replicates were carried out for each result reported.

### Selection of SfLptDE_FL_/ SfLptDE_T_ Protein Specific Bicycle Molecules by Phage Display

Bicycle bacteriophage (phage) libraries containing linear peptides (between 11 and 17 amino acids) with 3 cysteines cyclized on phage to a central scaffold to generate bicyclic peptide libraries. The libraries were used in selections against full length and truncated SfLptDE. Biotinylated protein immobilized to streptavidin beads was used to affinity pulldown phage particles over four subsequent rounds of selection with decreasing target protein concentration. After Round 4, phage clones were sequenced and then certain individual clones were assessed in their ability to bind SfLptDE using an Alphascreen and ELISA assay. Select binders were made individually by solid phase peptide synthesis for further characterization.

### Binding and Affinity Characterization Using Surface Plasmon Resonance (SPR)

SPR was carried out using a Biacore T200 or 8K+ instrument (Cytiva) at 25 °C, using streptavidin (SA) sensor chips (Cytiva) in a premade buffer of 1× HBS-N, pH 7.4 (Cytiva) supplemented with 0.05% (w/v) DDM and 1% (v/v) DMSO. 200 nM of randomly biotinylated SfLptDE was immobilized onto the SA chip, aiming for a ligand RU of 1000 RU per chip surface. Following 5–10 injections of buffer only, Bicycle molecules were injected at 5 or 8 different concentrations (highest concentration typically 5 μM) using a 60 s association and 650 s dissociation time, alongside buffer-only blanks. Binding constants between the immobilized protein and Bicycle peptides were tested using single- or multicycle kinetics. Following solvent correction (to correct for DMSO bulk effects) and double-reference correction using a reference flow cell and blank buffer injections, Biacore Insight Evaluation software (Cytiva) was used to fit 1:1 binding model to the data to calculate the kinetically derived binding affinities.

### Competition Binning by SPR

Competition binning experiments were conducted under similar conditions to the binding affinity experiments above, except for an increased target-chip occupancy (3000 RU) to maximize the signal-to-noise ratio of the response. Using a Biacore 8K+ instrument (Cytiva), pairs of Bicycle molecules were flowed over the chip surface in an ″A–B–A” format injection, where Molecule A (at 5 μM) was injected over the target-bound surface before the injection of Molecule B (at 5 μM) to identify whether the presence of Molecule A reduced the response of Molecule B. Molecule B was also injected following an injection of buffer “buffer-only control”. To increase throughput, the concentration of peptide for all injections was 5 μM. The change in response (*R*_B_) was measured, where *R*_B_ = [response level during injection of Molecule B] – [response level before injection of Molecule B] (Figure S3C). As the injections occurred over 8 flow cells, *R*_*B*_ was first normalized to the ligand density of the specific flow cell.

If the *R*_B_ measured after injection of Molecule A was <80% of *R*_B_ after injection of the buffer-only control, the interaction was scored as “competitive”, otherwise it was scored as “not competitive”. For each pair of molecules, three runs were conducted with each individual molecule tested as both Molecule A and Molecule B (i.e., two observations were taken per pair of peptides per experiment), for a maximum of 6 independent replicates. Number of replicates used for each interaction in shown in Figure S3E. In some cases, less than 6 replicates were used, this was either because an interaction was not tested in one of the runs or because an interaction was excluded because the *R*_B_ of the interaction was negative due to interference on the chip surface during the injection. Each compound was tested a maximum of 3 times against itself (i.e., when the sample compound was both Molecule A and Molecule B). The low (<80%) threshold for competition biases the method toward finding interactions. Compound **12** could not be shown to compete with itself and interactions with compound **12** were excluded from the results (Figure 2C). A blank (buffer injected as Molecule A and B) was used before each sample and a blank-correction applied.

The scores were formatted as a heatmap in Excel (Microsoft), then clustering was carried out manually using the heatmap to sort groups of molecules with similar competition patterns. Clusters of molecules with similar activity were designated as belonging to the same competition bin. During the clustering analysis, the peptide sequence of the Bicycle molecules was blinded.

### Cryo-EM Sample Preparation

For all samples, 80 μM SfLptDE was incubated with Bicycle molecules in a 1:3 molar ratio. Samples were centrifuged at 15,000 *g* on a benchtop centrifuge and the sample was subsequently stored on ice. Three μL of sample was applied to Quantifoil R1.2/1.3 Cu 300 mesh grids that had been glow discharged on both sides for 30 s at 20 mA using a PELCO easiGlow (Ted Pella, Inc.) glow discharge cleaning system. Samples were applied onto grids at 4 °C and 95% relative humidity using a Vitrobot Mark IV (Thermo Scientific), blotted for 2 s with a blot force of 0, then plunge frozen in liquid ethane.

### CryoEM Data Acquisition

Electron micrographs were captured at Cryo-EM Facility, Department of Biochemistry, University of Cambridge on a Thermo Scientific Titan Krios TEM equipped with a Falcon 4i direct electron detector and SelectrisX energy filter with 10 eV slits, using EPU 2 acquisition software. All collections were carried out at a pixel size of 0.729 Å per pixel, with an average total electron dose of ∼50.97 e^–^/Å^2^ used over 50 dose-fractioned movie frames and a total exposure time of 4.39 s. The defocus targets ranged from −0.6 to −1.6 μm. A total of 3000–7000 movies were collected per sample, averaging at ∼6 h per data set. See (Table S2) for detailed information on each data collection.

### CryoEM Processing, Model Building, and Refinement

For each data set, preprocessing (motion correction/CTF estimation) were estimated in WARP, concurrent with the data collection, with micrographs with an estimated CTF above 4 Å being discarded. Particles were picked using WARP (Version: 1.0.9) and extracted with a box size of 352 pix., then imported to cryoSPARC (Version: 4.1.1)^58^ for further processing. An initial round of 2D classification was performed, and particles from classes representing SfLptDE were used to generate three *ab initio* volumes. Additionally, particles from classes excluded at the initial 2D classification stage were also used to generate 3× *ab initio* volumes. All picked particles and all 6× *ab initio* volumes were then used as an input for heterogeneous refinement. Particles and volumes from the best classes were then used as inputs for nonuniform refinement. Final resolutions of the generated reconstructions maps ranged between 2.3 – 2.9 Å (see Table S2 for specific data set parameters).

Using PDB-ID 4Q35 ^16^ as a template for the SfLptDE β-barrel, rigid body fitting into the generated reconstruction was carried out in ChimeraX (version 1.8),^59^ followed by manually rebuilding and refitting in WinCoot (version 0.9.8.92).^60^ Bicycle molecules were built de novo in WinCoot. Real-space refinement was carried out in Phenix (Version 1.20.1–4487),^61^ applying Ramachandran restraints where appropriate.^62^ See Table S2 and Figure S4 for further details.

### Protein Mass Spectrometry

SfLptDE_FL_ was analyzed by LCMS using a Waters BioAccord time-of-flight mass spectrometer. Approximately 1 μg of total protein was injected via a Waters Acquity Premier liquid chromatography system. Components were fractionated using an 8 min method with a 5.5 min linear water/acetonitrile containing 0.1% (v/v) formic acid. The LC column used was a Waters Acquity BEH, C4, 50 × 2.1 mm, 1.7 μm, 300 Å, flow rate = 0.4 mL/min at a column temperature of 80 °C. UV absorbance at 280 nm was measured prior to elution into the mass spectrometer which was operated in positive in mode with the cone set to 30 V and the *m*/*z* range = 400–7000. Solvents were Fisher Scientific Ultrapure mass spectrometry grade.

### Peptide Synthesis

All peptides were synthesized on Rink amide resin using standard Fmoc (9-fluorenylmethyloxycarbonyl) solid phase peptide synthesis using 2 automated systems. Peptide synthesis at 25 μmol was run on a Biotage SyroII automated synthesizer. Peptide synthesis (80–240 μmol) was carried out with a Gyros Symphony X automated synthesizer. Following cleavage from the resin using a cocktail of 95% TFA, 2.5% triisopropylsilane, 2.5% H_2_O with 25 mg dithiothreitol (DTT) per ml, peptides were precipitated with diethyl ether and dissolved in 50:50 acetonitrile/water. Peptides for bicyclization were diluted to 2 mM in 50:50 acetonitrile:water, 2.6 mM scaffold solution and 200 mM ammonium bicarbonate to give final concentrations of 1, 1.3 and 100 mM respectively. Completion of cyclization was determined by matrix assisted laser desorption ionization time-of-flight (MALDI-TOF) or LC-MS. Once complete, the cyclization reaction was quenched using *N*-acetyl cysteine (10 eq 1 M solution over peptide) and lyophilized.

### Peptide Purification

Crude peptides, following lyophilization, were dissolved in an appropriate solvent system and filtered through a 0.45 μm PES filter before loading on to a 5 μm, 100 Å, 21.2 × 100 mm Kinetex XB-C18 column (Phenomenex). Prep HPLC gradients using 0.1% TFA in H2O (solvent A) and 0.1% TFA in acetonitrile (solvent B) were selected based on retention time of samples analyzed either after cleavage, or during cyclization on a 2.6 μm, 100 Å, 2.1 × 50 mm Kinetex XB-C18 analytical column on a gradient of 10–80% over 3 min in 0.1% TFA in acetonitrile.

### Compound Quality Control (QC)

Peptide fractions of sufficient purity and correct molecular weight, verified by MALDI-TOF and HPLC or LC-MS, were pooled and lyophilized. Compound purity was determined by UV absorbance at 220 nm using an LC gradient 5–95% solvent B, where solvent A is 0.1% TFA in H_2_O and solvent B is 0.1% TFA in acetonitrile over 9 min on a 2.6 μm, 100 Å, 2.1 × 50 mm Kinetex XB-C18 analytical column. All compounds, except compound 10 (88.5%), were >95% pure by HPLC analysis (Table S4). Solution concentrations prior to QC were determined by UV absorption (UV–vis) using the extinction coefficient at 280 nm, which was based on Trp/Tyr content.

This study included no animal or human studies.

## Supplementary Material





## Notes

MIC activity of the compounds described in the paper; cryogenic electron microscopy table of collection parameters; tolerances for amino acid substitutions at motif identified for epitope 1 peptides; comparison of the sequences of *Shigella flexneri* and *Escherichia coli* LptDE; overview of the phage selection process; SPR binding and Competition assays; details of the cryogenic electron microscopy collection and processing workflow; alignments of structures of LptDE bound by Bicyclic peptide binders; electron density closeups of each Bicycle peptide binder analyzed by cryo-EM; contacts made between bicycle peptide binders and SfLptDE; observations of the LptE lipid tail in mass spectrometry and cryo-EM; mass spectrometry of the SfLptDE complex; alignment of *Pseudomonas aeruginosa* LptDE with SfLptDE and bicycle peptide binders; summary of peptide HPLC data; and scaffold structures (PDF)
Molecular formula strings (CSV)

## Abbreviations

BAM: β-barrel assembly machinery
CaMHB: cation-adjusted mueller-hinton broth
DDM: *n*-dodecyl-β-d-maltoside
ELISA: enzyme-linked immunosorbent assay
EM: electron microscopy
GPCR: G-protein coupled receptor
HBS-N: HEPES buffered saline
*K*_d_: dissociation constant
LPS: lipopolysaccharide
Lpt: lipopolysaccharide transport
MICs: Minimum inhibitory concentrations
OM: outer membrane
PDB: protein data bank
PES: Poly­(ether sulfone)
p*K*_d_: –log10­(*K*_d_)
QC: quality control
RU: response units
SA: streptavidin
Sf: *Shigella flexneri*
SfLptDE_FL_: full-length construct of SfLptDE
SfLptDE_T_: truncated construct of SfLptDE
SPPS: solid phase peptide synthesis
SPR: surface plasmon resonance
TEV: tobacco etch virus
UV: ultraviolet
