# Supplementary material for: High-Throughput Identification and Characterization of LptDE-Binding Bicycle Peptides Using Phage Display and Cryo-EM
Source: J Med Chem. 2025 Oct 6;68(20):21144–55. doi: 10.1021/acs.jmedchem.5c00307 (PMC12557396; doi:10.1021/acs.jmedchem.5c00307)
Supplement: Supplementary file 1 [file jm5c00307_si_001.pdf]

Supporting Information

High Throughput Identification and Characterization of LptDE-binding Bicycle® Peptides Using Phage Display and Cryo-EM

Shenaz Allyjaun*[1,2], Emily Dunbar*[1], Steven W. Hardwick[3], Sarah Newell[2], Finn Holding[2], Catherine E Rowland[2], Megan A. St. Denis[2], Simone Pellegrino[2], Gustavo Arruda Bezerra[2], Nikolaos Bournakas[2], Dimitri Y. Chirgadze[3#], Lee Cooper[3], Giulia Paris[3], Nick Lewis[2], Peter Brown[2], Michael J. Skynner[2], Michael J Dawson[2], Paul Beswick[2], Julia Hubbard[1#], Bert van den Berg[1#], Hector Newman[2#]

*These authors contributed equally to this work

#Corresponding author

Hector Newman: hector.newman@bicycletx.com

Julia Hubbard: Julia.Hubbard@newcastle.ac.uk

Bert Van Den Berg: Bert.Van-Den-Berg@newcastle.ac.uk

Dima Chirgadze: dyc21@cam.ac.uk

1. Biosciences Institute, Faculty of Medical Sciences, Newcastle University, Newcastle upon Tyne, NE2 4HH, UK

2. BicycleTx Ltd, Blocks A&B, Portway Building, Granta Park, Great Abington, Cambridge, CB21 6GS, UK

3. Department of Biochemistry, University of Cambridge, 80 Tennis Court Road, Cambridge, CB2 1GA, UK

Contents

Table S1: MIC activity of the compounds described in the paper.

Table S2: Cryogenic electron microscopy table of collection parameters

Table S3: Tolerances for amino acid substitutions at motif identified for epitope 1 peptides

Figure S1: Comparison of the sequences of Shigella flexneri and Escherichia coli LptDE

Figure S2: Overview of the phage selection process

Figure S3: SPR binding and Competition assays.

Figure S4: Details of the cryogenic electron microscopy collection and processing workflow.

Figure S5: Alignments of structures of LptDE bound by Bicyclic peptide binders

Figure S6: Electron Density closeups of each Bicycle peptide binder analysed by cryo-EM.

Figure S7: Contacts made between Bicycle peptide binders and SfLptDE

Figure S8: Observations of the LptE lipid tail in mass spectrometry and cryo-EM

Figure S9: Mass spectrometry of the SfLptDE complex

Figure S10: Alignment of *Pseudomonas aeruginosa* LptDE with SfLptDE and Bicycle peptide binders

Table S4: Summary of peptide HPLC data

Table S5: Scaffold structures

Table S1. MIC activity of each Bicycle molecule binder against a panel of wild-type and outer-membrane disrupted *E. coli* strains

Epitope	Compound	Affinity Kd (nM)	*ΔbamB*	*ΔbamC*	*ΔbamE*	*ΔsurA*	*ΔlptM*	*ΔwaaD*
**1**	1	13	>128	>128	>128	>128	>128	>128
	2	26	>128	>128	>128	>128	>128	>128
	3	133	>128	>128	>128	>128	>128	>128
	4	22	>128	>128	>128	>128	>128	>128
	5	287	>128	>128	>128	>128	>128	>128
**2**	12	61	>128	>128	>128	>128	>128	>128
**3**	13	74	>128	>128	>128	>128	>128	>128
**4**	16	36	>128	>128	>128	>128	>128	>128
**Combination**	1, 12, 13, 16	-	>128	>128	>128	>128	>128 (n=1)	>128

Table S1. MIC values of the Bicycle peptides in 9 *E. coli* strains in Cation Adjusted MHB medium. *E. coli* mutants with attenuated outer membranes via knockouts of the OM protein biosynthesis pathway (*ΔbamB, ΔbamC, ΔbamE, ΔsurA)*[1], weakened LptDE complex (*ΔlptM*[1]), attenuated LPS production (*ΔwaaD*)[1]. Values are representative of at least 2 biological replicates unless otherwise stated. Affinity measurements by SPR (see Table 1 in the main text).

Table S2: Cryo-EM parameters

$SfLptDE_{FL}$ in complex with Compound:	1	2	3	4	5	12	13	16
Microscope	FEI Titan Krios							
Detector	F4i counting							
Voltage (keV)	300							
Nominal Magnification	165,000x							
Pixel Size, Å per pixel	0.729							
Exposure, sec	4.39							
Dose electrons/pixel/sec (measured)	6.42							
Dose electrons/$Å^2$/sec (calculated)	12.06							
Total Dose, electrons/$Å^2$ (measured)	53.03							
Number of Fraction (processing Frames)	50							
Defocus Range (µm)	(-1.8) – (-0.6)							
# Micrographs used for processing	6,347	2,400	2,500	7,533	4,561	10,357	3,000	3,000
Initial Particles (before 2D classification cleaning)	893,552	135,693	308,425	944,127	447,693	1,289,942	395,973	444,572
# Particles in Final map	158,113	101,063	116,397	175,457	129,785	512,272	151,742	168,619
Map resolution (Å)	2.41	2.86	2.84	2.35	2.67	2.48	2.68	2.54
Accession Codes (PDB/EMDB)	PDB 9I92 / EMD-52749	PDB 9I93 / EMD-52750	PDB 9I94 / EMD-52751	PDB 9I95 / EMD-52752	PDB 9I96 / EMD-52753	PDB 9I97 / EMD-52754	PDB 9I98 / EMD-52755	PDB 9Q8N / EMD-52896

Table S3: Tolerances of amino acid substitutions at the SfLptD β-barrel contacting motif for epitope 1 Bicyclic peptide binders

Compound	Peptide Sequence	Affinity to FL SfLptDE (Kd, nM)*
**1**	A C K W E N D I W H C M W M D C A	7.6 ± 0.15 (3)
**1a**	A C *A* W E N D I W H C M W M D C A	8.1 ± 0.33 (2)
**1b**	A C K *A* E N D I W H C M W M D C A	NB
**1c**	A C K W *A* N D I W H C M W M D C A	7.9 ± 0.36 (3)
**1d**	A C K W E *A* D I W H C M W M D C A	NB
**1e**	A C K W E N *A* I W H C M W M D C A	NB
**1f**	A C K W E N D *A* W H C M W M D C A	6.2 ± 0.20 (2)
**1g**	A C K W E N D I *A* H C M W M D C A	NB
**1h**	A C K W E N D I W *A* C M W M D C A	8.1 ± 0.17 (2)
**1i**	A C K W E N D I W H C *A* W M D C A	NB
**1j**	A C K W E N D I W H C M *A* M D C A	NB
**1k**	A C K W E N D I W H C M W *A* D C A	7.5 ± 0.35 (2)
**1l**	A C K W E N D I W H C M W M *A* C A	NB
**2**	A C R A K C D W F S W L D D C A	7.9 ± 0.19 (3)
**2a**	A C R A K C D W F S *A* L D D C A	NB
**2b**	A C R A K C D W F S W L *A* D C A	6.6 ± 0.18 (2)
**5**	A C W H W W L E E D C D K K E C A	6.5 ± 0.06 (3)
**5a**	A C W H W *A* L E E D C D K K E C A	NB
**5b**	A C W H W W L *A* E D C D K K E C A	6.3 ± 0.25 (2)
**5c**	A C W H W W L E *A* D C D K K E C A	6.5 ± 0.29 (2)

*NB denotes a non-binding peptide.

A

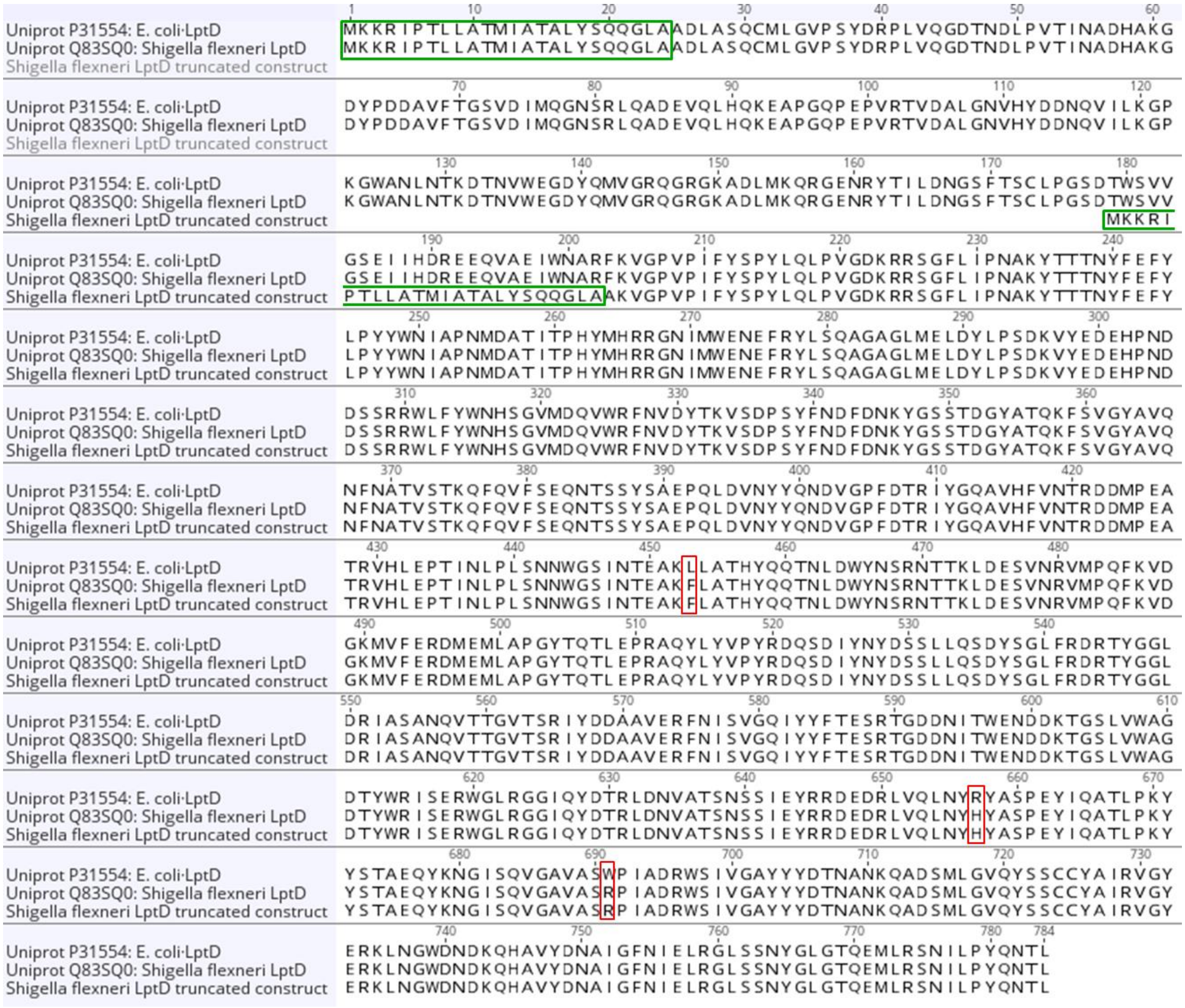
Uniprot P31554: E. coli·LptD
Uniprot Q83SQ0: Shigella flexneri LptD
Shigella flexneri LptD truncated construct
MKKRIPTLLATMIATALYSQQGLAADLASQCMLGVPSYDRPLVQGDTNDLPVTINADHAKG
MKKRIPTLLATMIATALYSQQGLAADLASQCMLGVPSYDRPLVQGDTNDLPVTINADHAKG
DYPDDAVFTGSVDIMQGNSRLQADEVQLHQKEAPGQPEPVRTVDALGNVHYDDNQVILKGP
DYPDDAVFTGSVDIMQGNSRLQADEVQLHQKEAPGQPEPVRTVDALGNVHYDDNQVILKGP
KGWANLNTKDTNVWEGDYQMVGRQGRGKADLMKQRGENRYTILDNGSFTSCLPGSDTWSVV
KGWANLNTKDTNVWEGDYQMVGRQGRGKADLMKQRGENRYTILDNGSFTSCLPGSDTWSVV
MKKRI
GSEIIHDREEQVAEIWNARFKVGPVPIFYSPYLQLPVGDKRRSGFLIPNAKYTTTNYFEFY
GSEIIHDREEQVAEIWNARFKVGPVPIFYSPYLQLPVGDKRRSGFLIPNAKYTTTNYFEFY
PTLLATMIATALYSQQGLAAKVGPVPIFYSPYLQLPVGDKRRSGFLIPNAKYTTTNYFEFY
LPYYWNIAPNMDATITPHYMHRRGNIMWENEFRYLSQAGAGLMELDYLPSDKVYEDEHPND
LPYYWNIAPNMDATITPHYMHRRGNIMWENEFRYLSQAGAGLMELDYLPSDKVYEDEHPND
LPYYWNIAPNMDATITPHYMHRRGNIMWENEFRYLSQAGAGLMELDYLPSDKVYEDEHPND
DSSRRWLFYWNHSGVMDQVWRFNVDYTKVSDPSYFNDFDNKYGSSTDGYATQKFSVGYAVQ
DSSRRWLFYWNHSGVMDQVWRFNVDYTKVSDPSYFNDFDNKYGSSTDGYATQKFSVGYAVQ
DSSRRWLFYWNHSGVMDQVWRFNVDYTKVSDPSYFNDFDNKYGSSTDGYATQKFSVGYAVQ
NFNATVSTKQFQVFSEQNTSSYSAEPQLDVNYYQNDVGPFDTRIYGQAVHFVNTRDDMPEA
NFNATVSTKQFQVFSEQNTSSYSAEPQLDVNYYQNDVGPFDTRIYGQAVHFVNTRDDMPEA
NFNATVSTKQFQVFSEQNTSSYSAEPQLDVNYYQNDVGPFDTRIYGQAVHFVNTRDDMPEA
TRVHLEPTINLPLSNNWGSINTEAKLLATHYQQTNLDWYNSRNTTKLDESVNRVMPQFKVD
TRVHLEPTINLPLSNNWGSINTEAKFLATHYQQTNLDWYNSRNTTKLDESVNRVMPQFKVD
TRVHLEPTINLPLSNNWGSINTEAKFLATHYQQTNLDWYNSRNTTKLDESVNRVMPQFKVD
GKMVFERDMEMLAPGYTQTLEPRAQYLYVPYRDQSDIYNYDSSLLQSDYSGLFRDRTYGGL
GKMVFERDMEMLAPGYTQTLEPRAQYLYVPYRDQSDIYNYDSSLLQSDYSGLFRDRTYGGL
GKMVFERDMEMLAPGYTQTLEPRAQYLYVPYRDQSDIYNYDSSLLQSDYSGLFRDRTYGGL
DRIASANQVTTGVTSRIYDDAAVERFNISVGQIYYFTESRTGDDNITWENDDKTGSLVWAG
DRIASANQVTTGVTSRIYDDAAVERFNISVGQIYYFTESRTGDDNITWENDDKTGSLVWAG
DRIASANQVTTGVTSRIYDDAAVERFNISVGQIYYFTESRTGDDNITWENDDKTGSLVWAG
DTYWRISERWGLRGGIQYDTRLDNVATSNSSIEYRRDEDRLVQLNYRYASPEYIQATLPKY
DTYWRISERWGLRGGIQYDTRLDNVATSNSSIEYRRDEDRLVQLNYHYASPEYIQATLPKY
DTYWRISERWGLRGGIQYDTRLDNVATSNSSIEYRRDEDRLVQLNYHYASPEYIQATLPKY
YSTAEQYKNGISQVGAVASWPIADRWSIVGAYYYDTNANKQADSMLGVQYSSCCYAIRVGY
YSTAEQYKNGISQVGAVASRPIADRWSIVGAYYYDTNANKQADSMLGVQYSSCCYAIRVGY
YSTAEQYKNGISQVGAVASRPIADRWSIVGAYYYDTNANKQADSMLGVQYSSCCYAIRVGY
ERKLNGWDNDKQHAVYDNAIGFNIELRGLSSNYGLGTQEMLRSNILPYQNTL
ERKLNGWDNDKQHAVYDNAIGFNIELRGLSSNYGLGTQEMLRSNILPYQNTL
ERKLNGWDNDKQHAVYDNAIGFNIELRGLSSNYGLGTQEMLRSNILPYQNTL

B

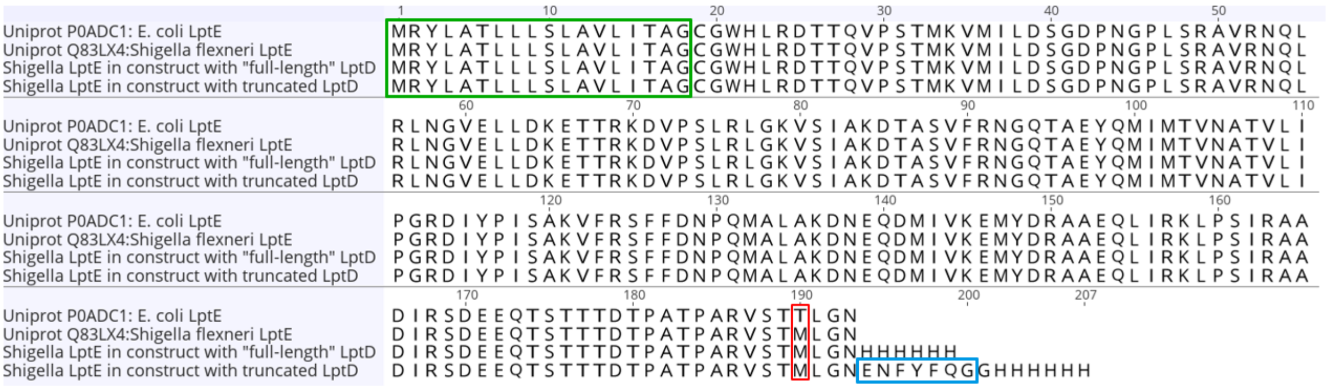
Uniprot P0ADC1: E. coli LptE
Uniprot Q83LX4:Shigella flexneri LptE
Shigella LptE in construct with "full-length" LptD
Shigella LptE in construct with truncated LptD
MRYLATLLLSLAVLITAGCGWHLRDTTQVPSTMKVMILDSGDPNGPLSRAVRNQL
RLNGVELLDKETTRKDVPSLRLGKVSIAKDTASVFRNGQTAEYQMIMTVNATVLI
PGRDIYPISAKVFRSFFDNPQMALAKDNEQDMIVKEMYDRAAEQLIRKLPSIRAA
DIRSDEEQTSTTTDTPATPARVSTTLGN
DIRSDEEQTSTTTDTPATPARVSTMLGN
DIRSDEEQTSTTTDTPATPARVSTMLGNHHHHHH
DIRSDEEQTSTTTDTPATPARVSTMLGNENFYFQGGHHHHHH

C

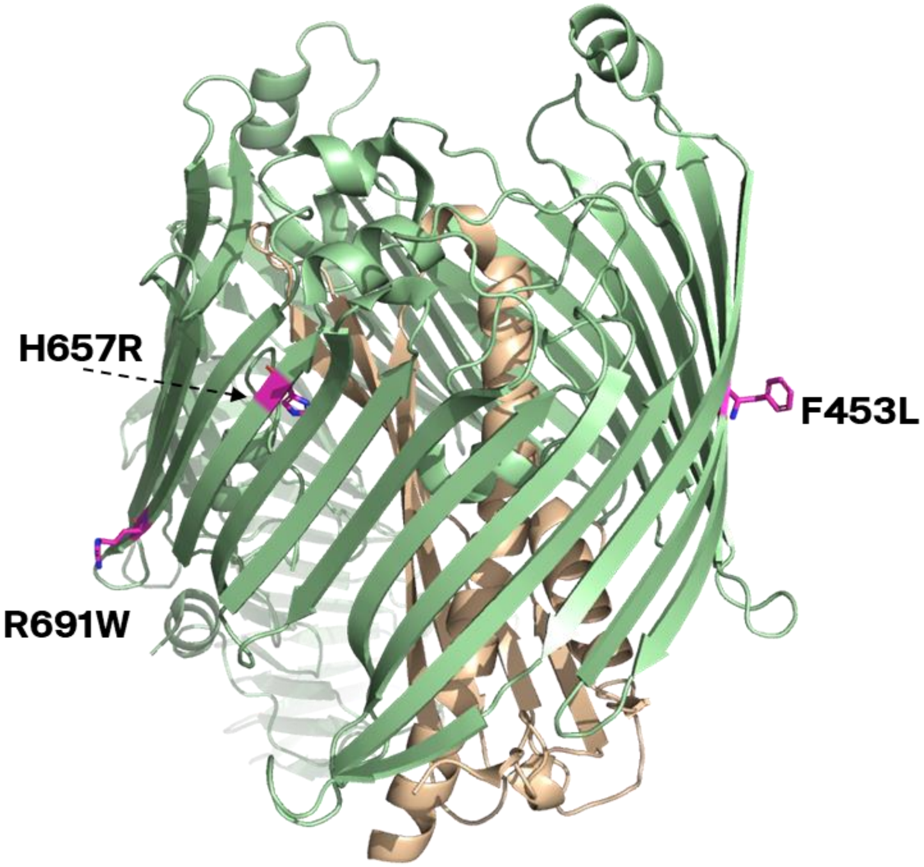

Figure S1. Alignment of the sequences of the Uniprot entries for *E. coli* (Uniprot accession codes: P31554, P0ADC1) and *Shigella flexneri* (Uniprot accession codes: Q83SQ0, Q83LX4) LptD (A) and LptE (B) with the constructs used in this study. Note the "full length" Shigella LptDE (SfLptDE$_{FL}$) contains the same LptD sequence as the Uniprot entry for SfLptD (Uniprot accession code: Q83LX4). There are 3 amino acid variants (red boxes) between *Shigella flexneri* and *E. coli* LptD (Phe435Leu, His657Arg, Arg691Trp), and one variant in LptE (Met190Thr). Numbering used is the translated sequence, before posttranslational modification. Residues 1-24 of LptD and 1-18 of LptE are signal peptide sequences, cleaved during post-translational modification, shown in green boxes. The truncated sequence of LptD starts with an alanine (a "scar" derived from the signal peptide cleavage). The LptE constructs have a C-terminal hexahistidine tag, with an additional Tobacco Etch Virus (TEV) protease cleavage recognition sequence (blue box) found before the hexahistidine tag in the LptE complexed to the truncated LptD. (**C**) Sequence variants (dark blue) between *E. coli* and *Shigella* LptDE shown on a model of SfLptDE (PDB ID: 4Q35[2]). The Met190Thr variants in LptE is found in a disordered region and is not shown.

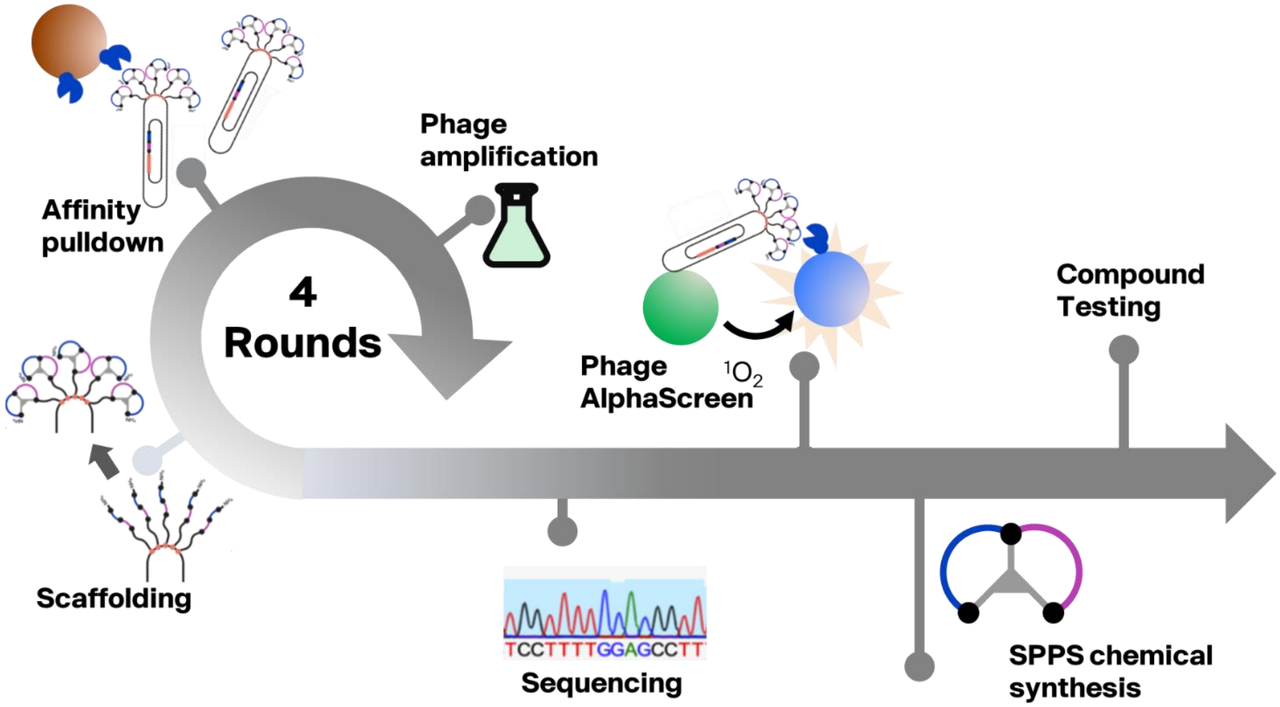

Figure S2. Overview of the phage selection process. The process was initiated with the scaffolding of linear peptides displayed on phage particles. These were exposed to the biotinylated target molecule which was bound via streptavidin to magnetic beads. Affinity pulldown was used to select phage particles expressing scaffolded peptides bound to the target molecule. These phage were then amplified overnight by infection into *E. coli* cells. This process was repeated 4 times with decreasing target concentrations in subsequent rounds, then the phage genomes were sequenced by next generation sequencing (NGS) or Sanger sequencing. Optionally, monoclonal isolates were sequenced and then tested in a "AlphaScreen" assay as described previously.[3,4] Clones selected for chemical synthesis were selected based on their diversity of sequence, frequency of observation and (where run) AlphaScreen signal.

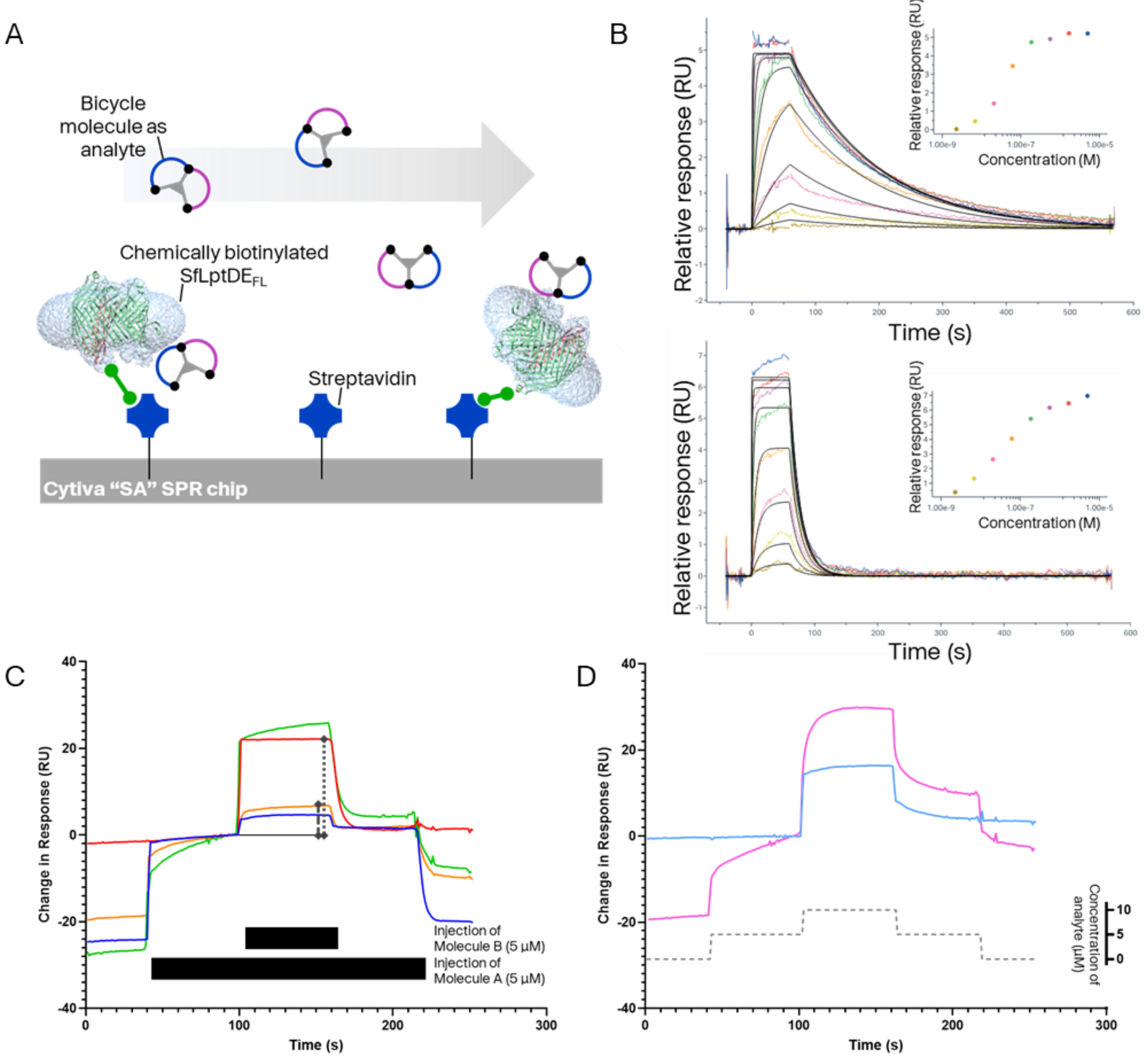

E

	1	2	3	4	5	6	7	8	9	10	11	12	13	14	15	16	17
1	2	2	4	4	2	2	4	2	4	3	2	2	2	3	4	2	4
2	2	2	2	3	2	4	4	4	5	3	3	6	6	6	6	6	6
3	4	2	3	5	5	4	5	3	6	5	4	4	4	5	6	4	6
4	4	3	5	3	4	3	6	3	6	6	3	4	4	5	6	4	6
5	2	2	5	4	3	2	4	2	5	3	3	4	4	5	6	4	6
6	2	4	4	3	2	3	4	4	6	2	2	6	4	6	4	6	6
7	4	4	5	6	4	4	3	3	6	5	3	5	5	5	5	5	5
8	2	4	3	3	2	4	3	2	5	2	3	6	6	6	6	6	6
9	4	5	6	6	5	6	6	5	3	5	4	6	6	6	6	6	6
10	3	3	5	6	3	2	5	2	5	3	2	4	4	5	6	4	6
11	2	3	4	3	3	2	3	3	4	2	2	4	4	2	4	2	2
12	2	6	4	4	4	6	5	6	6	4	4	0	6	6	6	6	6
13	2	6	4	4	4	4	5	6	6	4	4	6	3	4	5	4	4
14	3	6	5	5	5	6	5	6	6	5	2	6	4	3	4	6	6
15	4	6	6	6	6	4	5	6	6	6	4	6	5	4	3	4	4
16	2	6	4	4	4	6	5	6	6	4	2	6	4	6	4	3	6
17	4	6	6	6	6	6	5	6	6	6	2	6	4	6	4	6	3

Figure S3 SPR binding and competition assays. A) Schematic of the SPR experimental set up. Randomly biotinylated $SfLptDE_{FL}$ was immobilised to a Cytiva SA SPR chip to a surface density of approximately 1000 RU. After immobilisation, bicycle molecules were injected over the SPR surface, and the association and dissociation phase were recorded. B) Example sensorgrams of two compounds Compound **4** (competition bin 1) (top- affinity reported as 16 nM) and Compound **16** (competition bin 3) (bottom- affinity reported as 35 nM). Responses at steady state (response measured 5 seconds before end of dissociation phase) are shown inset, showing a saturation of the response. The concentration titration used here was 8 concentrations of the peptide (between 2.3 and 5000 nM, in 3-fold increments). Sensorgrams were double reference corrected using the reference flow cell and blank buffer injections, and then solvent corrected. C) Example competition binning by SPR experiment. In each trace, Molecule B (5 µM; compound Compound **5**, competition bin 1 in this example) was injected during the period indicated by the short black bar, and Molecule A (5 µM; Compound **16**, competition bin 3 in the green trace, Compound **3**, competition bin 1 in the orange trace) or control (5 µM compound Compound **5** in the blue trace or buffer only in the red trace) was injected during the period indicated by the long black bar. The change in response (R_B) was measured, where R_B = [response level during injection of Molecule B]-[response level before injection of Molecule B] (indicated by the dashed and dotted lines). If the R_B measured after injection of Molecule A was < 80% of R_B after injection of the buffer-only control, the interaction was scored as "competitive". In the example shown, the R_B (dotted line) when Molecule A was Compound **3** (orange trace) shows a large (>80%) reduction, whilst the R_B when Molecule A is Compound 16 (green trace) was similar to the R_B for the buffer-only control (dotted line). Injection of the same compound (e.g. here we show Compound **5**, blue trace) as both Molecule A and Molecule B was used as a positive control for competition. D) An example of an "ambiguous" interaction. Shown is the buffer only control for Compound **12**, (cyan) and the equivalent positive control trace (i.e. Compound **12** used as Molecule A and Molecule B, Magenta). The concentration of Compound **12** during the positive control experiment is indicated (right axis). In this example, R_B for the positive control was greater than the R_B of the buffer-only control (i.e., rather than preventing the binding of further peptide, the signal was increased by the pre-injection of "Molecule A" peptide). This was due to Compound **12** not saturating the protein, as the interaction also has a non-specific, non-saturable component which appears at high concentrations such as the 10 µM used here. Evidence for this component was also seen in the lack of return to the 0 RU baseline following the end of the Molecule B injection (at circa 160 s). In general, this type of non-specific signal was likely to occur on a peptide-by-peptide basis, and at high concentrations of peptide (>5 µM) and will "mask" the observation of competition between peptides that bind to the same competition bin. For this reason, all interactions with Compound **12** were excluded from the results. E) Numbers of replicates for each of the observation in Figure 2 in the main text. Dashed lines indicate the competition bins shown in Figure 2 in the main text. All interactions including Compound **12** are highlighted in gray as these were all excluded from the results. As in the main text, peptides highlighted in red are those which we determined a structure in complex with the protein.

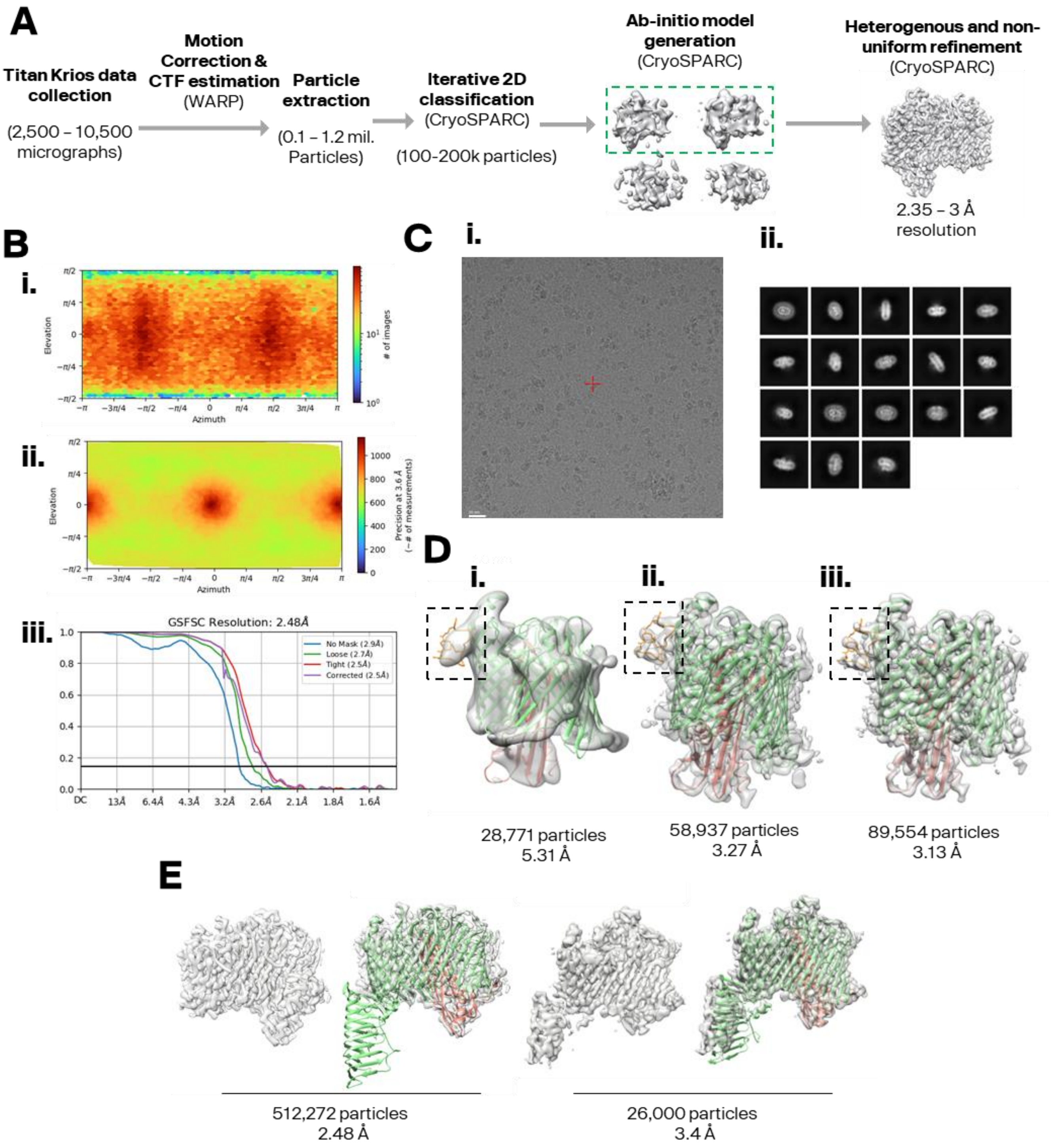
A
Titan Krios data collection
(2,500 – 10,500 micrographs)
Motion Correction & CTF estimation
(WARP)
Particle extraction
(0.1 – 1.2 mil. Particles)
Iterative 2D classification
(CryoSPARC)
(100-200k particles)
Ab-initio model generation
(CryoSPARC)
Heterogenous and non-uniform refinement
(CryoSPARC)
2.35 – 3 Å resolution
B
i.
ii.
iii.
GSFSC Resolution: 2.48Å
No Mask (2.9Å)
Loose (2.7Å)
Tight (2.5Å)
Corrected (2.5Å)
C
i.
ii.
D
i.
ii.
iii.
28,771 particles
5.31 Å
58,937 particles
3.27 Å
89,554 particles
3.13 Å
E
512,272 particles
2.48 Å
26,000 particles
3.4 Å

Figure S4: Cryo-EM data collection and processing of Bicycle molecule-bound SfLptDE. **A**) image processing workflow for purified SfLptDE with bound Bicycle molecules. Initially, close to 10,000 micrographs were collected for a final electron density reconstruction under 3 Å, but this was reduced to 2,500 – 3000 micrographs per data collection without a compromise in final resolution. WARP was used to streamline motion correction, CTF estimation and extracting particles. Particles were then imported into CryoSPARC and underwent iterative rounds of 2D classification to remove junk particles. After multiple *ab initio* and refinement steps, final 3D reconstruction maps were reached with a resolution between 2.5 – 3 Å per dataset **B)** Particle distribution of **i:** viewing direction distribution and **ii:** posterior precision directional distribution and **iii:** cryo-EM FSC cure of a typical representative LptDE-Bicycle molecule dataset **C: i:** Representative cryo-EM micrograph and **ii**: corresponding selected 2D classes used for 3D map reconstruction**. D:** Electron density reconstructions using data derived from 1 **(i),** 2**(ii)** and 3 **(iii)** hours' worth of micrograph collection. **E:** 3D reconstruction maps generated from 2D classes and *ab initio* modelling, with a small subset of particles (~5%) showing refinable density for the LptD N-terminal domain. All data shown here are derived from the Compound **12** dataset, but are representative of all datasets and their associated processing.

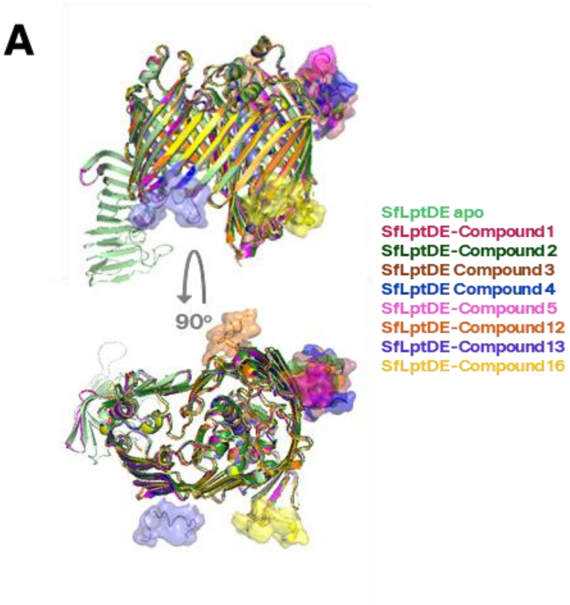

B

RMSD (Å²)	Apo (PDB 4Q35)	Compound1	Compound 2	Compound3	Compound 4	Compound5	Compound12	Compound13	Compound16
**Apo (PDB 4Q35)**		[redacted]	[redacted]	[redacted]	[redacted]	[redacted]	[redacted]	[redacted]	[redacted]
**Compound1**	0.317		[redacted]	[redacted]	[redacted]	[redacted]	[redacted]	[redacted]	[redacted]
**Compound2**	0.382	0.175		[redacted]	[redacted]	[redacted]	[redacted]	[redacted]	[redacted]
**Compound3**	0.401	0.232	0.259		[redacted]	[redacted]	[redacted]	[redacted]	[redacted]
**Compound4**	0.328	0.114	0.164	0.254		[redacted]	[redacted]	[redacted]	[redacted]
**Compound5**	0.363	0.150	0.178	0.243	0.142		[redacted]	[redacted]	[redacted]
**Compound12**	0.384	0.285	0.320	0.267	0.294	0.294		[redacted]	[redacted]
**Compound13**	0.379	0.186	0.218	0.211	0.183	0.200	0.273		[redacted]
**Compound16**	0.376	0.172	0.198	0.208	0.182	0.201	0.286	0.181	

Figure S5: Alignments of SfLptDE bound with a Bicycle molecule, from each identified epitope. Alignments **(A)** of the side (above) and top (below) view of SfLptDE apo-form and bound to each Bicyclic peptide compound. Corresponding root mean squared deviation (RMSD) values **(B)** between the apo structure of SfLptDE (PDB ID: 4Q35[2]) and the bound state with each Bicyclic peptide compound. Low RMSD values and minimal conformational change between structures indicates no structural rearrangement of the core of the LptDE beta-barrel upon Bicyclic peptide binding.

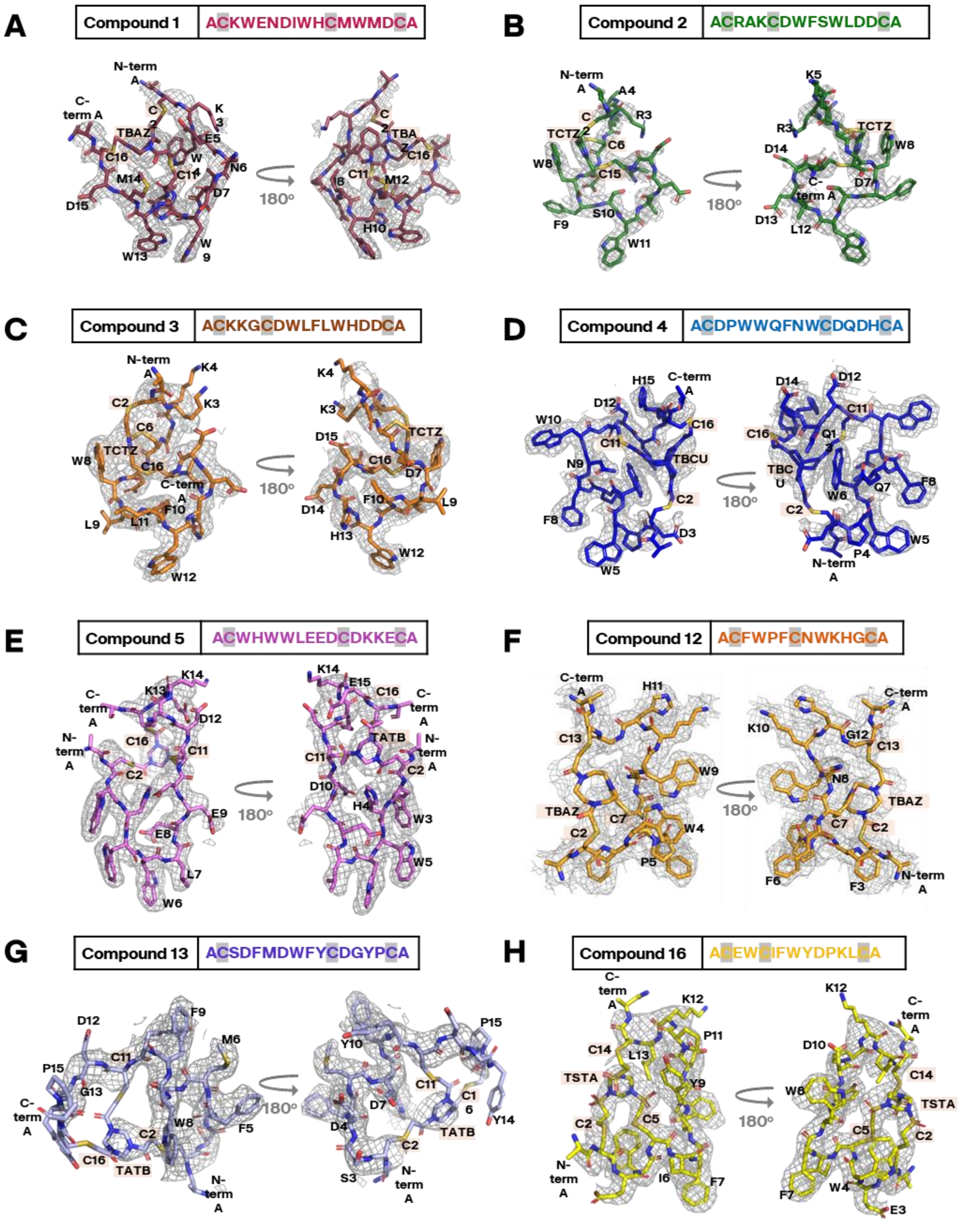

Figure S6: Close-up views of cryo-EM maps showing the electron densities for SfLptDE-binding Bicyclic Peptides. Maps are shown for Compounds **1** (A), **2** (B), **3** (C), **4** (D), **5** (E), **12** (F), **13** (G), and **16** (H). All maps are contoured between 1 – 1.5 σ and clipped around the Bicycle peptide.

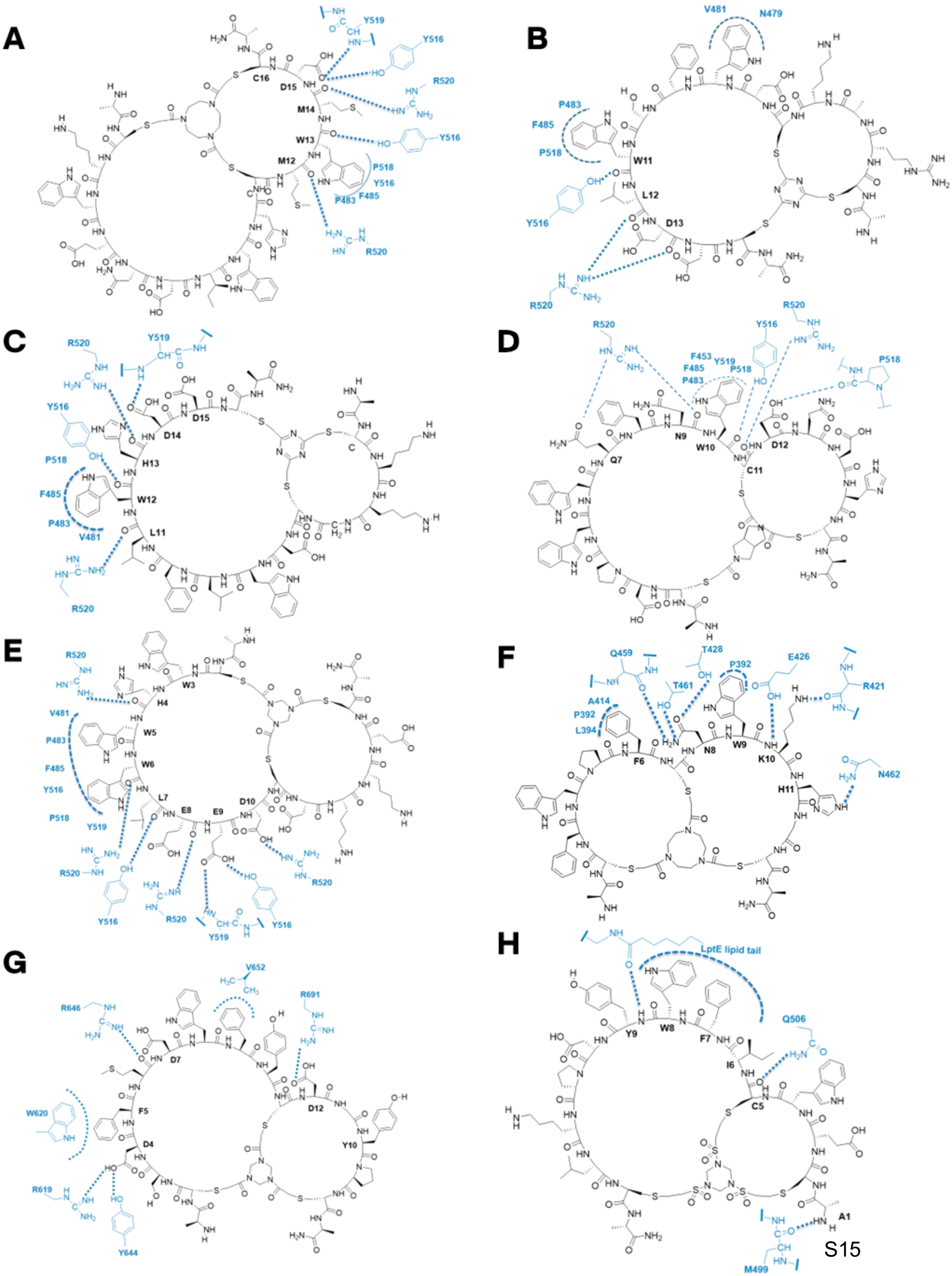
A
Y519
Y516
R520
Y516
P518
Y516
P483
F485
R520
C16
D15
M14
W13
M12
B
V481
N479
P483
F485
P518
W11
L12
D13
Y516
R520
C
R520
Y519
Y516
P518
F485
P483
V481
R520
D14
D15
H13
W12
L11
D
R520
Y516
R520
F453
Y519
F485
P518
P483
P518
N9
W10
Q7
D12
C11
E
R520
V481
P483
F485
Y516
P518
Y519
R520
Y516
R520
Y519
Y516
R520
W3
H4
W5
W6
L7
E8
E9
D10
F
Q459
T428
P392
E426
R421
A414
P392
L394
T461
N462
F6
N8
W9
K10
H11
G
V652
R646
R691
W620
R619
Y644
D7
F5
D4
D12
Y10
H
LptE lipid tail
Q506
Y9
W8
F7
I6
C5
A1
M499

Figure S7: 2D representations of each Bicyclic peptide binder and the contacts made with SfLptDE, based on the cryo-EM reconstructions referred to in Figures 3, 4 and S6. Representations are shown for Compounds **1** (A), **2** (B), **3** (C), **4** (D), **5** (E), **12** (F), **13** (G), and **16** (H), with the Bicycle peptide shown in black and contacting SfLptDE residues shown in blue. Binders in epitope 1 (Compounds **1**-**5**, A-E) all make key contacts via the WxD/EE motifs on the Bicycle peptide, with the Tryptophan docking into a hydrophobic pocket on the LptD β-barrel formed primarily by P483, F485, Y516 and P518 . Additional hydrogen bonding contacts are made with both the backbone and functional groups of the D/E residues with residues R520, Y519 and P518. Mutation of these key motif residues results in abolished or decreased affinity of the peptide to LptDE (Table S3).

A

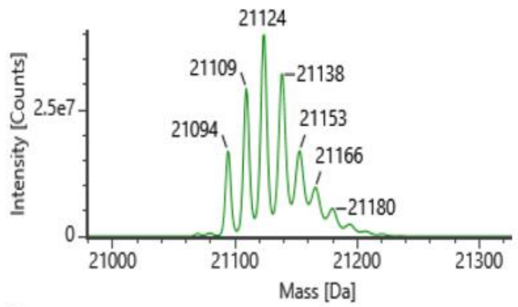

B

Shigella flexneri LptE (Uniprot: Q83LX4) with hexa-histidine tag (Cys19-Asn193-HHHHHH)		
**Expected (Da):**	20308	
**Observed (Da):**	**Deviation (Da)**	**Sequential Addition (Da)**
21094	786	-
21109	801	+15
21124	816	+15
21138	830	+14
21153	845	+15
21166	858	+13
21180	872	+14

C

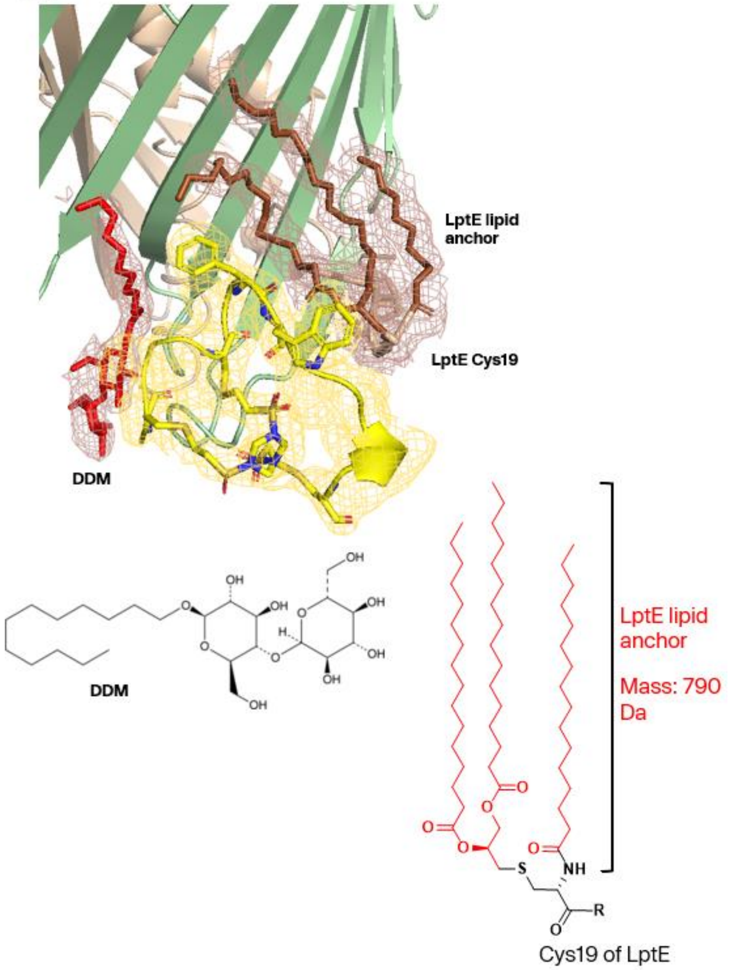

Figure S8: The LptE lipoprotein anchor. A) In the deconvoluted mass spectrum of SfLptE, a cluster of peaks was observed with the lowest with mass = 21,094 Da. This differs from the unmodified protein by 786 Da corresponding to addition of the lipid anchor with various lipid chain lengths differing in length by 1-6 CH_2 groups (14 Da). LC-MS trace and raw mass spectrum of LptE is shown in Figure S5. **B)** Table of mass values detected by LCMS **C)** The cryoEM model of compound **16** in complex with LptDE appears to stabilise the lipid anchor of LptE and enables resolution of part of the lipid anchor. The lipid has a theoretic mass of 790 Da. An example structure of the modified structure of the N-terminal cysteine of LptE where a di-palmatidyl glycerol is reacted with the side chain of Cys19 of LptE, and a molecule of palmitate is reacted with the N-terminal amine. Not all atoms of the lipid tails are present in the pdb model, as there is insufficient density.

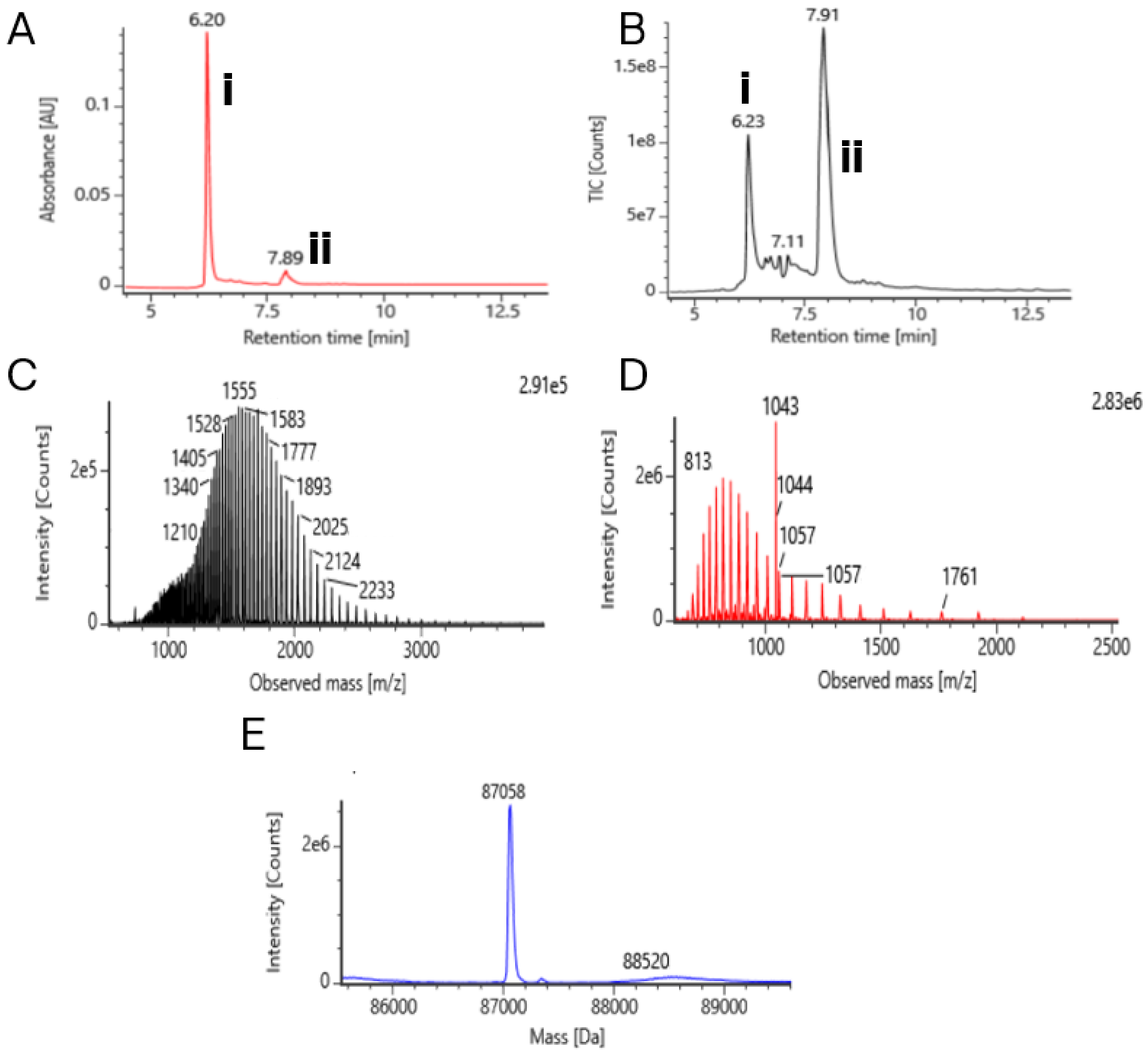

Figure S9: Mass spectrometry of SfLptDE$_{FL}$. A,B) LC-MS trace of the complex as observed by the UV-visible spectrometry and total ion chromatogram (TIC). LptD (i) eluted at approximately 6.2 mins and SfLptE (ii) at 7.9 mins. Raw mass spectrum **(C)** and deconvoluted mass spectrum **(E)** of SfLptD. Raw mass spectrum of SfLptE **(D).** Deconvoluted mass spectrum for SfLptE is in Figure S8A. The mass observed for SfLptD [87,058 Da] corresponds to the expected mass of the construct [87,054 Da]: (Uniprot: Q83SQ0 (Ala25-> Leu784). Residue numbers as per Figure S1.

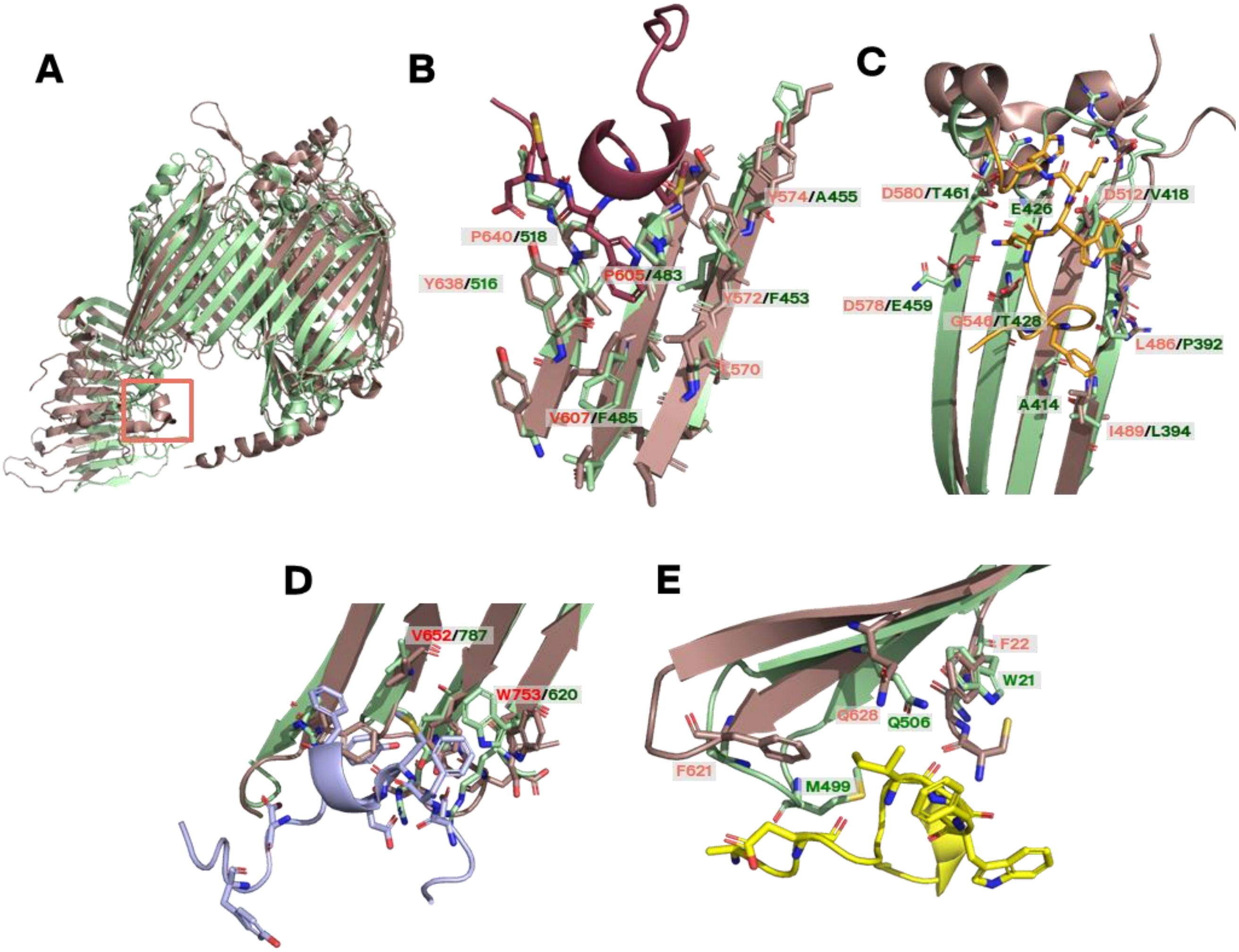

Figure S10: Alignment of *Pseudomonas aeruginosa* LptDE with SfLptDE and Bicycle peptide binders. A) Overall alignment of the crystal structure of SfLptDE (green) (PDB code: 4Q35)[2] and PaLptDE (salmon) (PDB code: 8H1S),[5] showing overall structural homology, with an RMSD of 4.25 Å. The predicted murepavadin binding site on PaLptDE is highlighted with the pink box. B) Local structural alignments of Bicycle peptide epitope representatives B) Compound **1**, C) Compound **12**, D) Compound **13** and E) Compound **16**, at their binding sites on SfLptDE (green), aligned with the cryo-EM model of PaLptDE (81HS). The binding site for members of epitope 1 has highly conserved residues within the hydrophobic pocket that the tryptophan of the WxD/EE motif on the Bicycle peptide binders docks into (B), suggesting that compounds from epitope 1 could also bind the *Pseudomonas* protein. The binding sites for compounds belonging shown in (C-E) have less structural and sequence homology, suggesting these compounds may have a weakened affinity or no binding to the *Pseudomonas aeruginosa* protein, although this has not been confirmed.

Compound HPLC data

Table S4: Summary of peptide HPLC data

Compound	Theoretical Mass (Da)	Observed Mass (Da)	Purity
**1**	2390.8	1195.97	95.9
**2**	2006.3	1003.78	96.2
**3**	2127.4	1064.46	98.7
**4**	2397.6	1199.33	99.2
**5**	2358.6	1180.01	96.3
**6**	2387.7	1194.37	97.3
**7**	2382.7	1192.34	99.9
**8**	2079.3	2079	96.1
**9**	2422.8	2444*	99.3
**10**	2109.4	1055.57	88.5
**11**	2121.3	1060.74	98.9
**12**	1918.3	959.76	98.5
**13**	2200.4	1100.85	99.5
**14**	2278.5	2278.23	100.0
**15**	2291.6	2291.80	96.4
**16**	2204.6	1103.16	95.2
**17**	1749.1	1749.04	95.5
**1a**	2333.7	1167.5	95.7
**1b**	2275.6	1138.1	96.0
**1c**	2332.7	1167	95.4
**1d**	2347.8	1174.4	95.0
**1e**	2346.8	2346.8	97.0
**1f**	2348.7	1174.7	96.0
**1g**	2275.6	1137.9	96.3
**1h**	2324.7	1162.6	96.4
**1i**	2330.7	1165.8	95.1
**1j**	2275.6	1137.7	95.0
**1k**	2330.7	1165.8	95.1
**1l**	2346.8	2346.76	97.6
**2a**	1891.1	1891.53	97.1
**2b**	1962.3	981.5	95.7
**5a**	2243.5	1122.4	97.1
**5b**	2300.6	1150.5	98.7
**5c**	2300.6	1151.3	97.3

*** Theoretical mass plus Na^+**

Compound 1: Molecular weight: 2390.8 Da

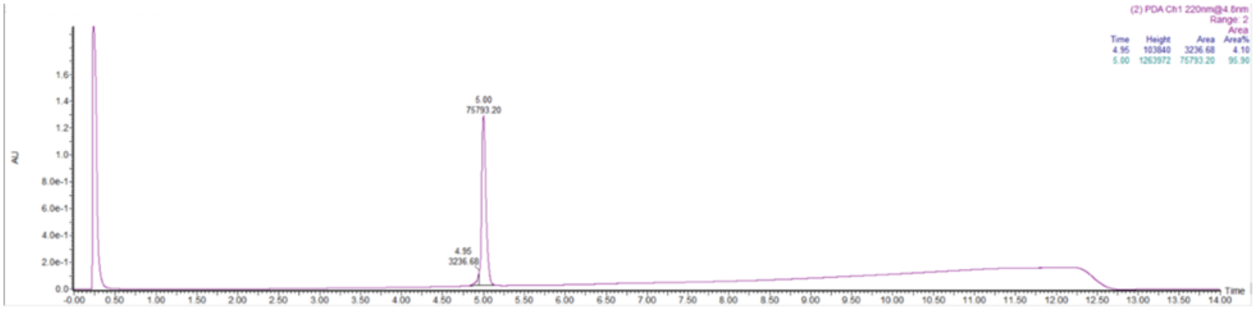

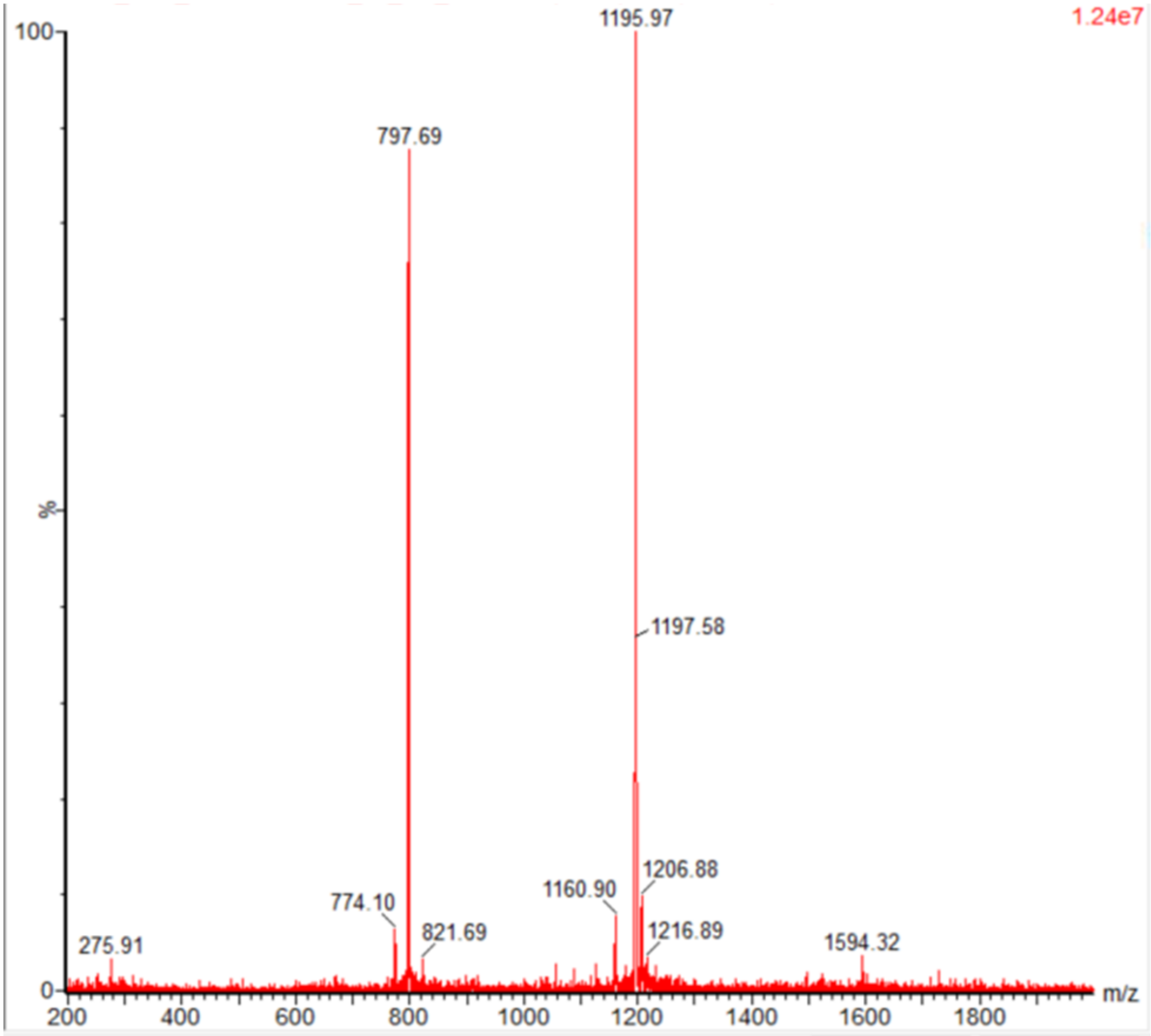

Compound 2: Molecular weight: 2006.2 Da

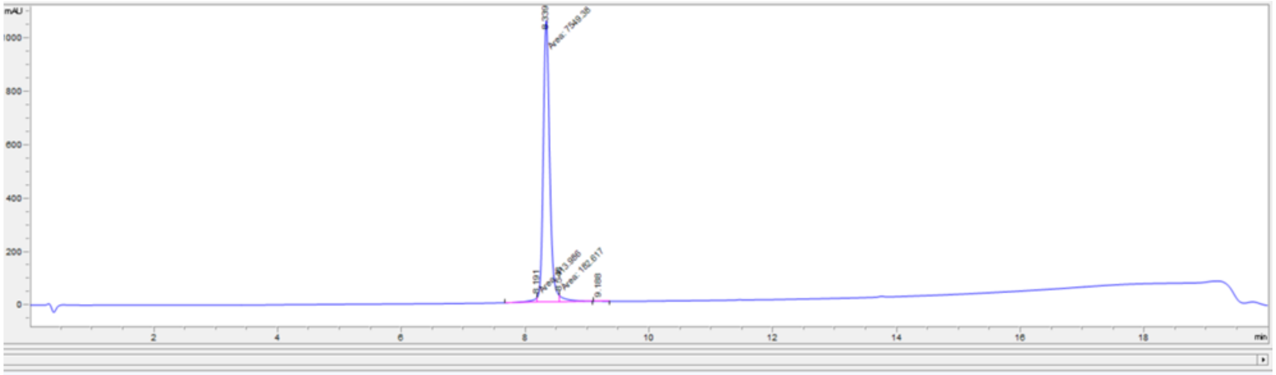

#	Time	Type	Area	Height	Width	Area%	Symmetry
1	8.191	MF	114	17.8	0.1067	1.452	257.11
2	8.339	MF	7549.4	1053.1	0.1195	96.167	0.828
3	8.548	FM	182.6	24.3	0.1255	2.326	0
4	9.188	BB	4.3	7.1E-1	0.0961	0.054	0.759

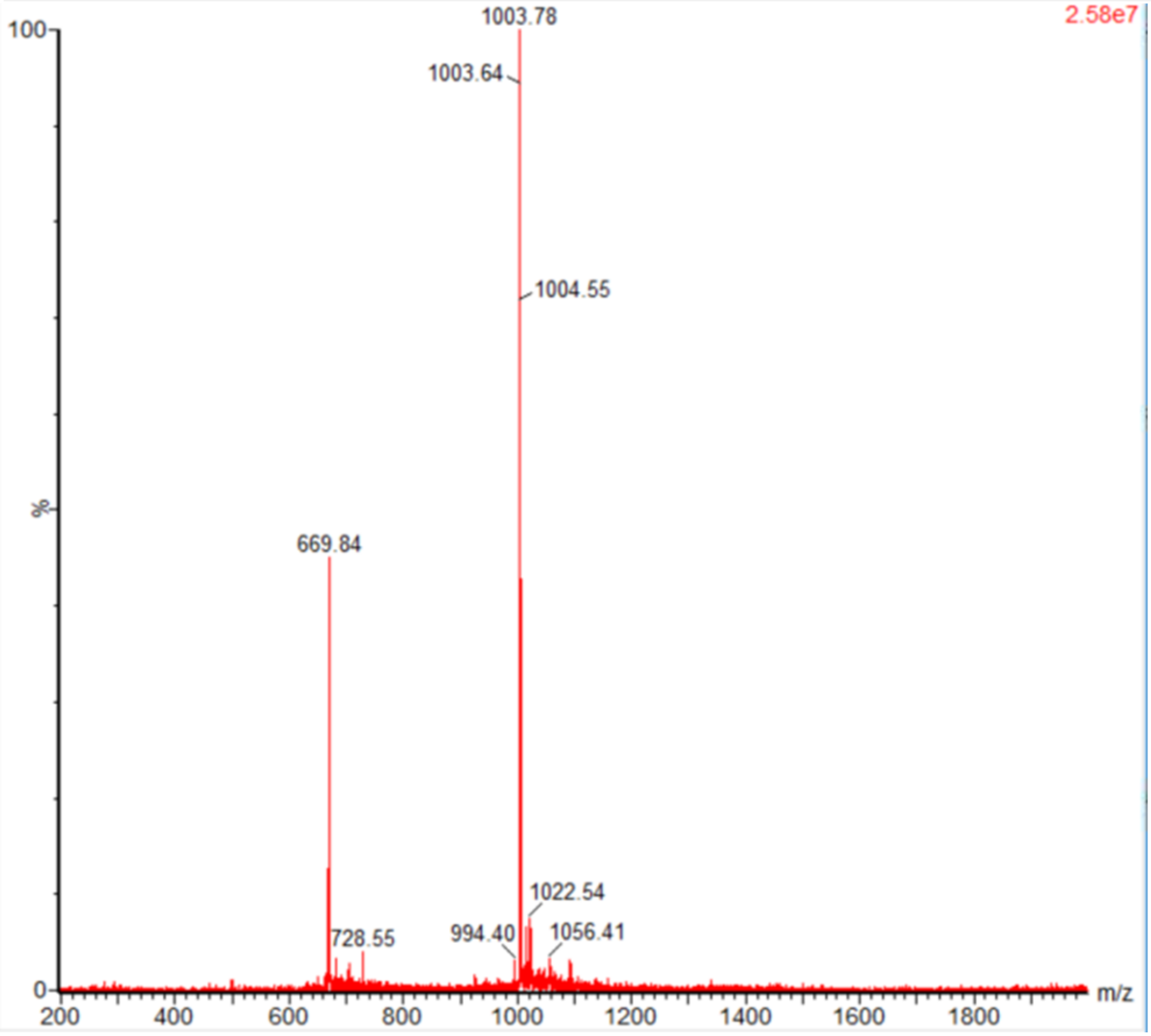

Compound 3: Molecular weight: 2127.4 Da

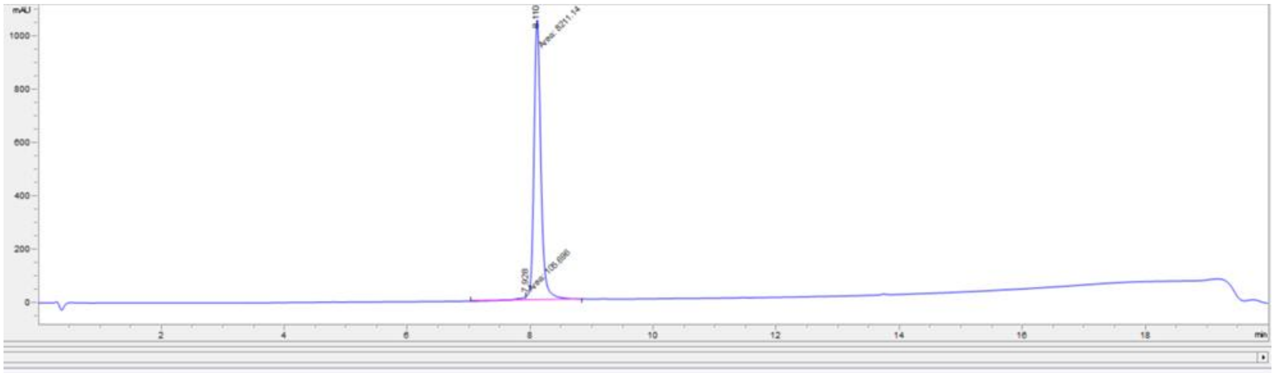

#	Time	Type	Area	Height	Width	Area%	Symmetry
1	7.928	MF	105.7	13	0.1354	1.271	484.208
2	8.11	FM	8211.1	1052.7	0.13	98.729	0.789

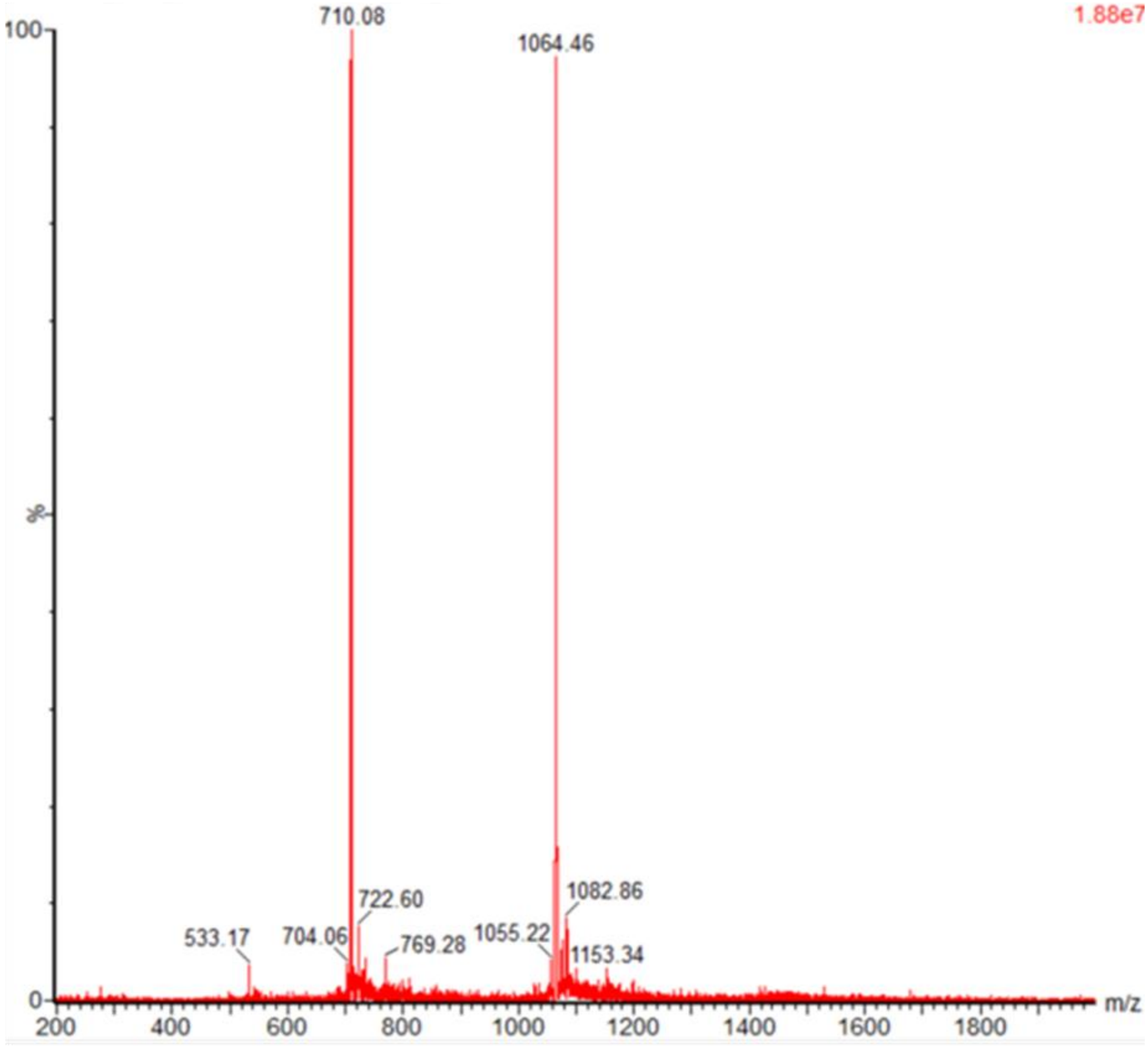

Compound 4: Molecular weight: 2397.6 Da

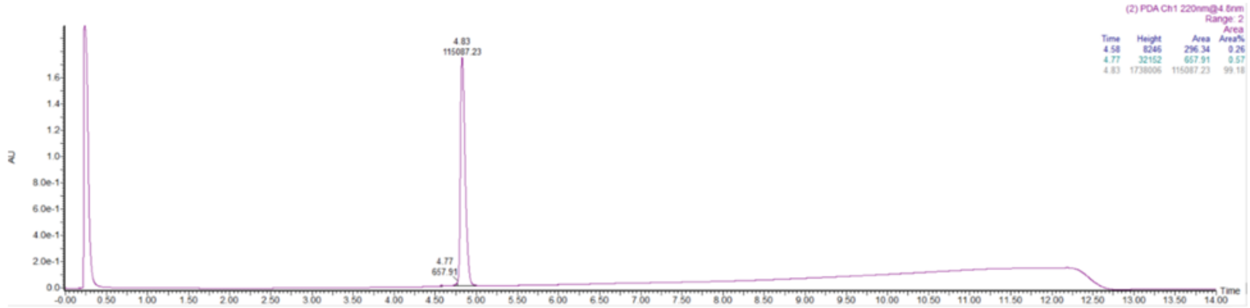

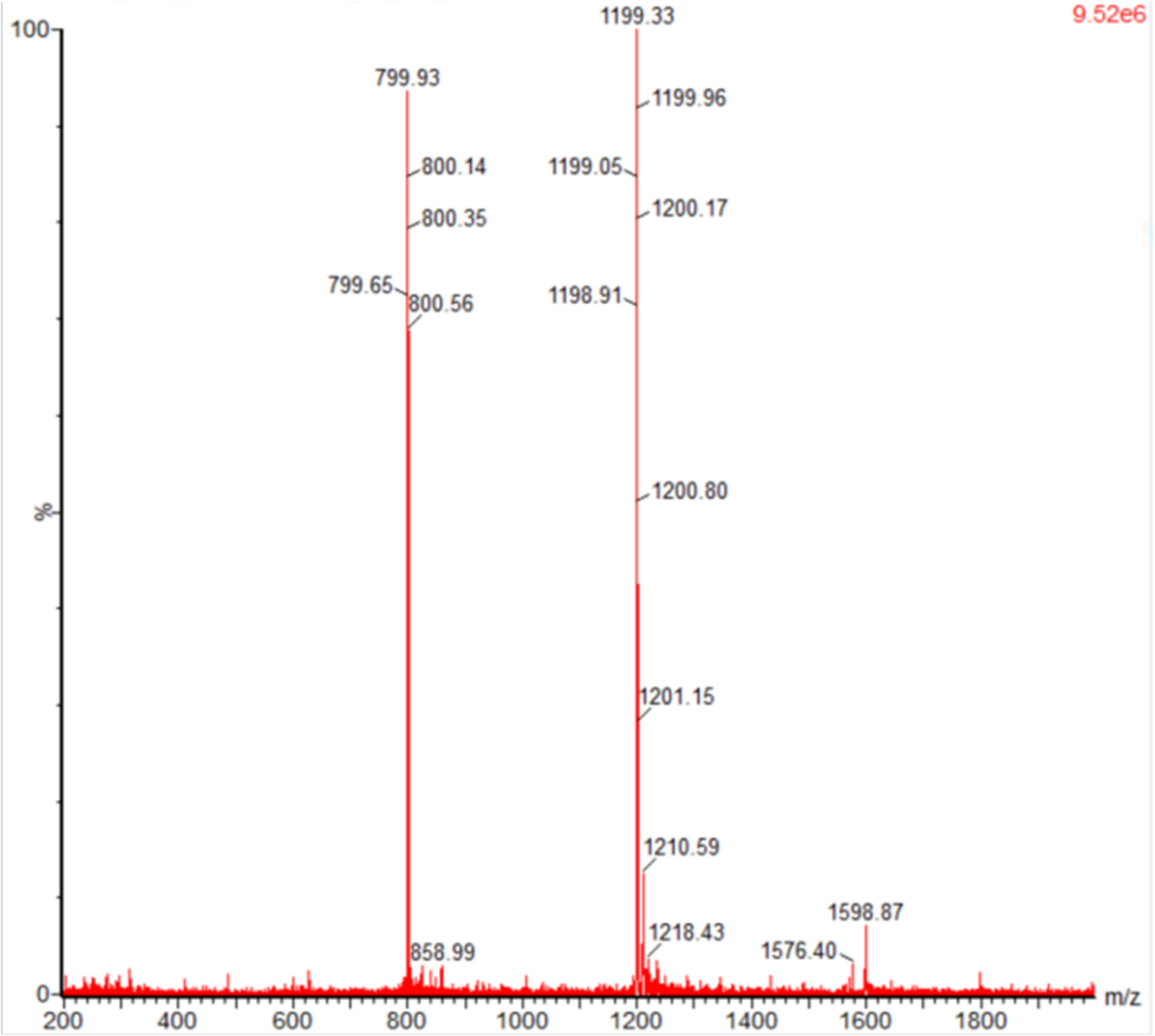

Compound 5: Molecular weight: 2358.6 Da

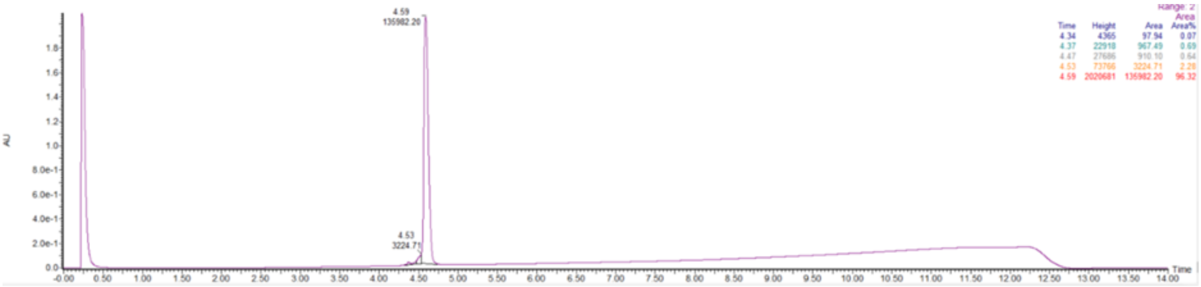

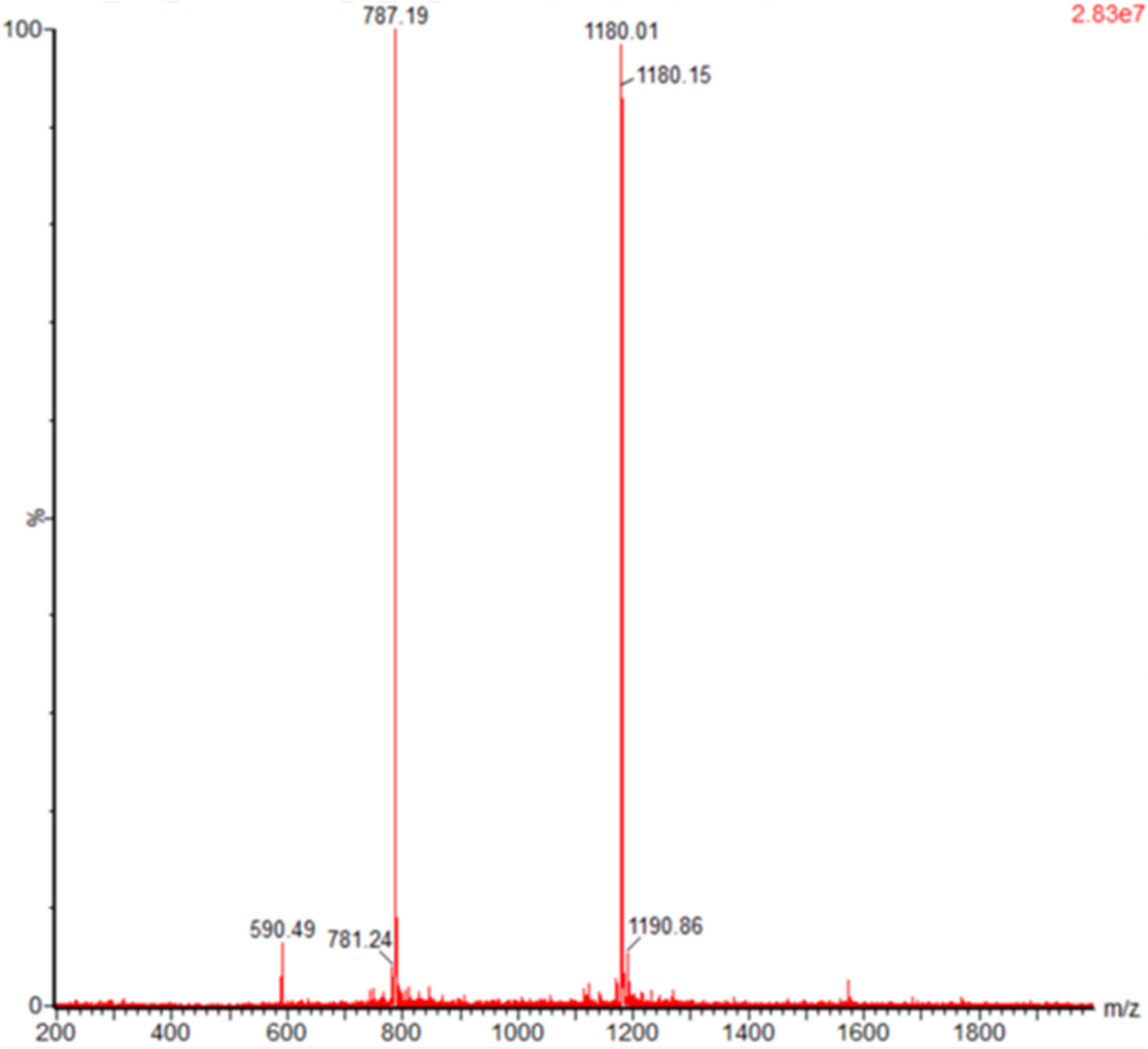

Compound 6: Molecular weight: 2387.7 Da

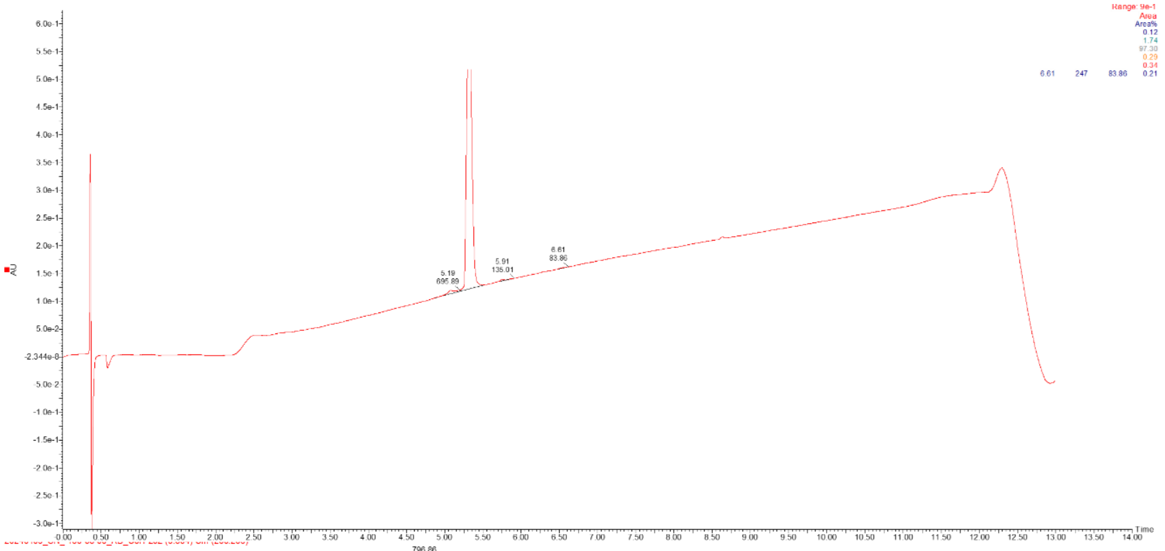

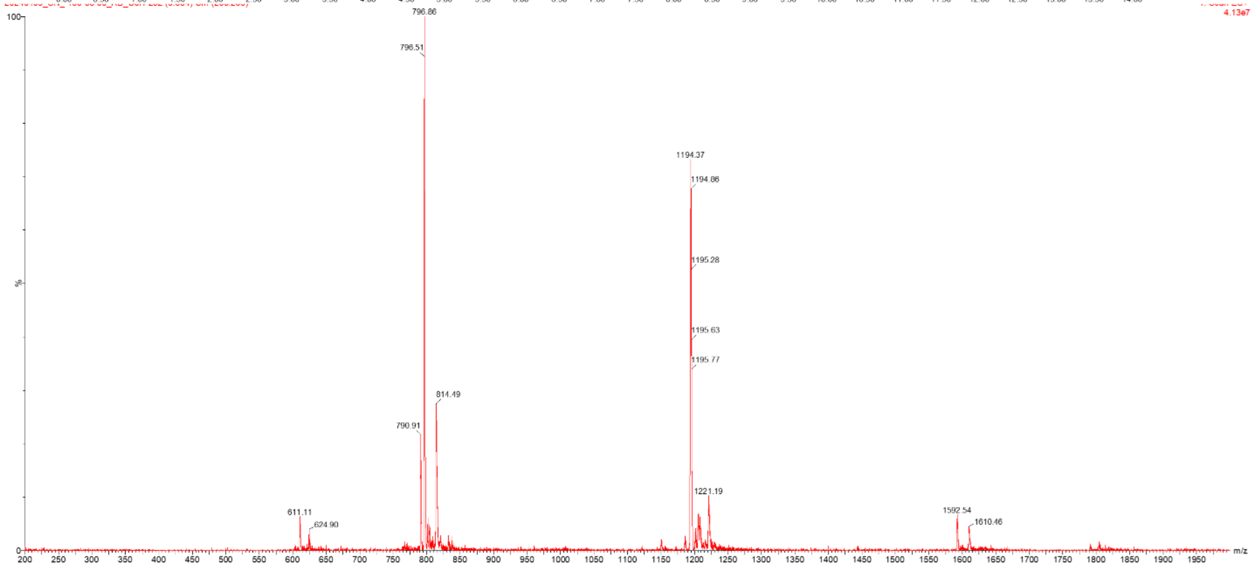

Compound 7: Molecular weight 2382.7 Da

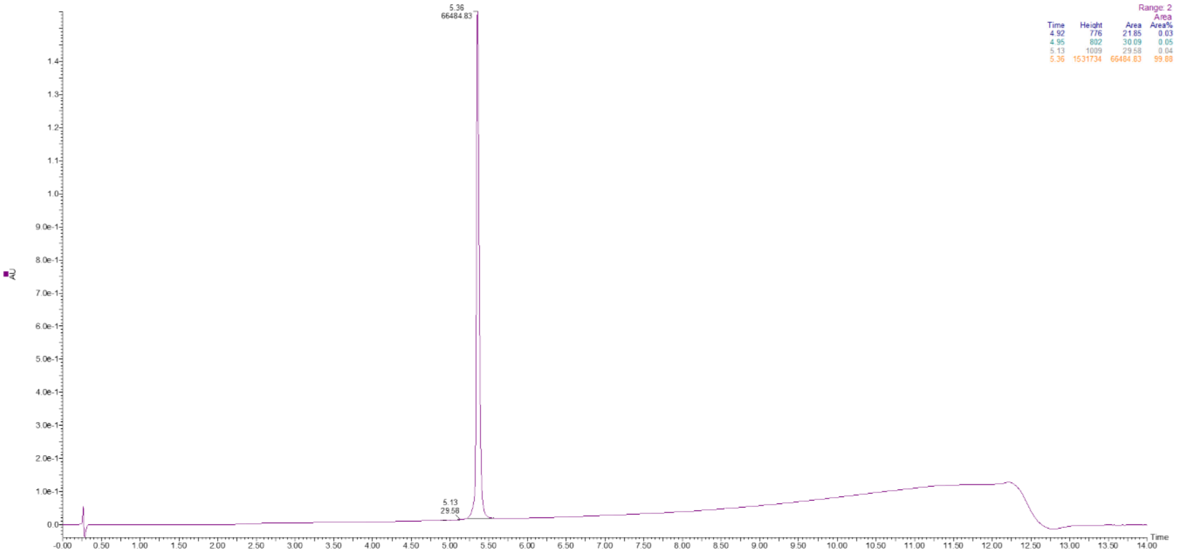

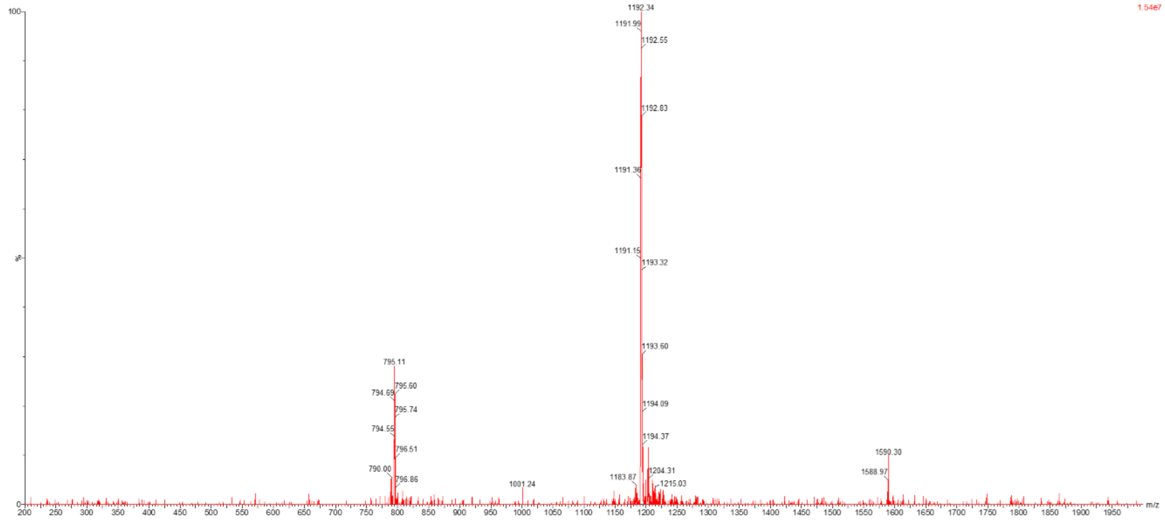

Compound 8: Molecular weight 2079.3 Da

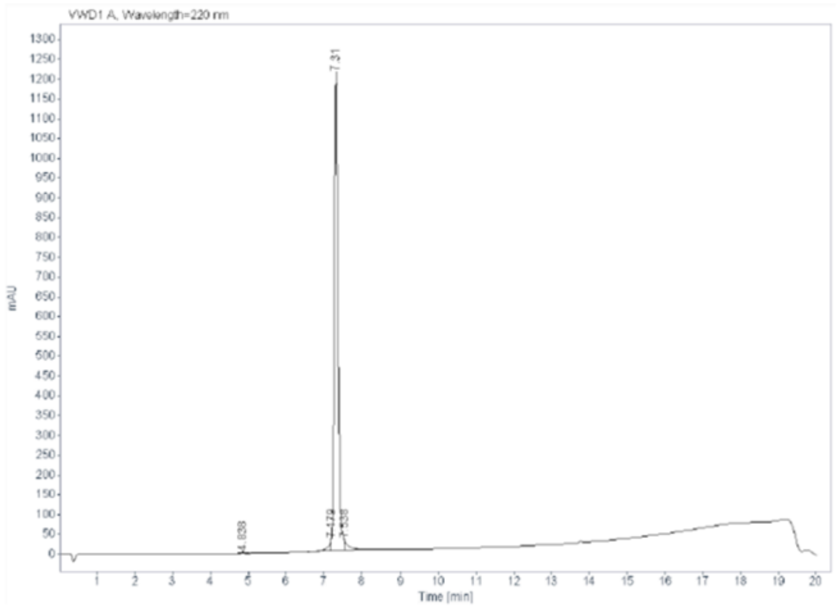

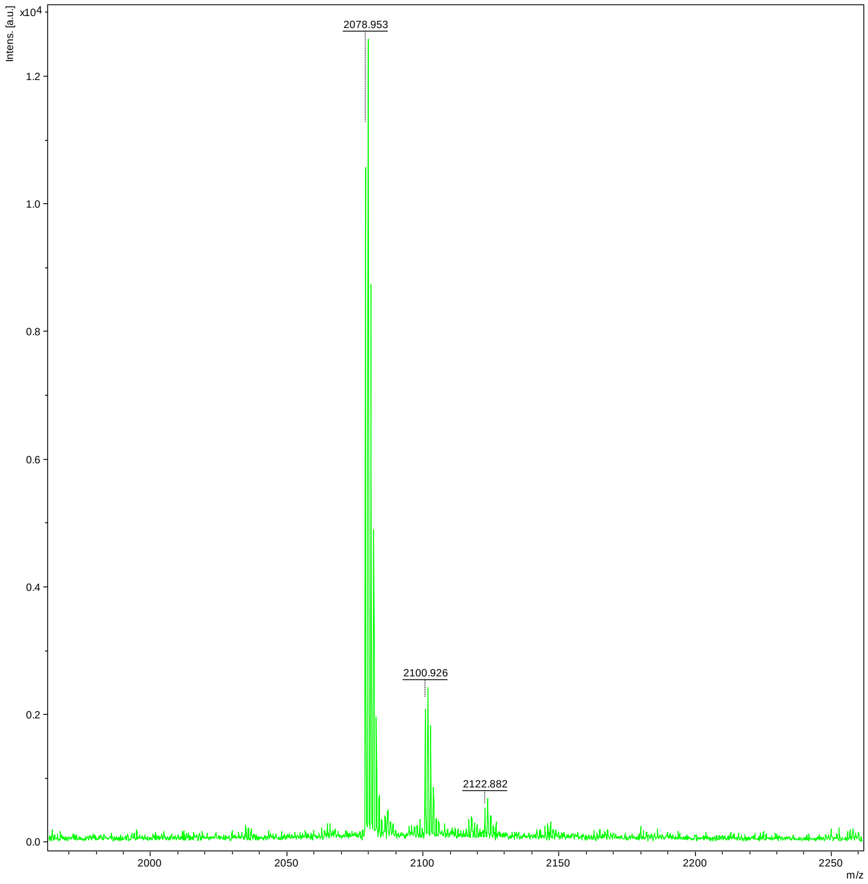

Compound 9: Molecular weight 2422.8 Da

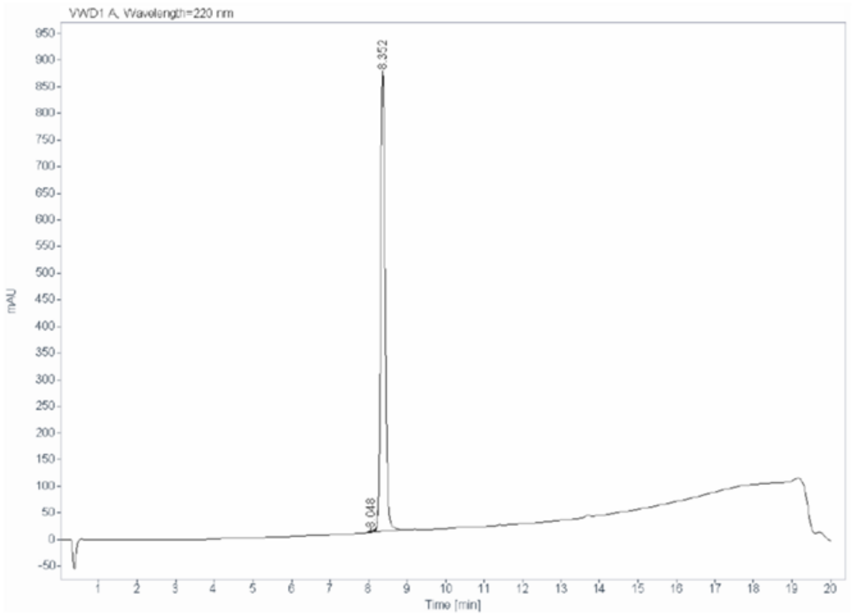

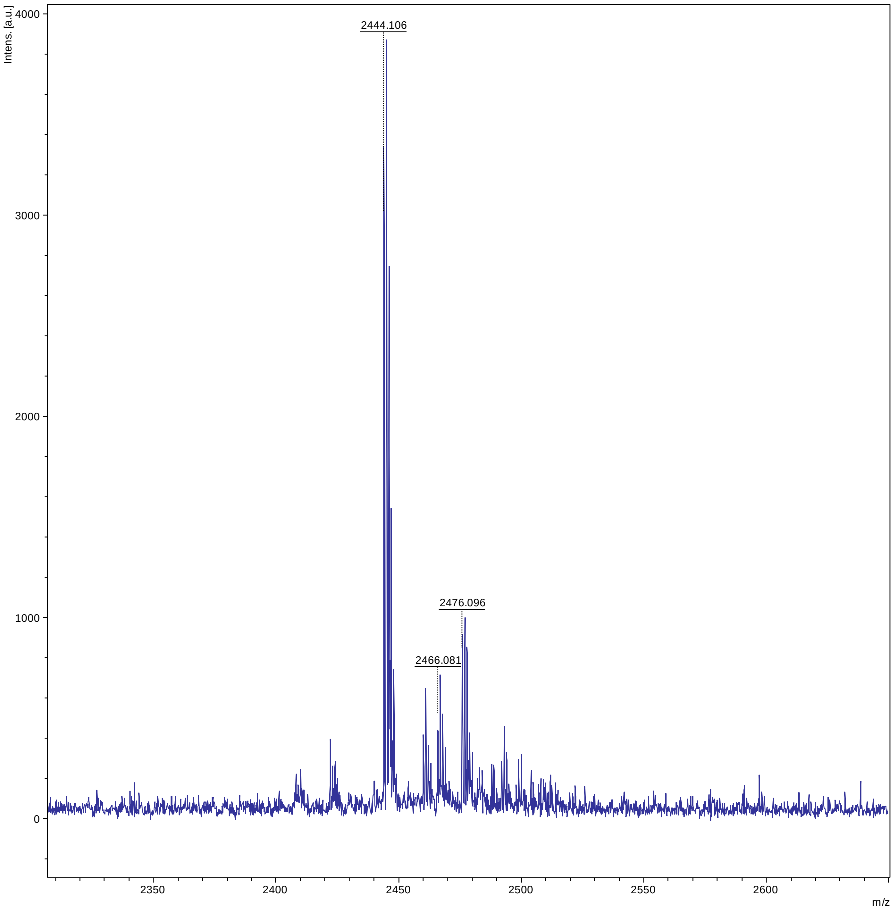

Compound 10: Molecular weight: 2109.4 Da

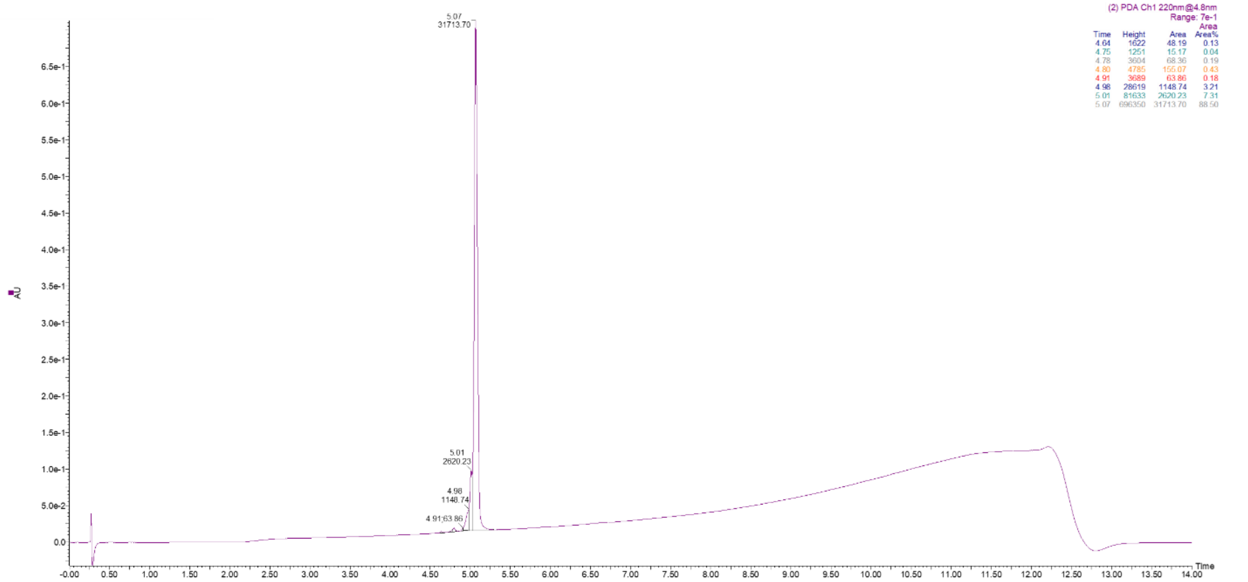

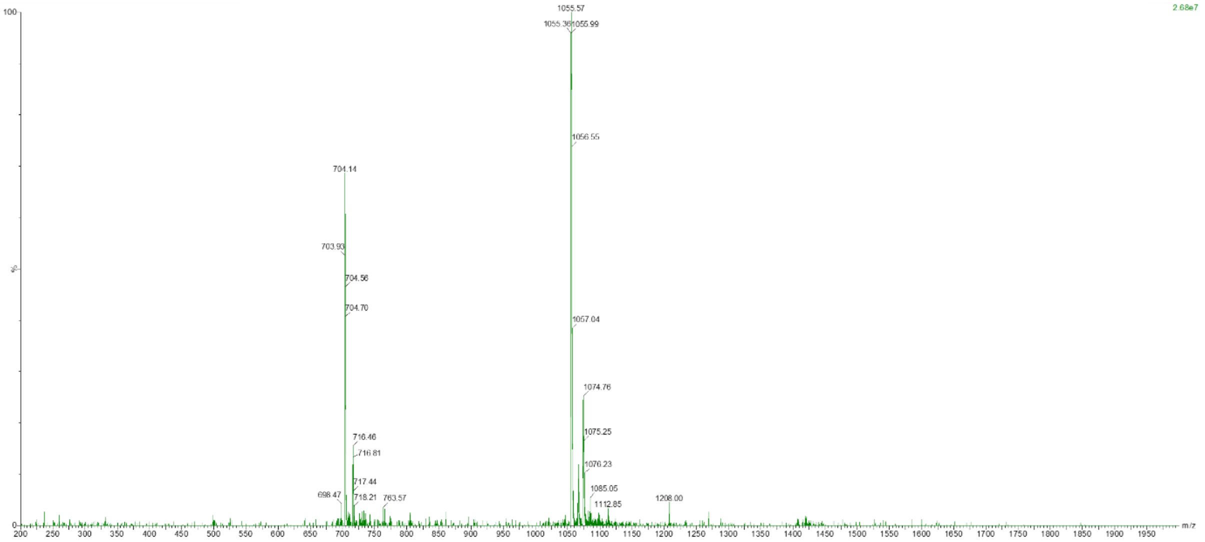

Compound 11: Molecular weight: 2121.3 Da

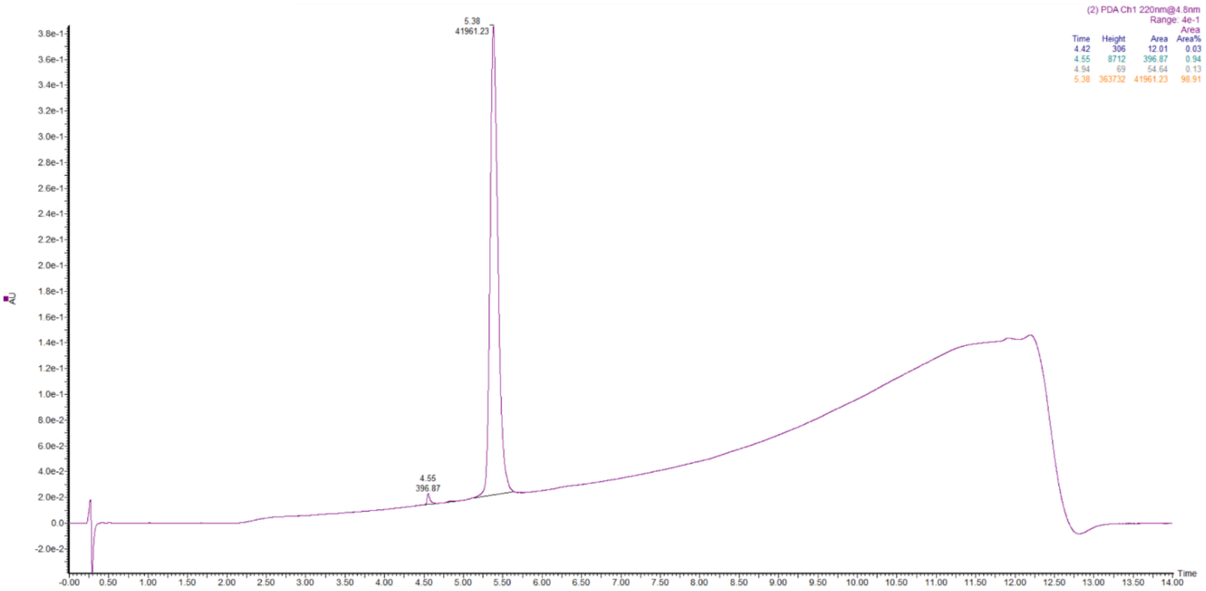

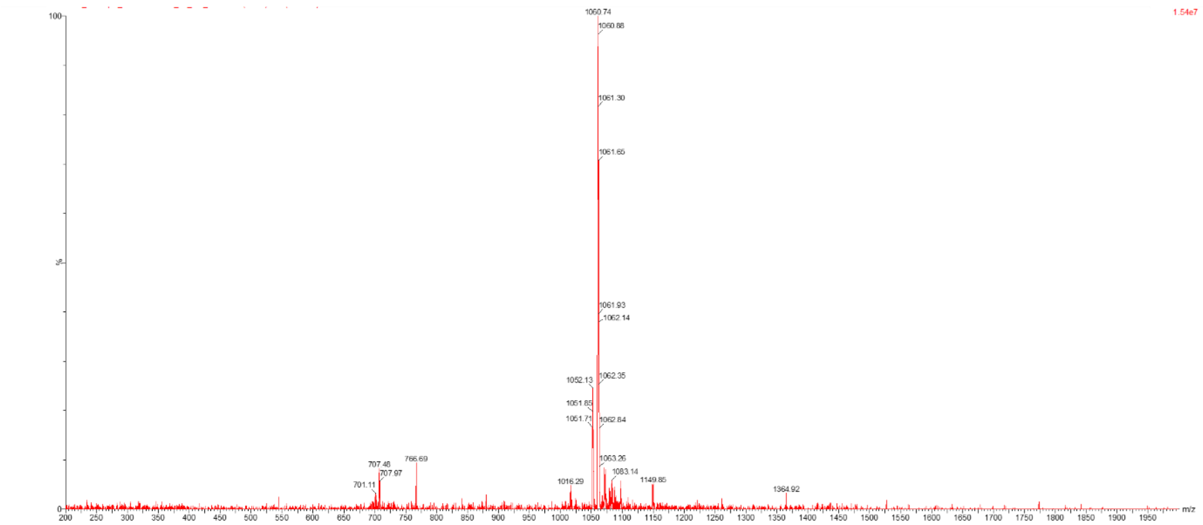

Compound 12: Molecular weight: 1918.23 Da

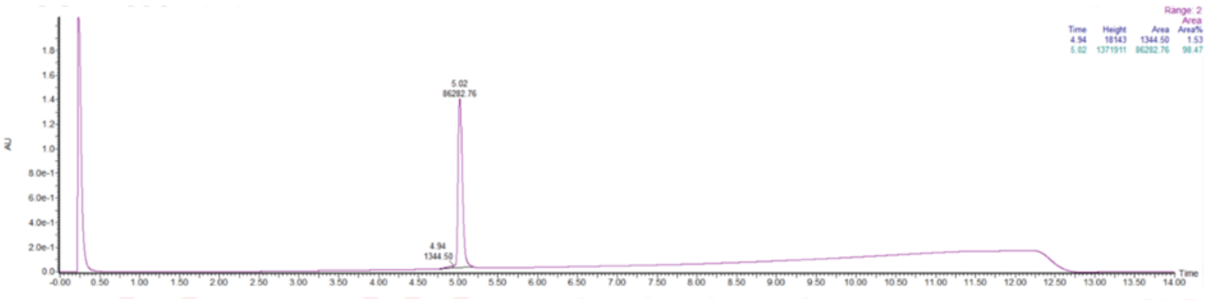

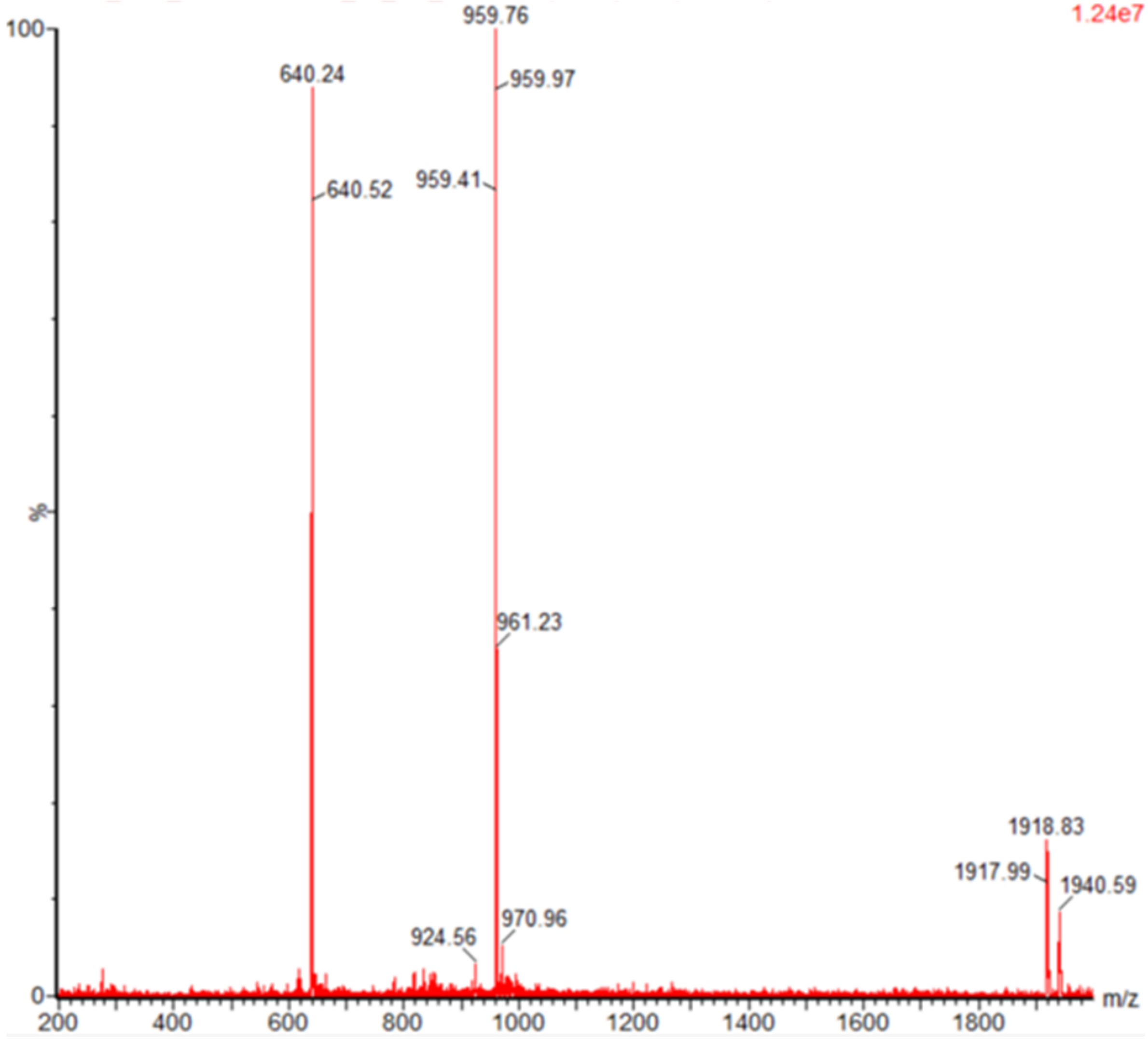

Compound 13: Molecular weight: 2200.4 Da

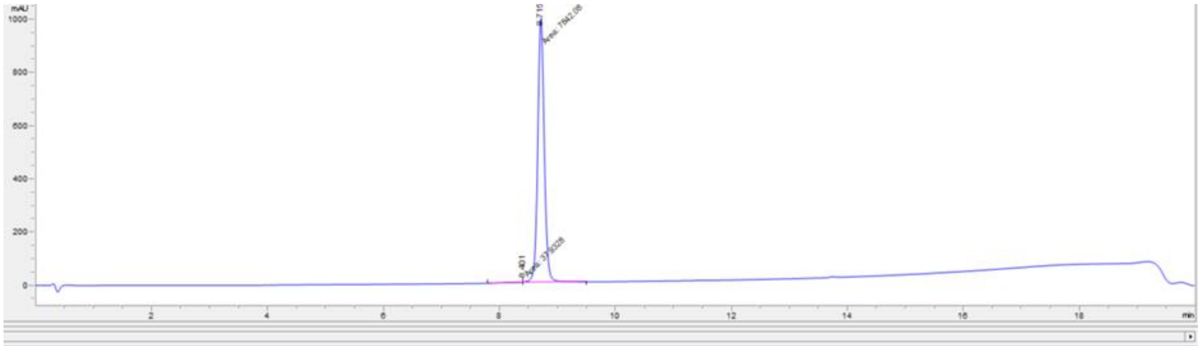

#	Time	Type	Area	Height	Width	Area%	Symmetry
1	8.401	MF	37.9	2.5	0.2554	0.481	1486
2	8.715	FM	7842.1	996.6	0.1312	99.519	0.887

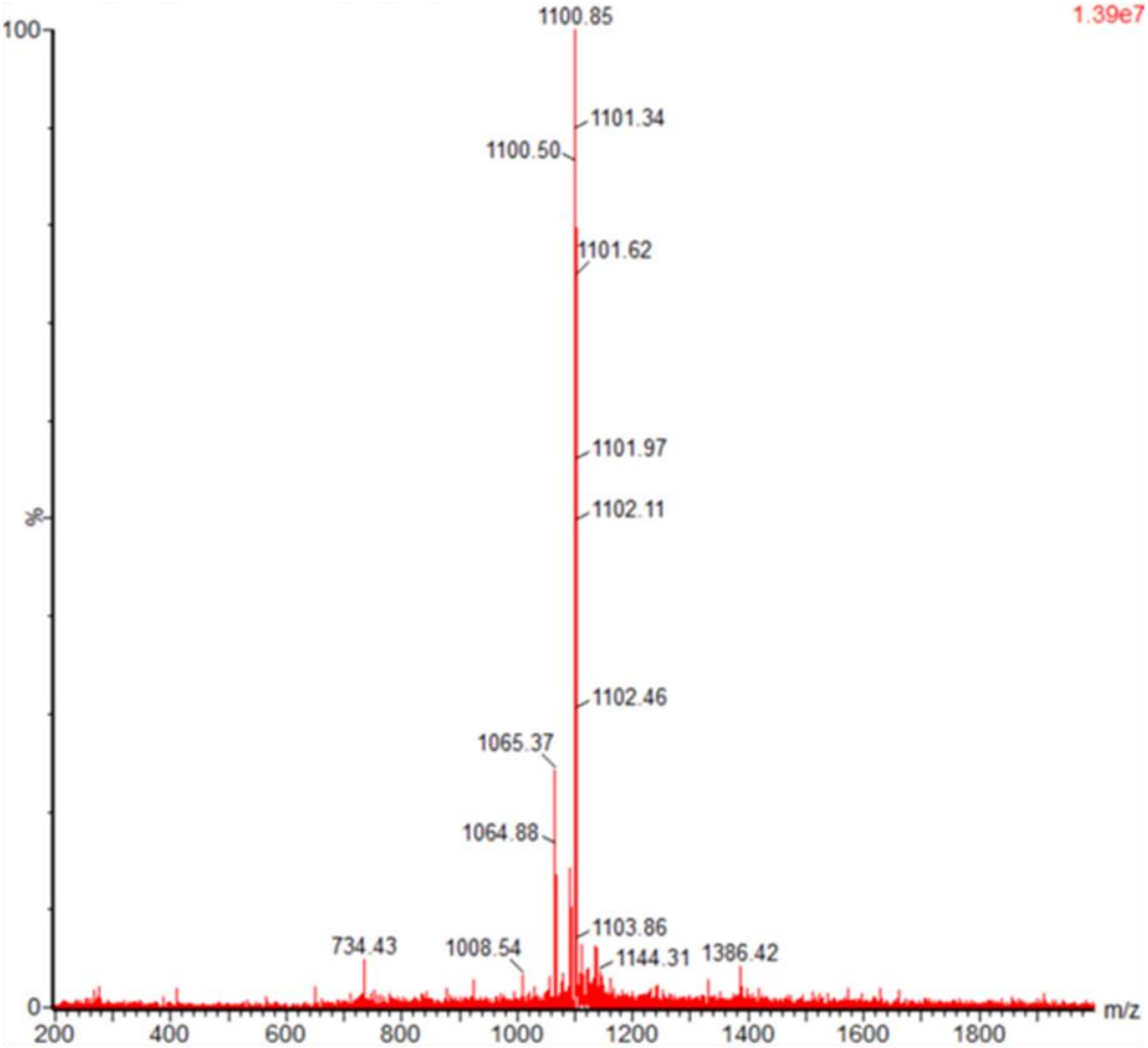

Compound 14: Molecular weight: 2278.5 Da

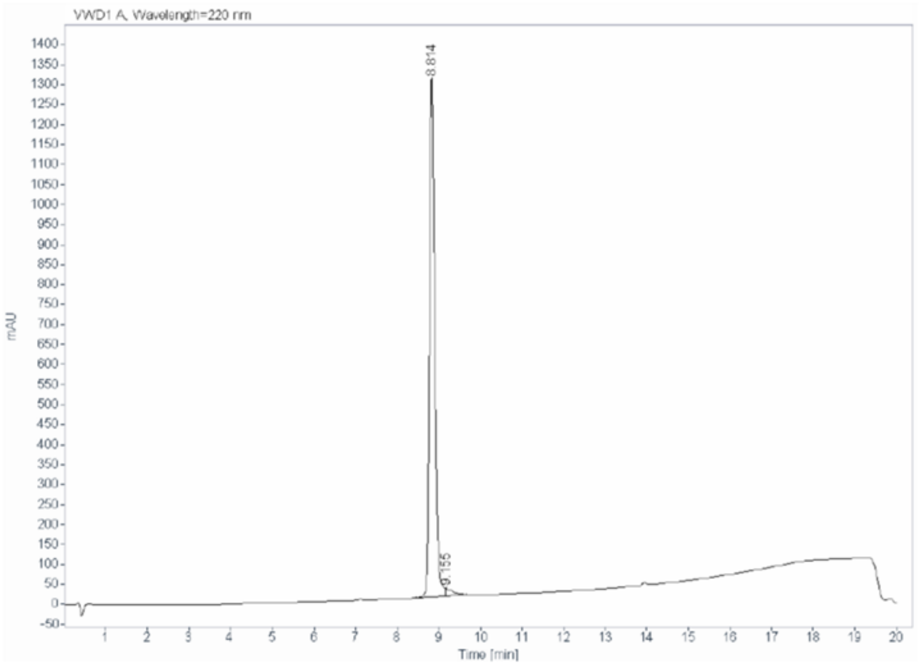

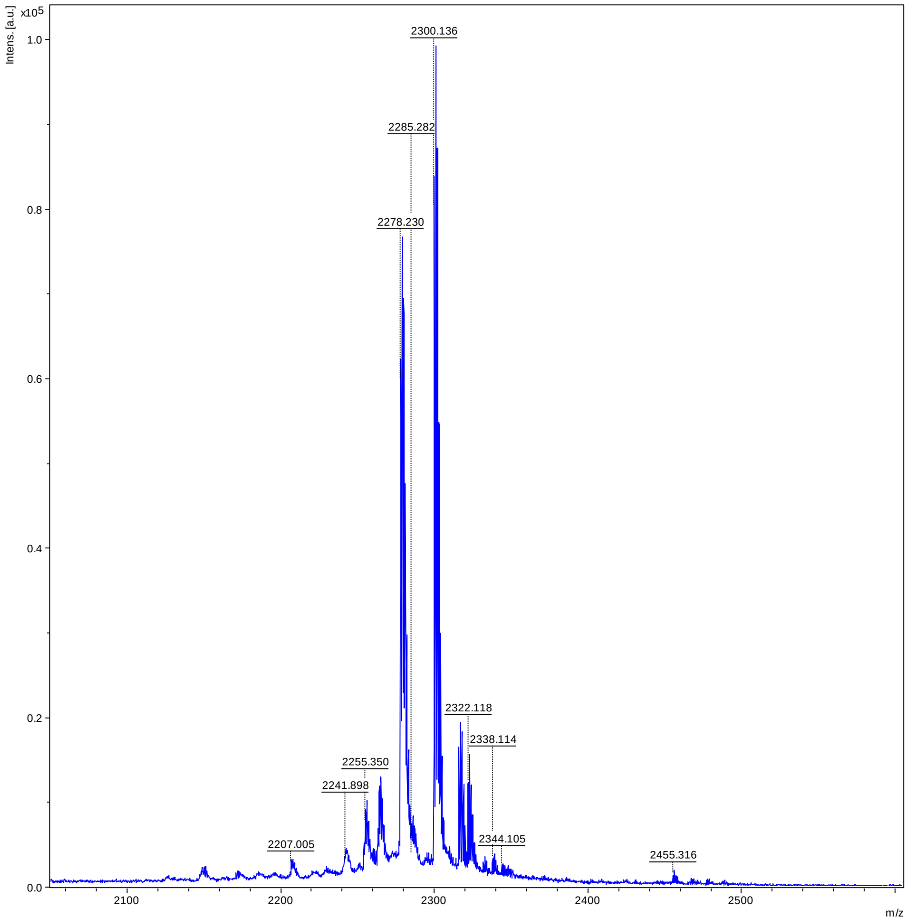

Compound 15: Molecular weight: 2291.6 Da

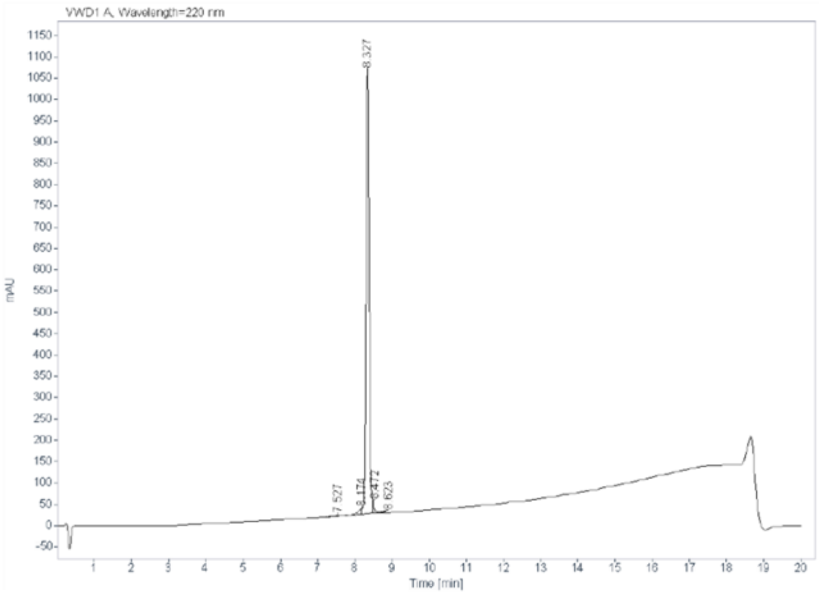

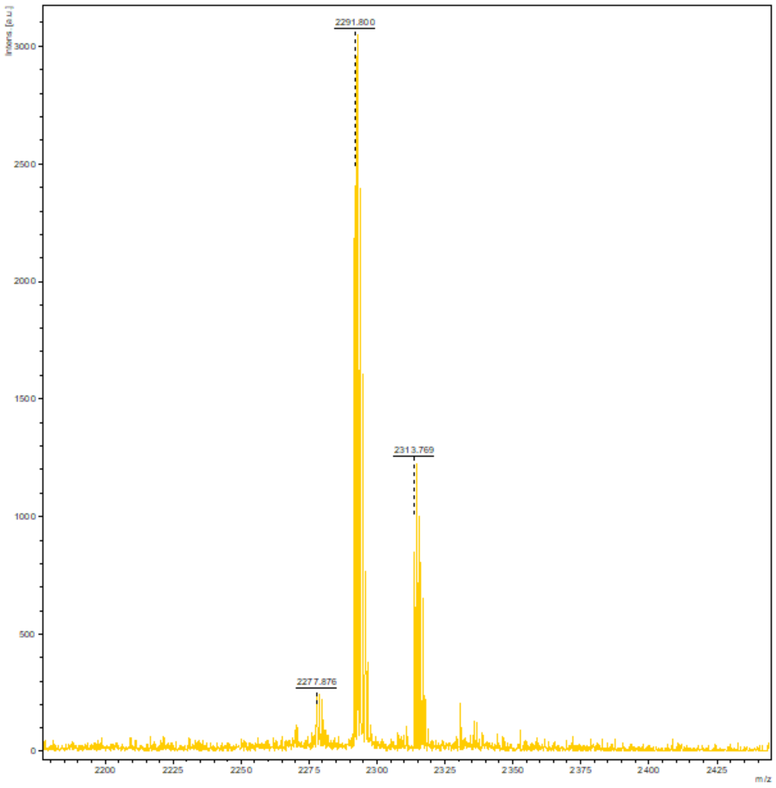

Compound 16: Molecular weight: 2204.6 Da

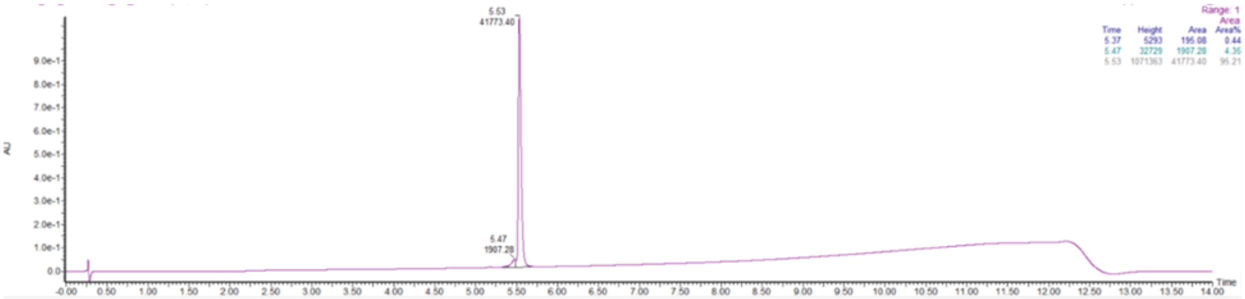

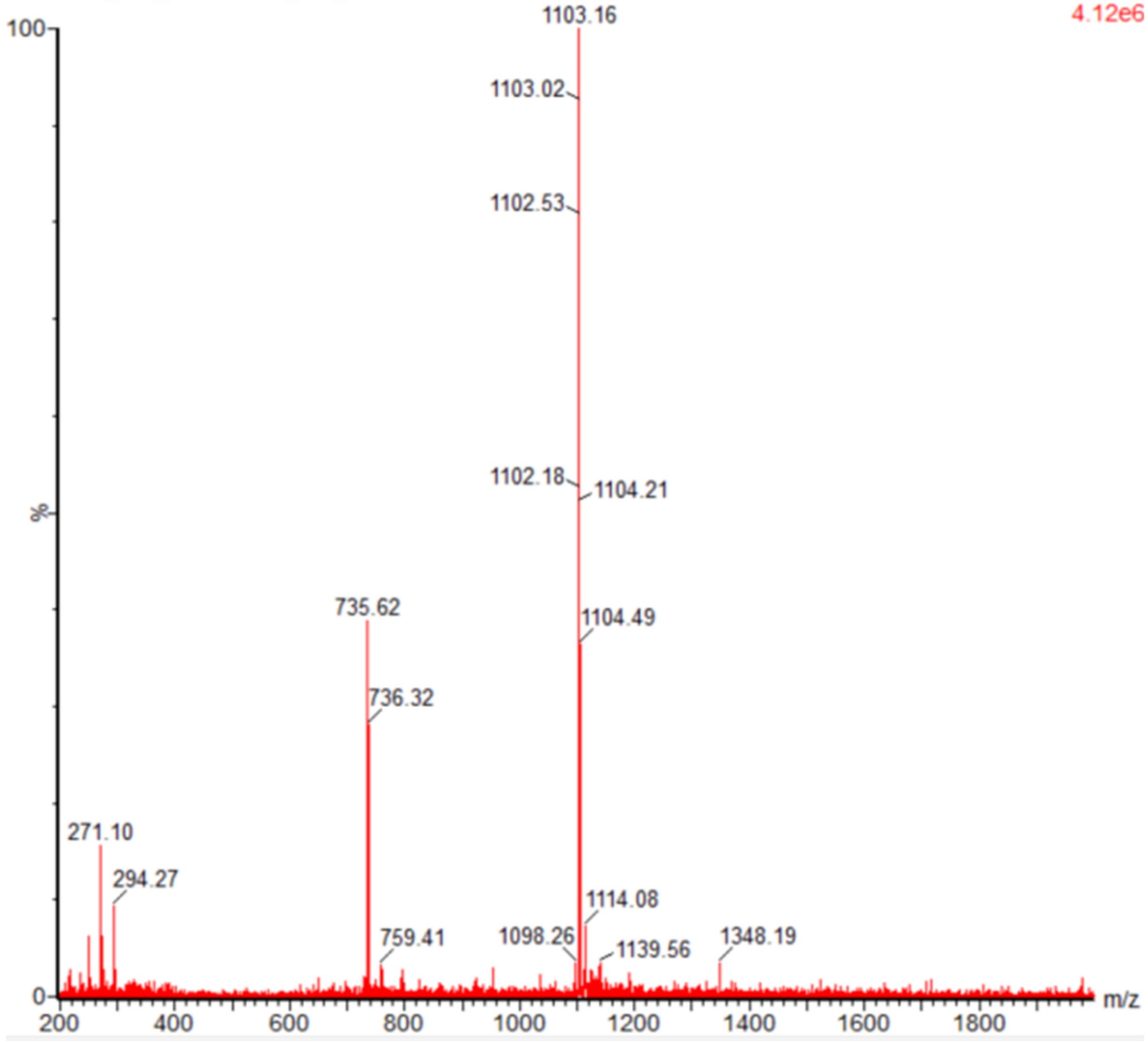

Compound 17: Molecular weight: 1749.1 Da

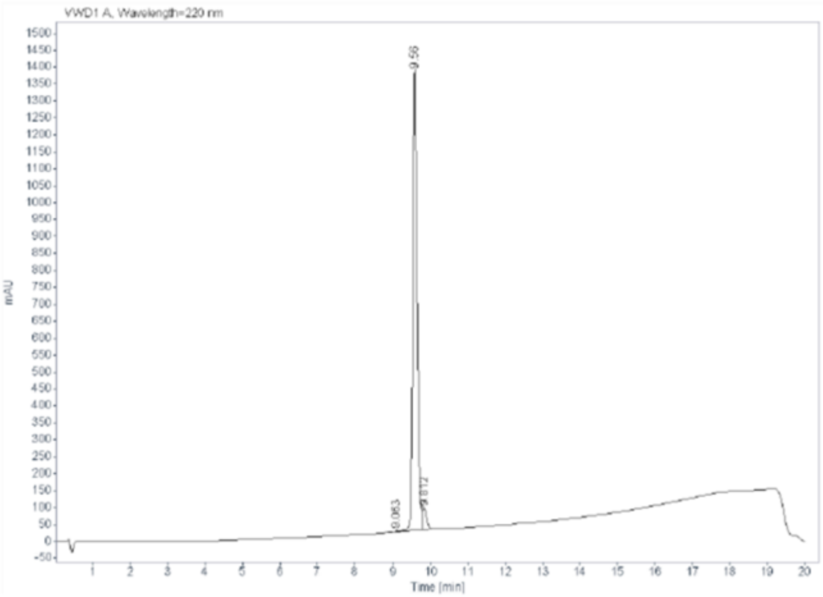

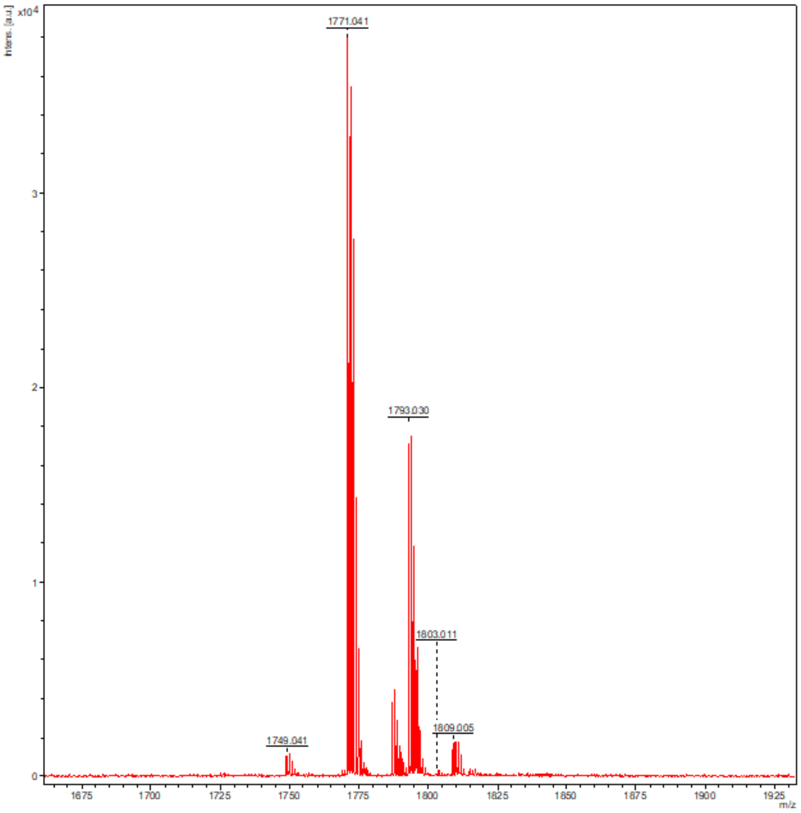

Compound 1a: Molecular weight: 2333.7 Da

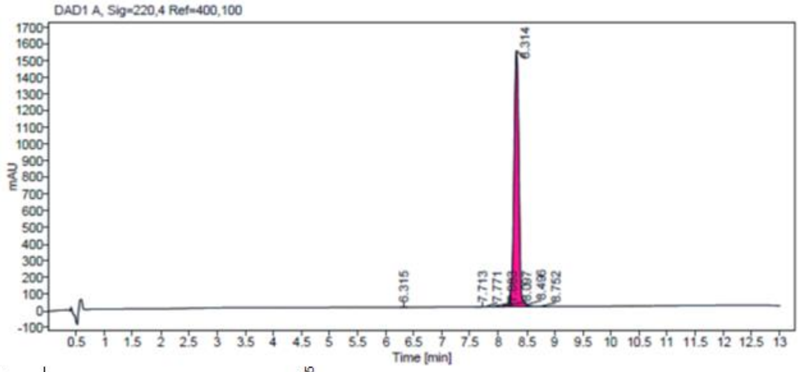

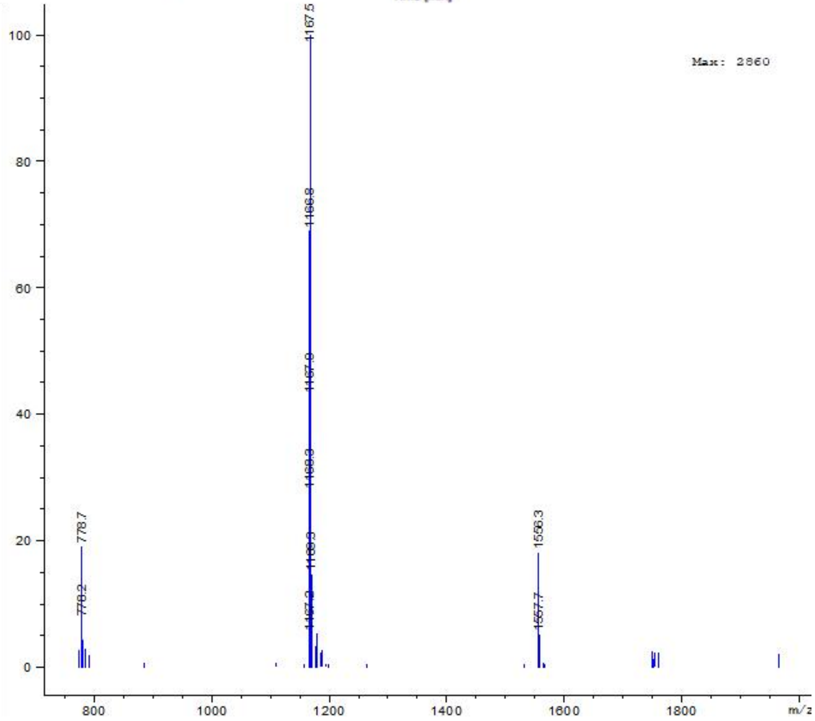

Compound 1b: Molecular weight: 2275.6 Da

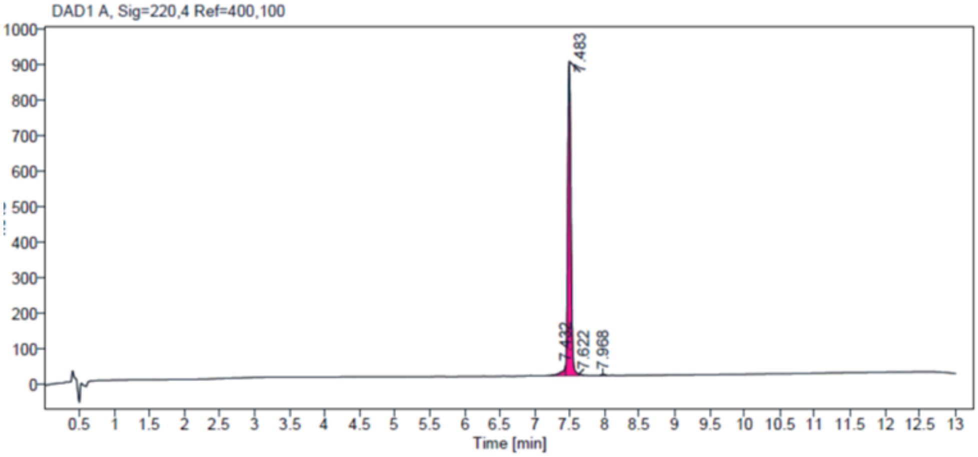

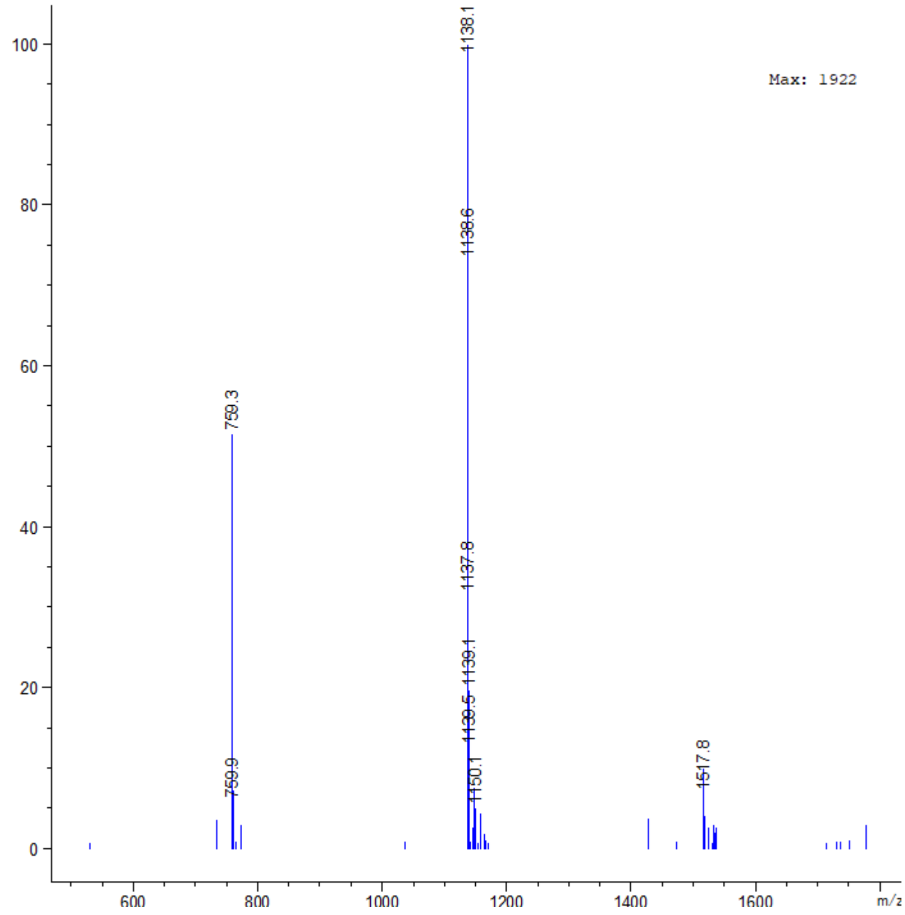

Compound 1c: Molecular weight: 2332.7 Da

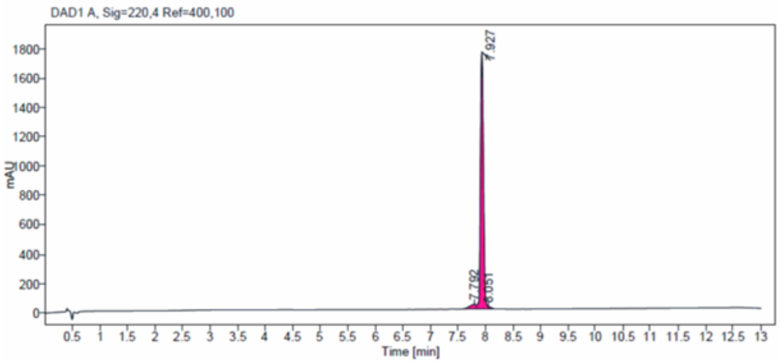

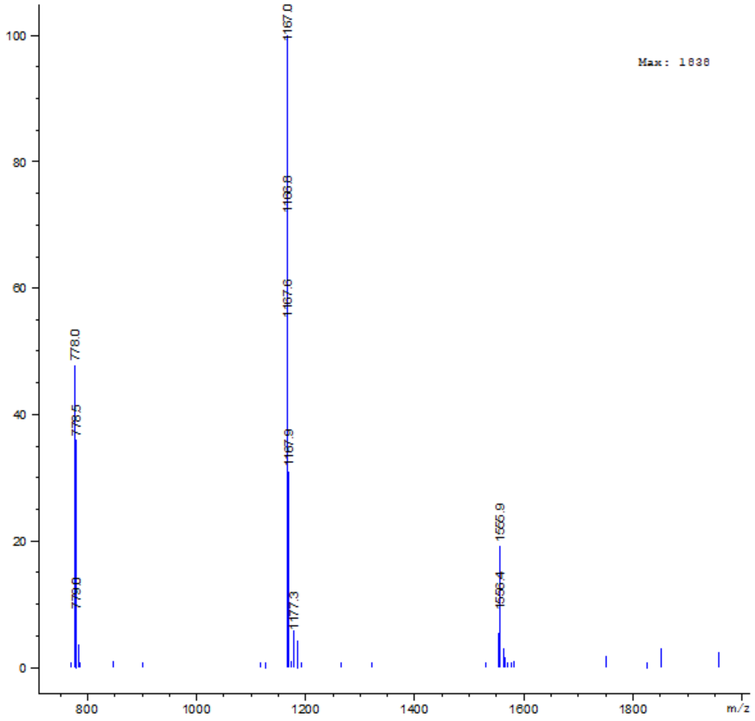

Compound 1d: Molecular weight: 2347.8 Da

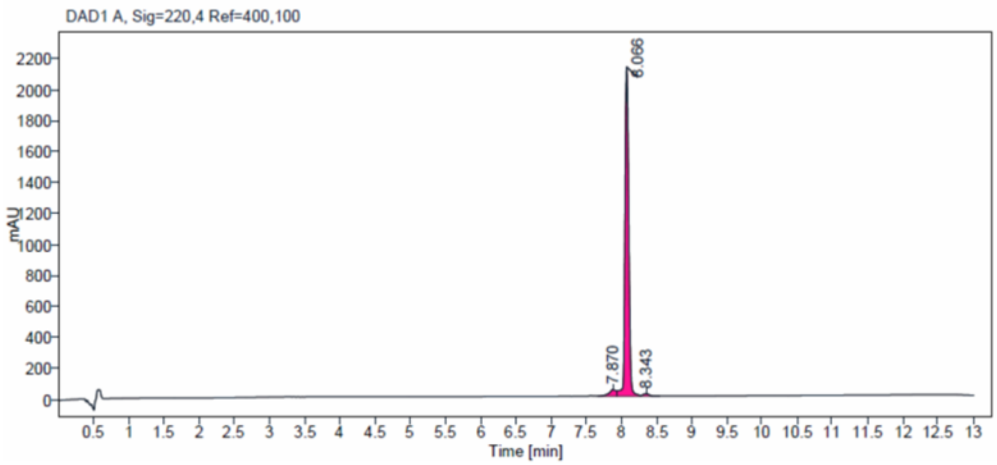

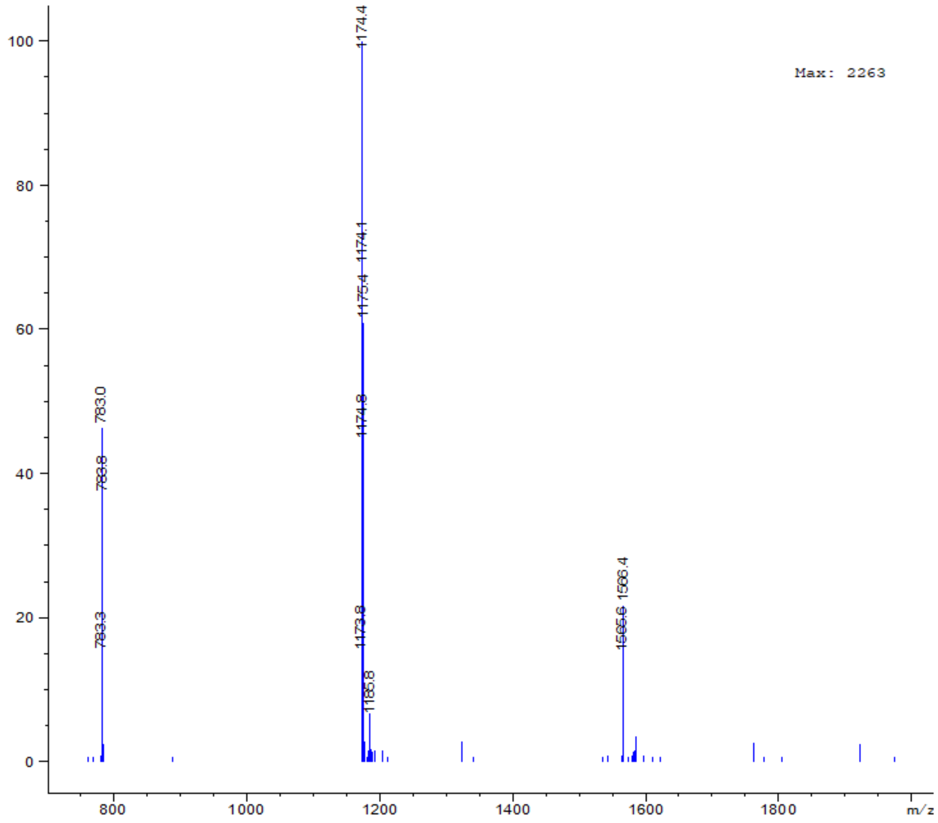

Compound 1e: Molecular weight: 2346.8 Da

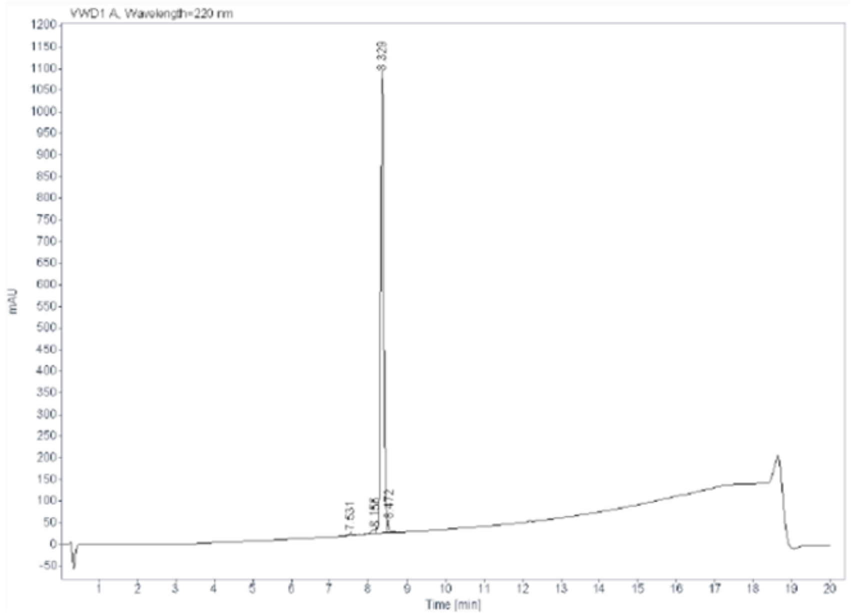

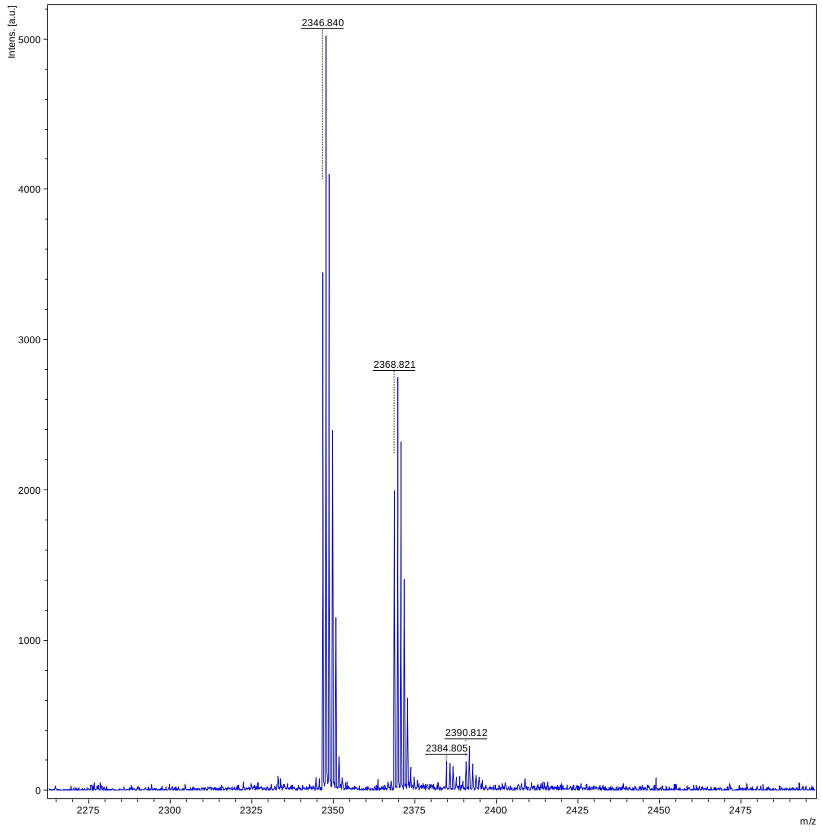

Compound 1f: Molecular weight: 2348.7 Da

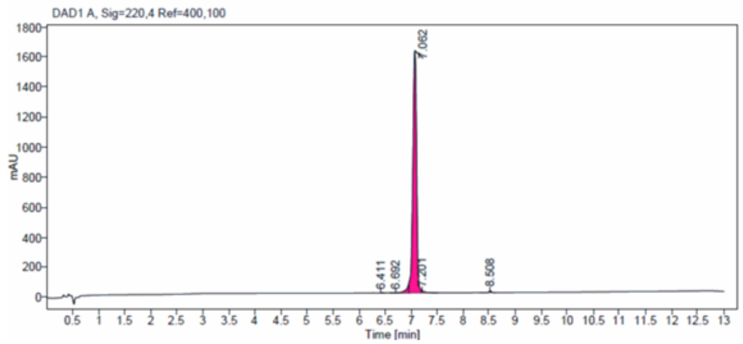

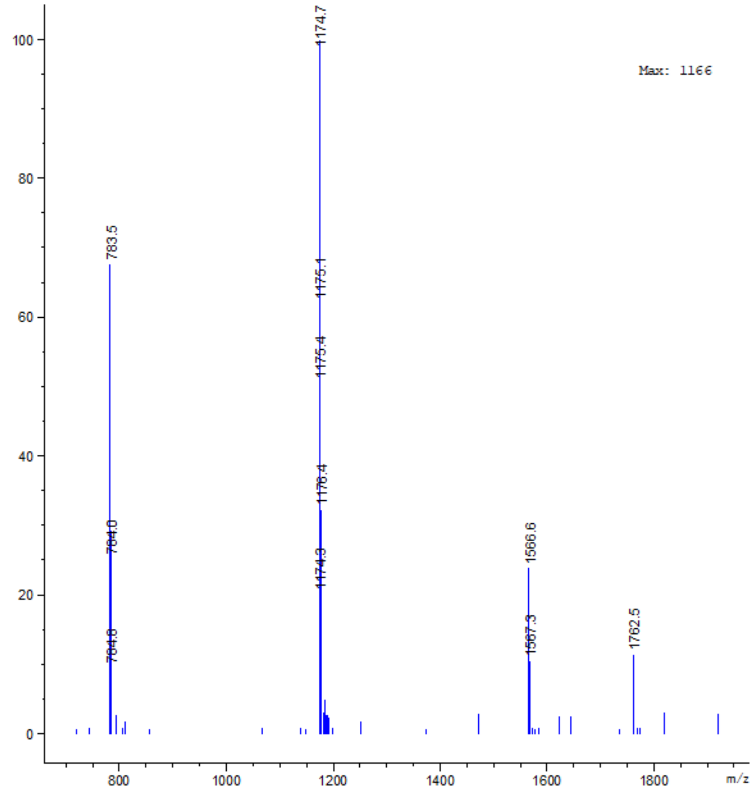

Compound 1g: Molecular weight: 2275.6 Da

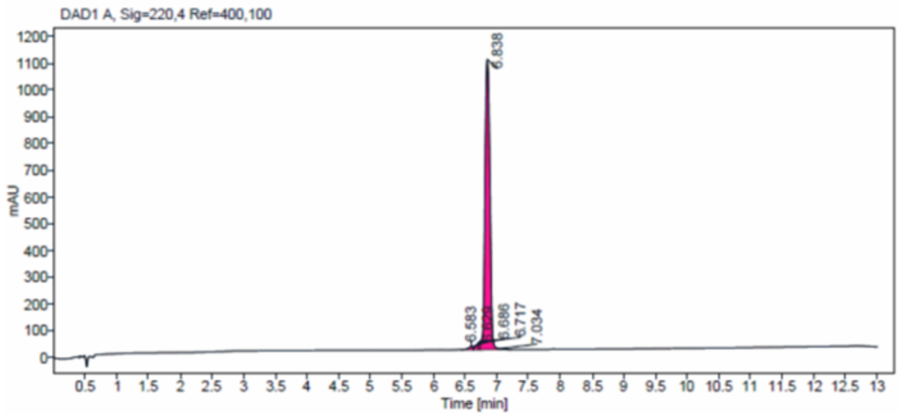

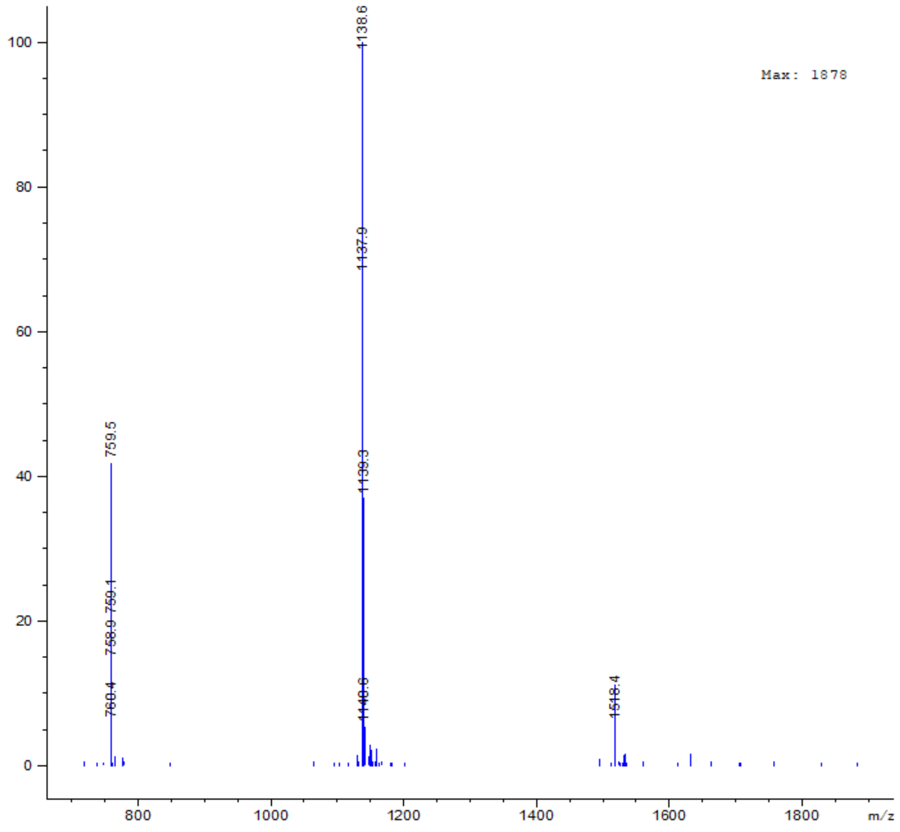

Compound 1h: Molecular weight: 2324.7 Da

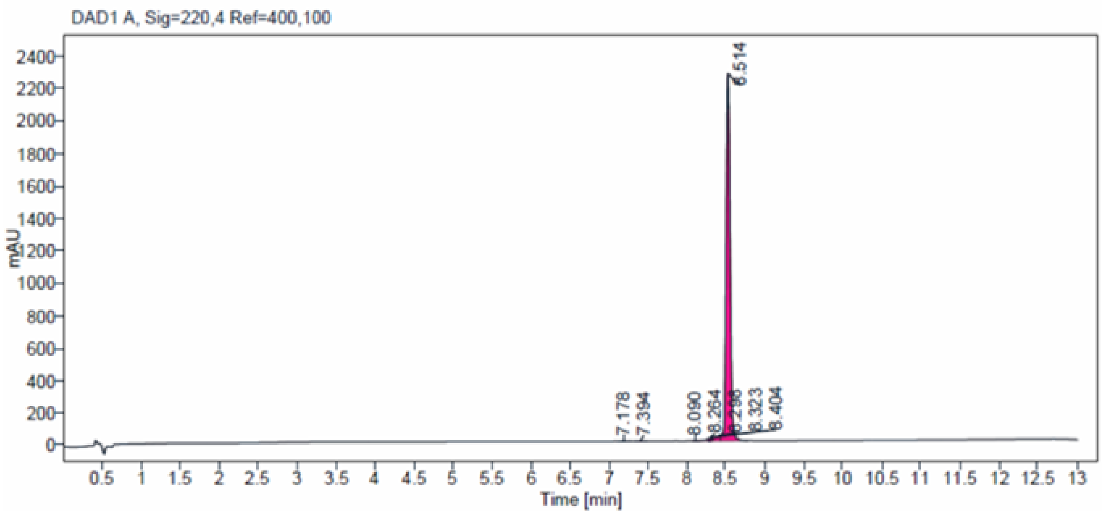

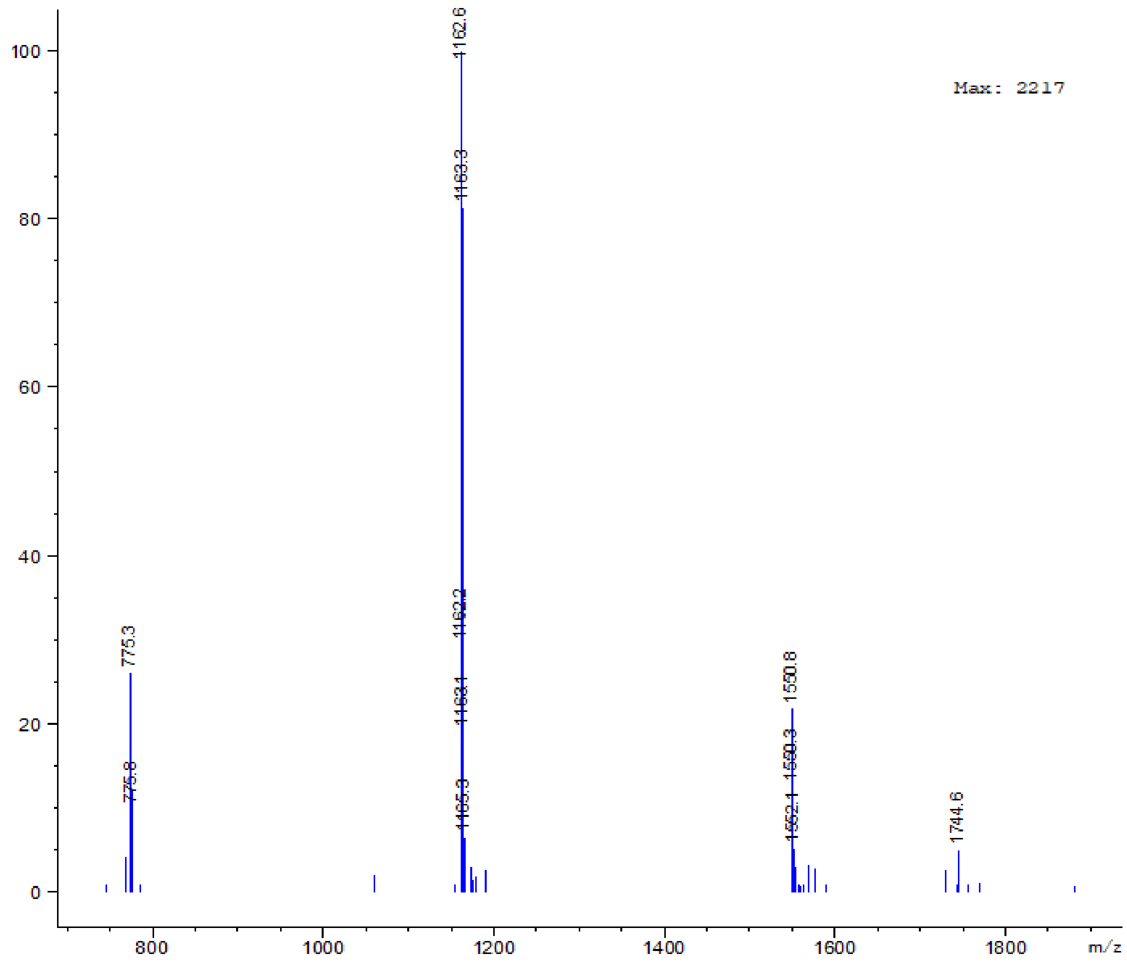

Compound 1i: Molecular weight: 2330.7 Da

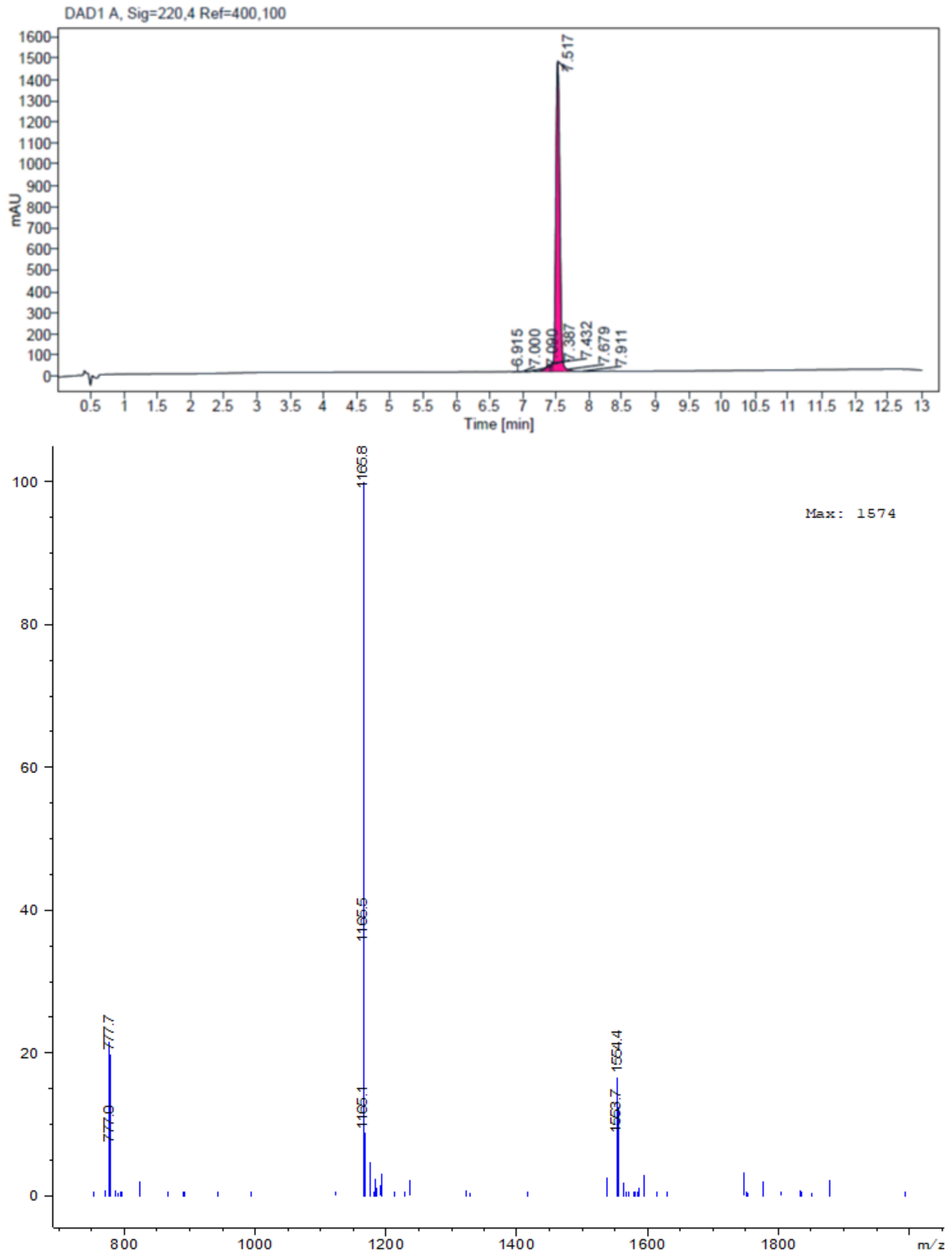

Compound 1j: Molecular weight: 2275.6 Da

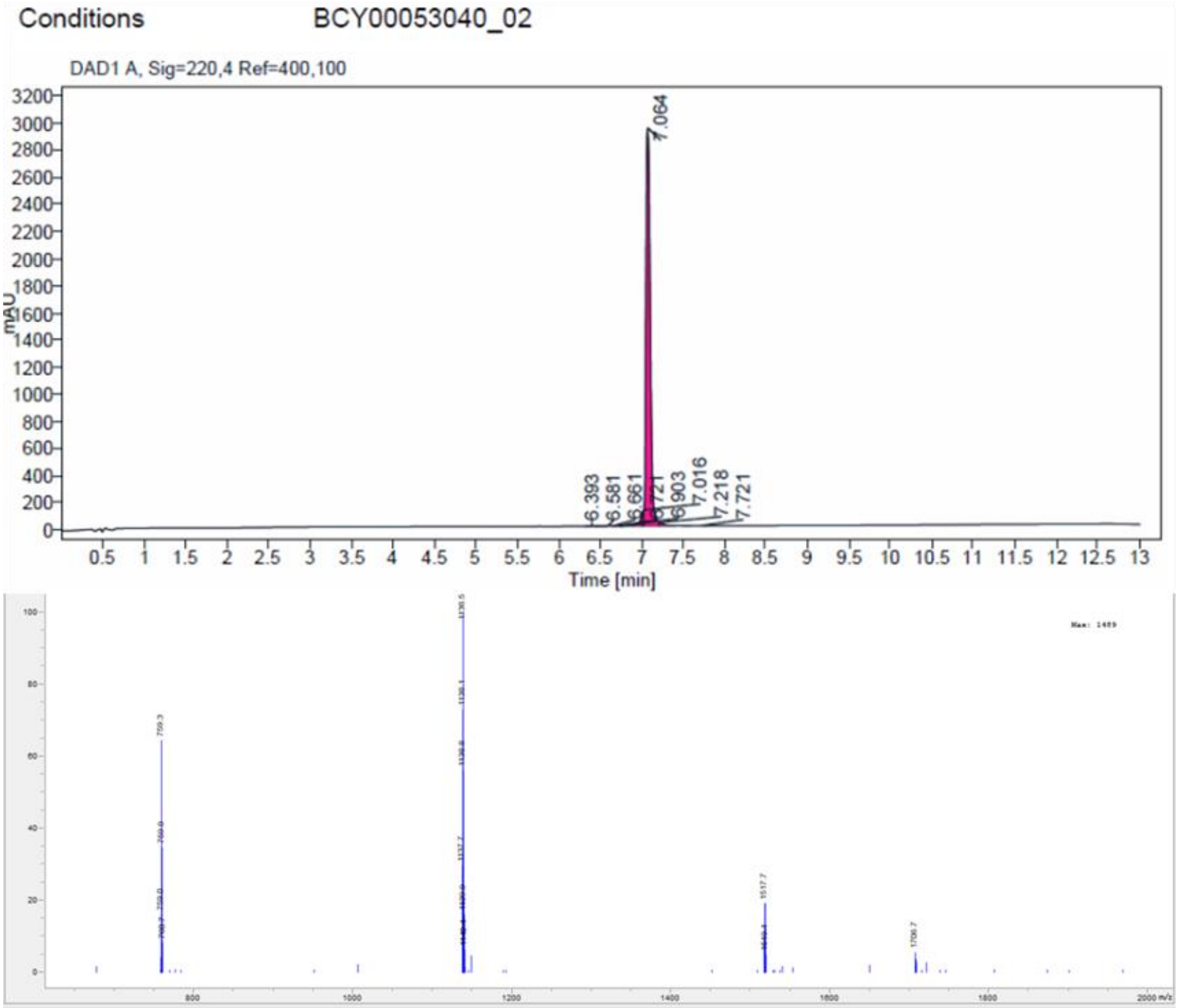

Compound 1k: Molecular weight: 2330.7 Da

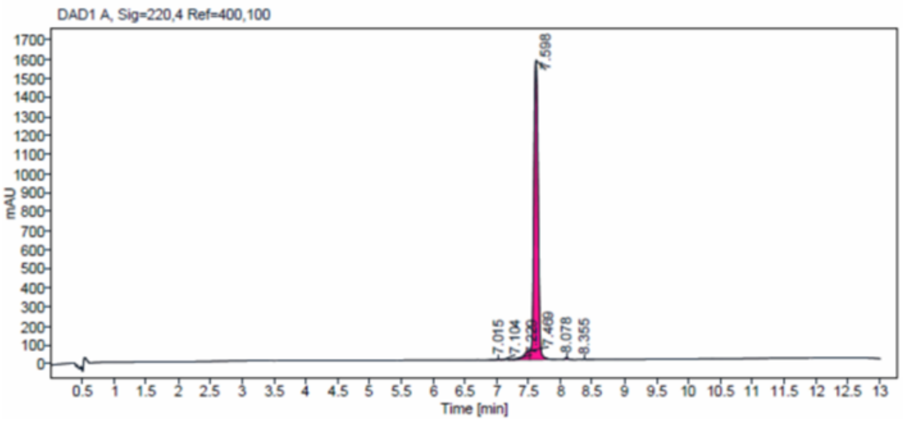

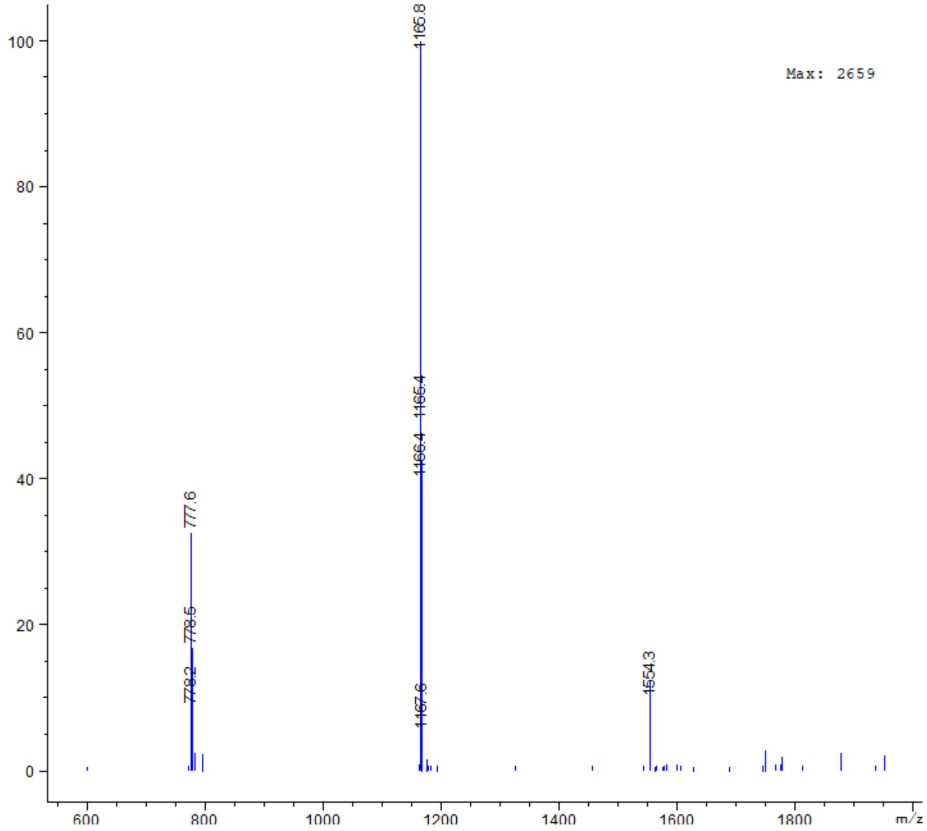

Compound 1l: Molecular weight: 2346.8 Da

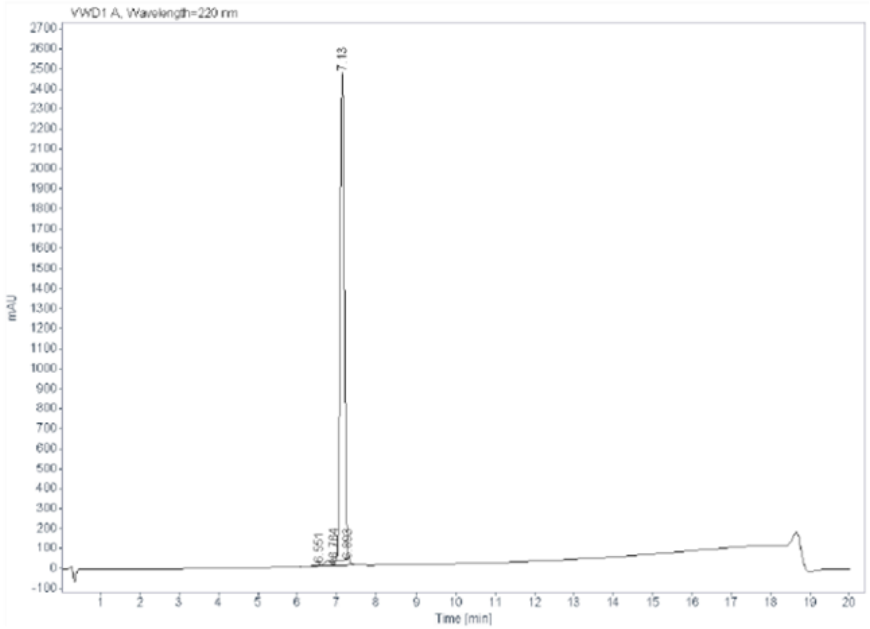

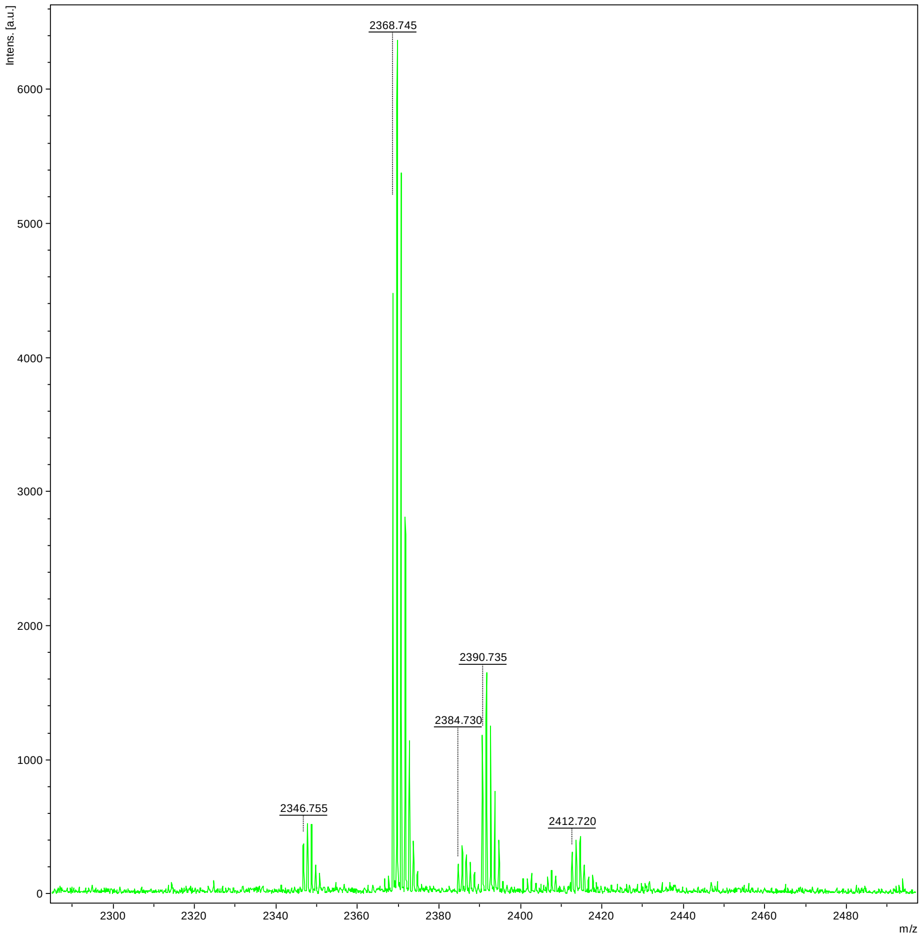

Compound 2a: Molecular weight: 1891.1 Da

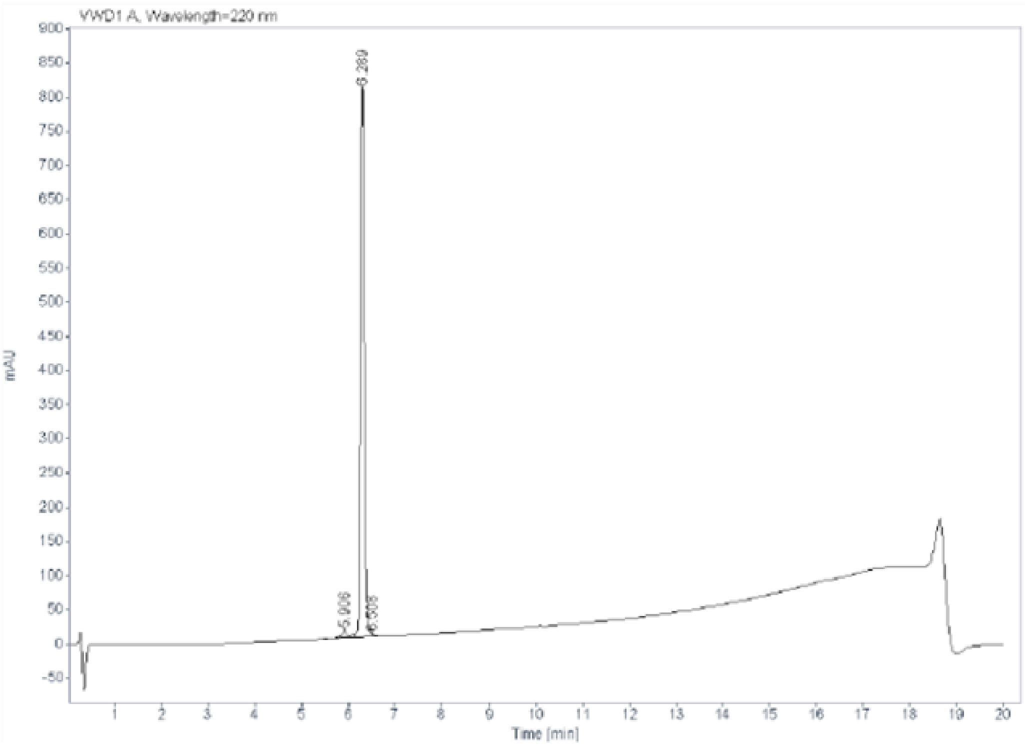

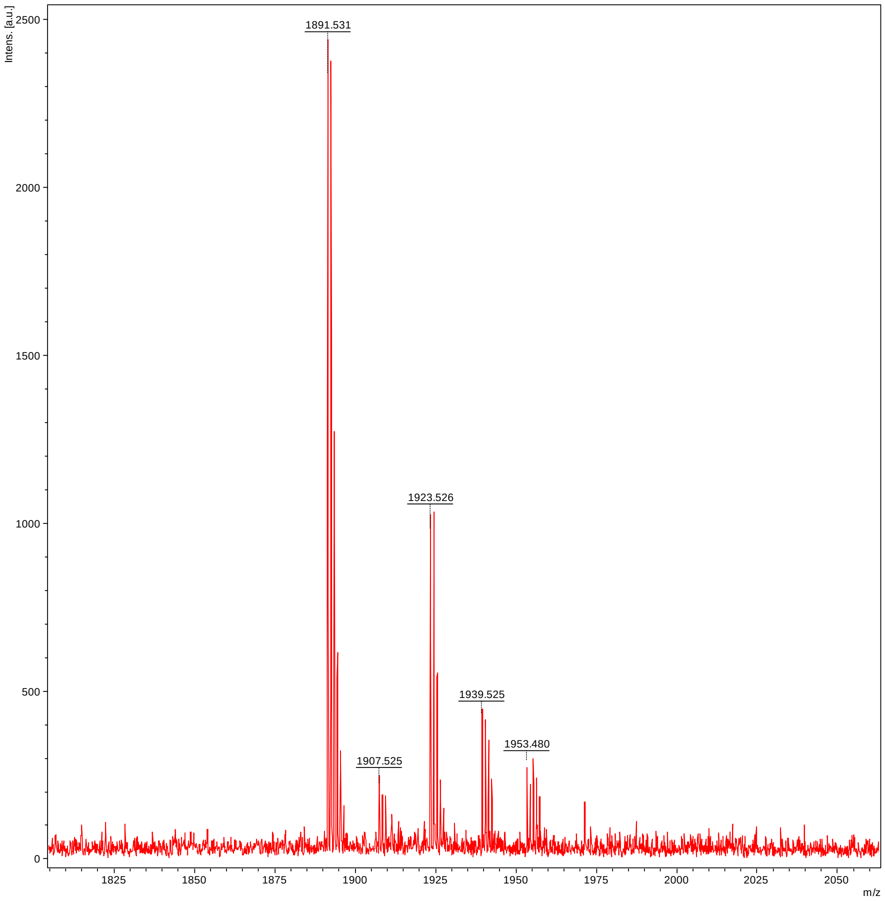

Compound 2b: Molecular weight: 1962.3 Da

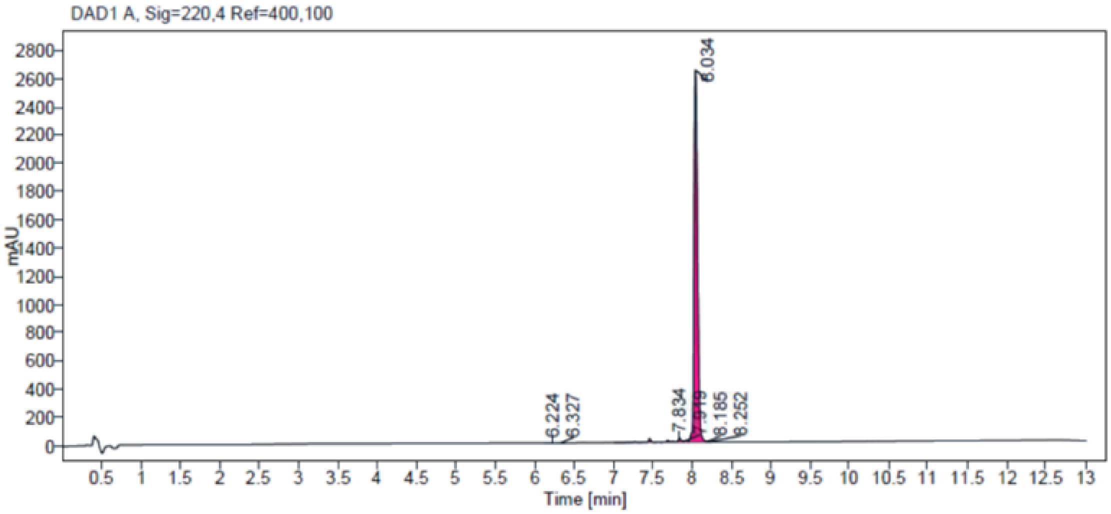

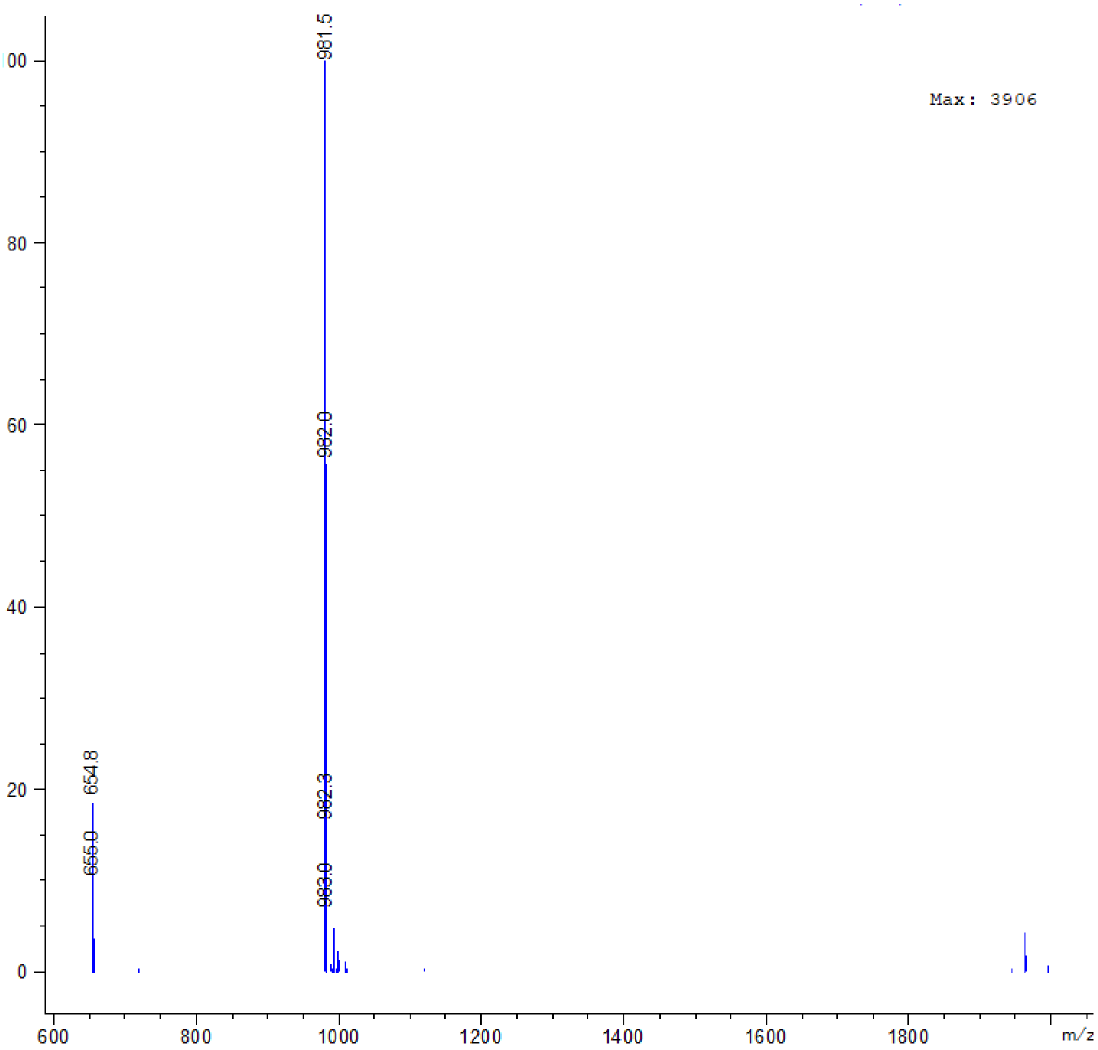

Compound 5a: Molecular weight: 2243.5 Da

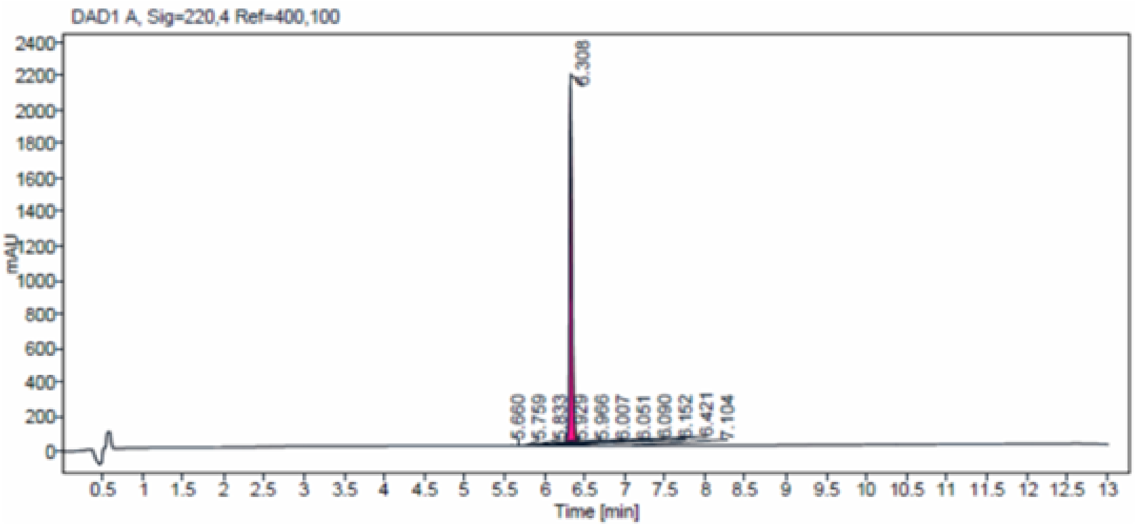

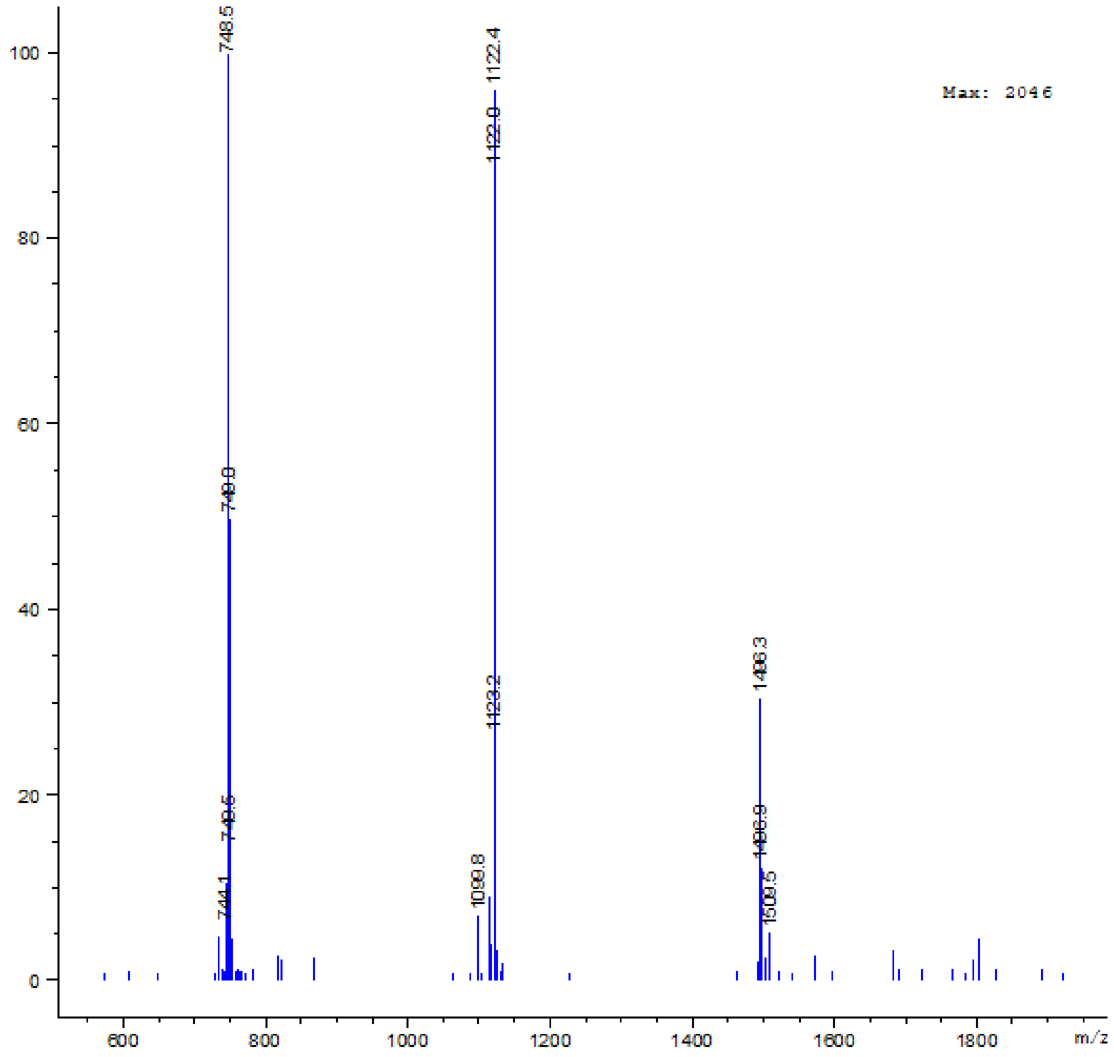

Compound 5b: Molecular weight: 2300.6 Da

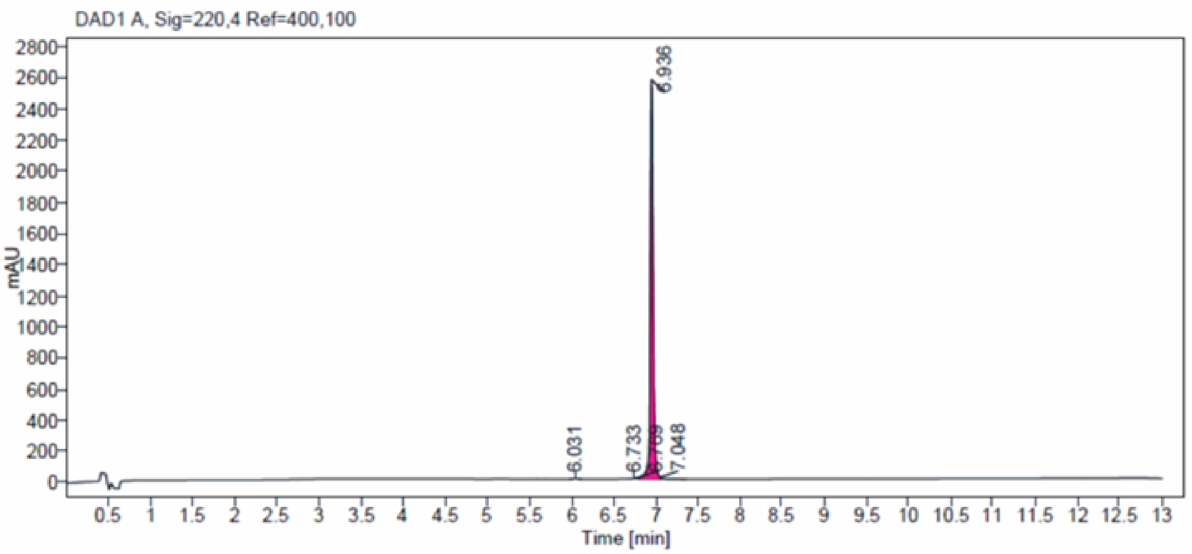

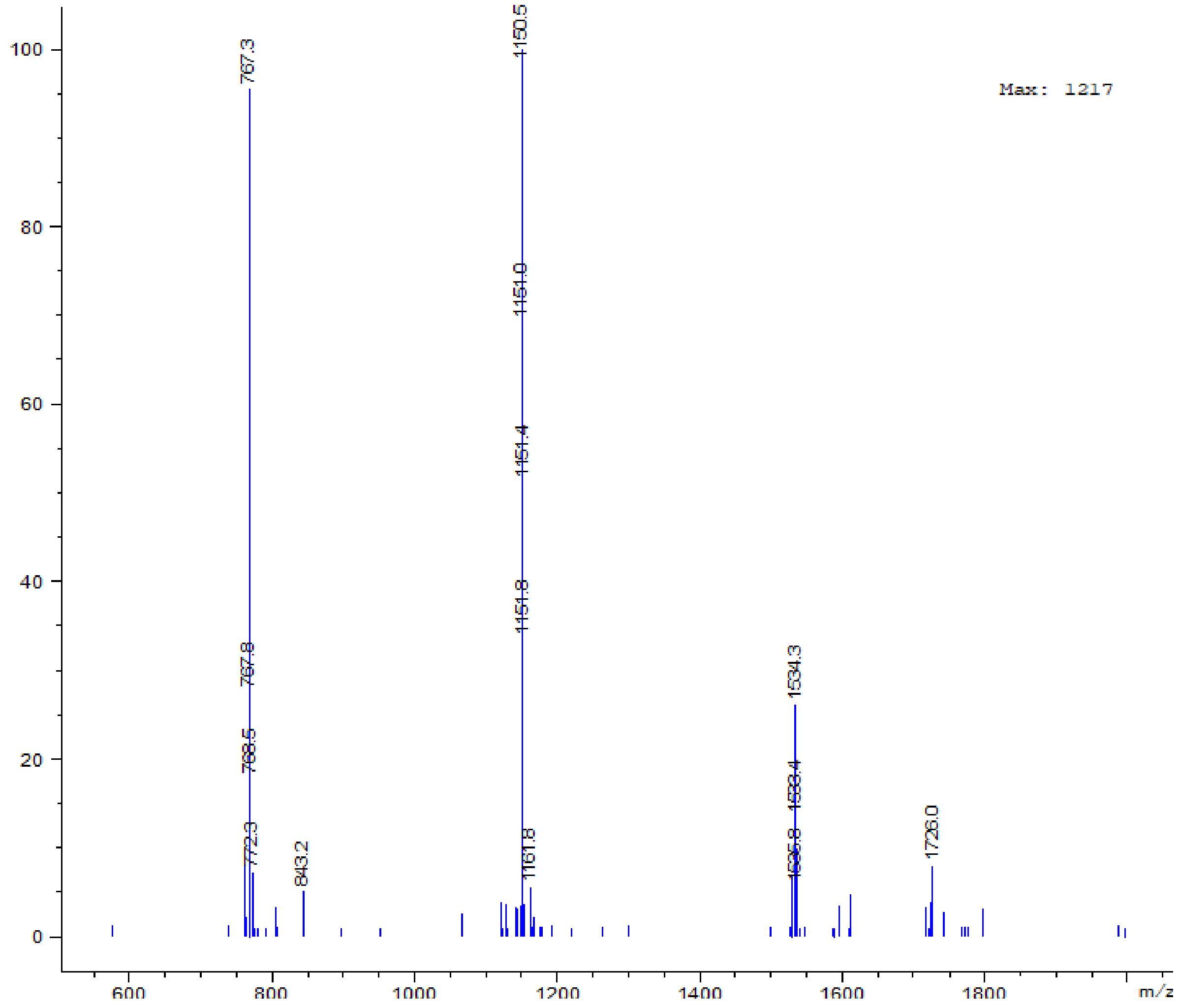

Compound 5c: Molecular weight: 2300.6 Da

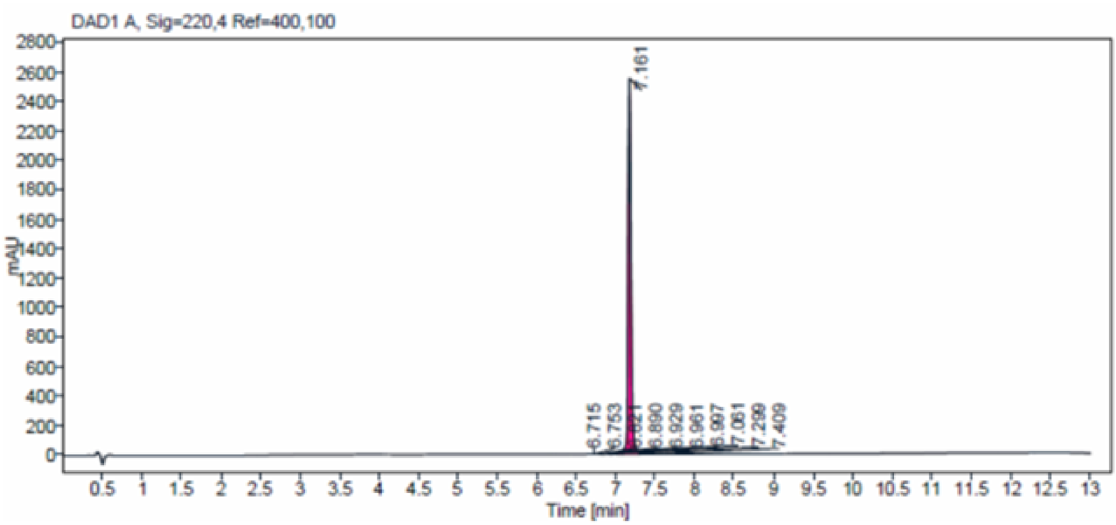

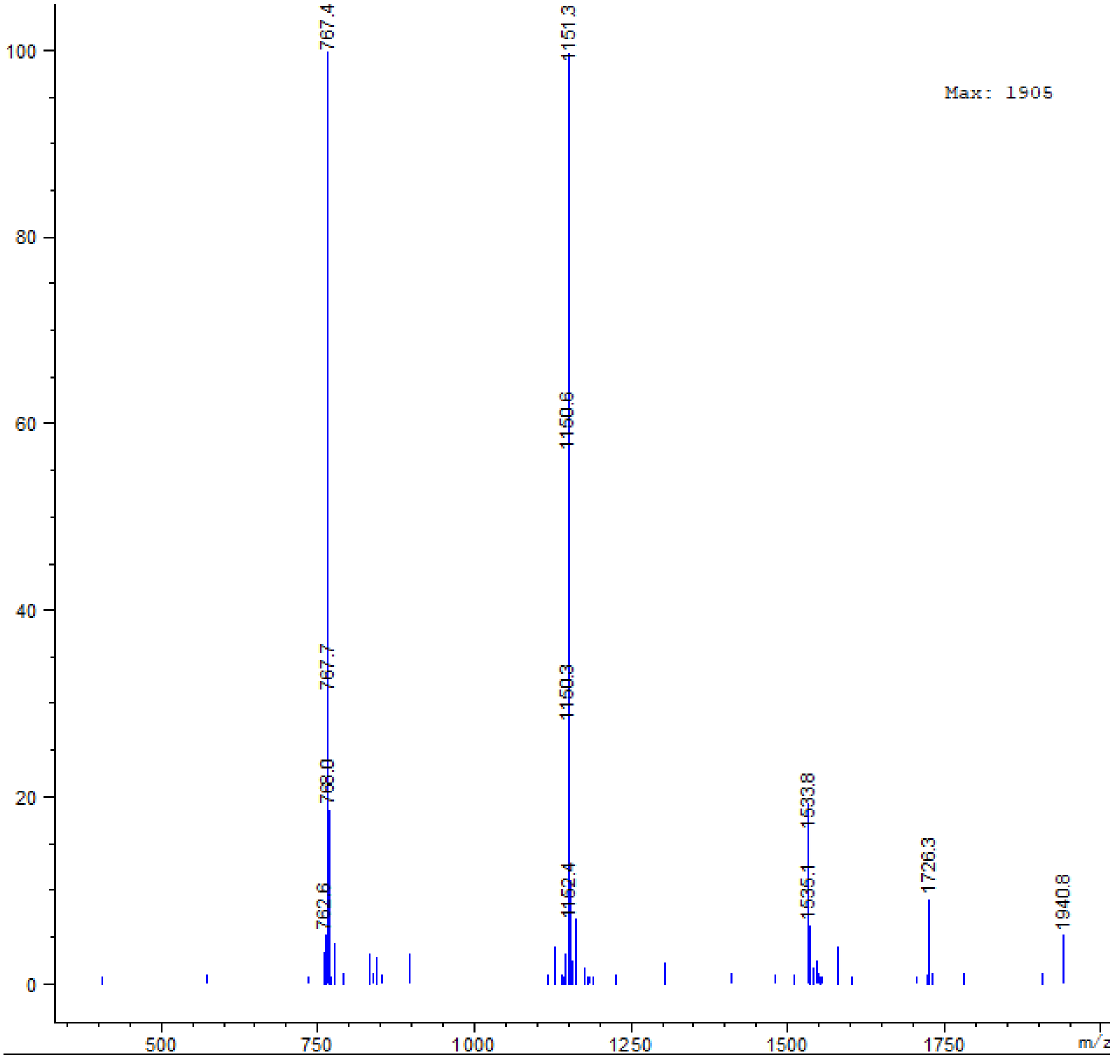

Table S5: Scaffold Structures

Scaffold	Structure[a]
TATB	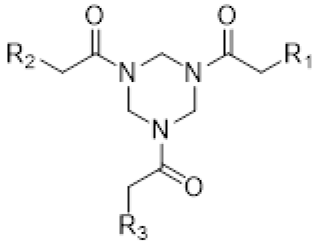
TCTZ	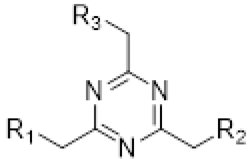
TBAZ	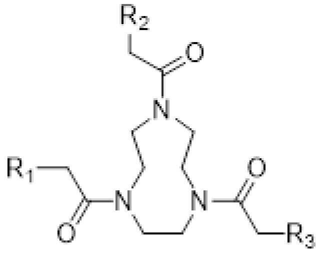
TBCU	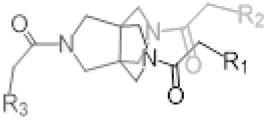
TSTA	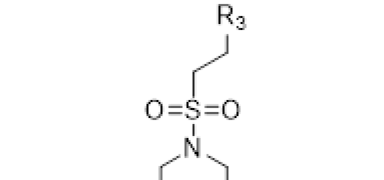

[a]$R_{1\text{-}3}$ represents the position of sulphur atom of the cysteine residue after the completion of the cyclization reaction

References

(1) Baba, T.; Ara, T.; Hasegawa, M.; Takai, Y.; Okumura, Y.; Baba, M.; Datsenko, K. A.; Tomita, M.; Wanner, B. L.; Mori, H. Construction of *Escherichia Coli* K-12 In-frame, Single-gene Knockout Mutants: The Keio Collection. *Mol. Syst. Biol.* **2006**, *2* (1), 2006.0008. https://doi.org/10.1038/msb4100050.

(2) Qiao, S.; Luo, Q.; Zhao, Y.; Zhang, X. C.; Huang, Y. Structural Basis for Lipopolysaccharide Insertion in the Bacterial Outer Membrane. *Nature* **2014**, *511* (7507), 108–111. https://doi.org/10.1038/nature13484.
(3) Gaynor, K. U.; Vaysburd, M.; Harman, M. A. J.; Albecka, A.; Jeffrey, P.; Beswick, P.; Papa, G.; Chen, L.; Mallery, D.; McGuinness, B.; Van Rietschoten, K.; Stanway, S.; Brear, P.; Lulla, A.; Ciazynska, K.; Chang, V. T.; Sharp, J.; Neary, M.; Box, H.; Herriott, J.; Kijak, E.; Tatham, L.; Bentley, E. G.; Sharma, P.; Kirby, A.; Han, X.; Stewart, J. P.; Owen, A.; Briggs, J. A. G.; Hyvönen, M.; Skynner, M. J.; James, L. C. Multivalent Bicyclic Peptides Are an Effective Antiviral Modality That Can Potently Inhibit SARS-CoV-2. *Nat. Commun.* **2023**, *14* (1), 3583. https://doi.org/10.1038/s41467-023-39158-1.
(4) Yasgar, A.; Jadhav, A.; Simeonov, A.; Coussens, N. P. AlphaScreen-Based Assays: Ultra-High-Throughput Screening for Small-Molecule Inhibitors of Challenging Enzymes and Protein-Protein Interactions. *Methods Mol. Biol. Clifton NJ* **2016**, *1439*, 77–98. https://doi.org/10.1007/978-1-4939-3673-1_5.
(5) Luo, Q.; Wang, C.; Qiao, S.; Yu, S.; Chen, L.; Kim, S.; Wang, K.; Zheng, J.; Zhang, Y.; Wu, F.; Lei, X.; Lou, J.; Hennig, M.; Im, W.; Miao, L.; Zhou, M.; Bei, W.; Huang, Y. Surface Lipoprotein Sorting by Crosstalk between Lpt and Lol Pathways in Gram-Negative Bacteria. *Nat. Commun.* **2025**, *16* (1), 4357. https://doi.org/10.1038/s41467-025-59660-y.
